# BiCl_3_-catalyzed green synthesis of 4-hydroxy-2-quinolone analogues under microwave irradiation†

**DOI:** 10.1039/d3ra05289c

**Published:** 2023-09-21

**Authors:** Yousra Ouafa Bouone, Abdeslem Bouzina, Rayene Sayad, Abdelhak Djemel, Farouk Benaceur, Abdelhalim Zoukel, Malika Ibrahim-Ouali, Nour-Eddine Aouf, Fouzia Bouchareb

**Affiliations:** a Laboratory of Applied Organic Chemistry, Bioorganic Chemistry Group, Department of Chemistry, Sciences Faculty, Badji-Mokhtar – Annaba University Box 12 23000 Annaba Algeria abdeslem.bouzina@univ-annaba.dz bouzinaabdeslem@yahoo.fr; b Laboratory of Applied Organic Chemistry, Synthesis of Biomolecules and Molecular Modelling Group, Department of Chemistry, Sciences Faculty, Badji-Mokhtar – Annaba University Box 12 23000 Annaba Algeria; c Research Unit in Medicinal Plants, URPM, Research Center of Biotechnology, CRBt 3000 Laghouat 25000 Constantine Algeria; d Technical Platform of Physico-Chemical Analysis (PTAPC-Laghout-CRAPC), University of Laghouat Laghouat 03000 Algeria; e Aix Marseille Univ, CNRS, Centrale Marseille, iSm2 F-13397 Marseille France; f Faculty of Sciences and Technology, Department of Chemistry, Chadli Bendjedid – EL Tarf University P.O. Box: 73 El Tarf 36000 Algeria

## Abstract

Traditional chemical synthesis, which involves the use of dangerous protocols, hazardous solvents, and toxic products and catalysts, is considered environmentally inappropriate and harmful to human health. Bearing in mind its numerous drawbacks, it has become crucial to substitute conventional chemistry with green chemistry which is safer, more ecofriendly and more effective in terms of time and selectivity. Elaborating synthetic protocols producing interesting new compounds using both microwave heating and heterogeneous non-toxic catalysts is acknowledged as a green approach that avoids many classical chemistry-related problems. In the current study, β-enaminones were used as precursors to the synthesis of modified 4-hydroxy-2-quinolone analogues. The synthesis was monitored in a benign way under microwave irradiation and was catalyzed by bismuth chloride III in an amount of 20 mol%. This method is privileged by using a non-corrosive, non-toxic, low-cost and available bismuth Lewis acid catalyst that has made it more respectful to the demands of green chemistry. The synthesized compounds were obtained in moderate to good yields (51–71%) and were characterized by ^1^H, ^13^C NMR, and IR spectroscopy as well as elemental analysis. Compound 5i was subjected to a complete structural elucidation using the X-ray diffraction method, and the results show the obtention of the enolic tautomeric form.

## Abstract 2

Traditional chemical synthesis, which involves the use of dangerous protocols, hazardous solvents, and toxic products and catalysts, is considered environmentally inappropriate and harmful to human health.

## Graphical abstract

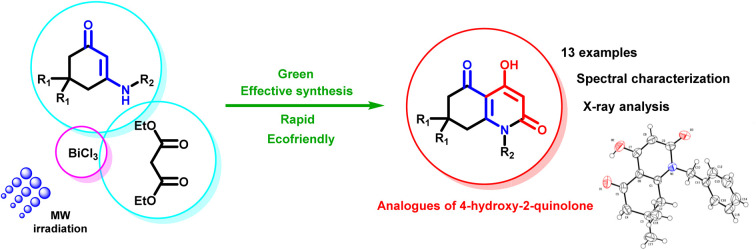

## Introduction

Microwave-assisted synthesis has constituted a remarkable revolution in the field of green chemistry and the organic synthesis of bioactive compounds.^1^ The introduction of microwave irradiation into organic chemistry laboratories has helped to overcome many problems related to traditional synthesis, including high reaction times, low yields, and poor selectivity that can directly affect the effectiveness of synthetic protocols. Using microwave radiation as a source of heat increased yields and shortened reaction times from several hours to a few minutes or seconds. Furthermore, microwave heating plays a crucial role in decreasing toxic byproducts and avoiding the use of hazardous solvents and harsh reaction conditions that are greatly used in conventional chemistry methods such as refluxing.

Microwave-heating effectiveness relies on the fact that the reaction materials themselves absorb microwave electromagnetic energy and convert it into thermal energy, resulting in homogeneous and equally partitioned heat all over the reaction constituents, unlike traditional heating in which the high temperature is superficially conducted to the external surface of the material.^2^

In addition to the use of microwaves as a green method that decreases reaction times, heterogeneous catalysts have also triggered the interest of scientists with regard to their high utility in generating new products in a rapid and selective manner.^3,4^ Microwave activation, which consists of deep heating of the reaction components, combined with solid catalysis, which has the advantages of reusability, recoverability, and high selectivity, is recognized nowadays as an effective tool in the synthesis of different important heterocyclic systems, such as imidazole,^5^ acridinedione,^6^ quinazolinone,^7^ dihydroquinazolinone,^8^ pyridine,^9^ dihydropyridine,^10^ and quinolone.^11^

**Scheme 1:**
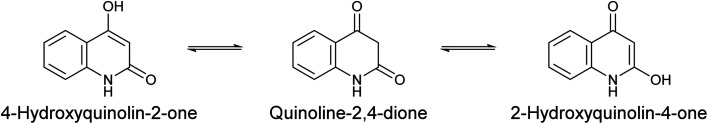
Main tautomeric forms of 4-hydroxyquinolin-2-one.

The chemistry of heterocycles constitutes an important branch of the field of drug design and the development of new biologically active compounds. Many natural and synthetic active products bear a heterocycle within their structures; these molecules are recognized for their vast number of applications in the medical field, exhibiting various beneficial pharmacological activities.^12–20^ A well-known class of heterocycles, 4-hydroxyquinolin-2-one and its tautomers (Scheme 1),^21^ are of great interest in both chemical and medicinal domains. In 2017, the number of described molecules containing a 4-hydroxyquinolin-2-one skeleton reached 14 thousand including nearly 7 thousand compounds that had been subjected to bioactivity studies.^22^

4-Hydroxyquiolin-2-ones found a large spectrum of applications as therapeutic agents presenting antibacterial,^23–25^ anticancer,^26,27^ antiproliferative,^28^ analgesic,^29–31^ antiallergenic,^32^ and antitubercular activities.^33^ They were also described as antagonists of cannabinoid type 2 receptor CB2R,^34^ and modulators of glycogen synthase kinase GSK-3.^35^

Due to their wide range of biological applications, many synthetic routes leading to 4-hydroxy-2-quinolones and related analogues have been reported in the literature,^22,36^ including classical methods using different catalysts, such as hydrogen chloride,^37^ sodium hydride,^28^ polyphosphoric acid PPA,^38–40^ phosphorus pentoxide methanesulfonic acid solution or Eaton's reagent,^41,42^ TiCl_4_,^43^ AgNO_3_,^44^ and Pd/C.^45^ Microwave irradiation was also used in the synthesis of various 4-hydroxy-2-quinolones from the condensation of anilines and other reagents comprising diethylmalonate,^46^ malonic acid,^47^ and activated arylmalonate.^48^

In view of the environmental concerns related to practising traditional chemical methods that involve the use of dangerous chemicals, finding a way that will lead to an applied chemistry that is green, ecofriendly, respectful of human health, and, simultaneously, more productive and low-cost is an essential requirement from chemists and scientists, especially in terms of searching for interesting new potentially active compounds.

In this context, our interest focused on the combination of the microwave method and the use of the heterogeneous catalyst BiCl_3_ to realize a green high-speed synthesis of modified analogues of 4-hydroxy-2-quinolones starting from simple, available, and easily accessible reagents, β-enaminones and diethylmalonate, resulting in a series of molecules: 4-hydroxydihydroquinoline-2,5-diones.

## Results and discussion

### Synthesis

In a continuation of our investigation of the use of microwave irradiation in synthesizing heterocyclic-based derivatives,^55^ as well as the use of β-enaminones as reactive synthetic intermediates leading to interesting compounds,^56^ we have developed a new, rapid, and environmentally friendly method for synthesizing hydroxyquinolone analogues. This method involves the condensation of β-enaminones with diethyl malonate CH_2_(CO_2_Et)_2_, catalyzed by BiCl_3_ under microwave irradiation in the presence of EtOH.

**Scheme 2:**
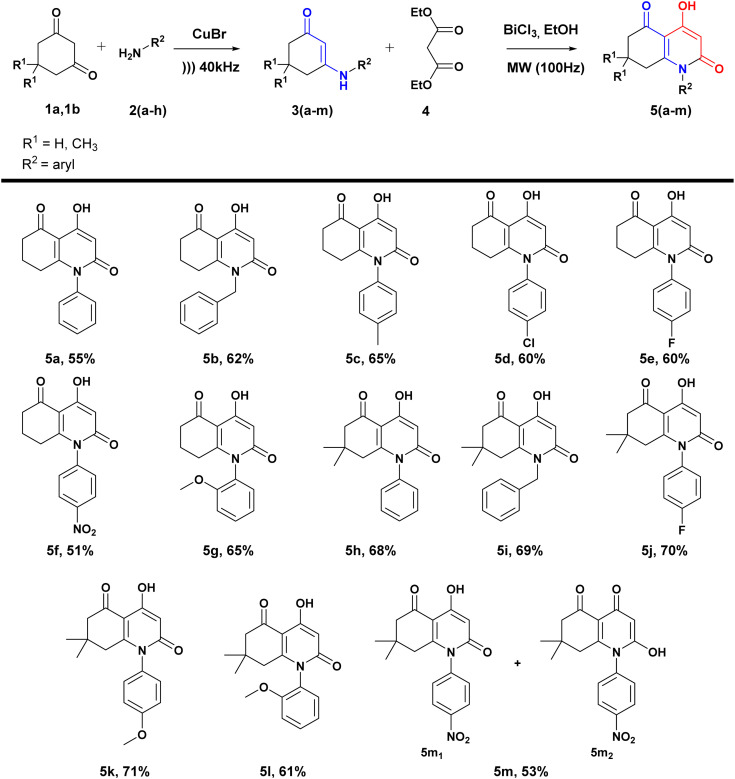
Synthetic green route leading to 4-hydroxyquinolin-2-one analogues.

The general synthetic route for these analogues is outlined in Scheme 2. The synthesis of the desired compounds occurs in two steps: first, β-enaminones are obtained using the method previously described by our group,^54^ including the condensation of dimedone or cyclohexanedione with primary aromatic amines under ultrasound irradiation catalyzed by CuBr.

**Scheme 3:**
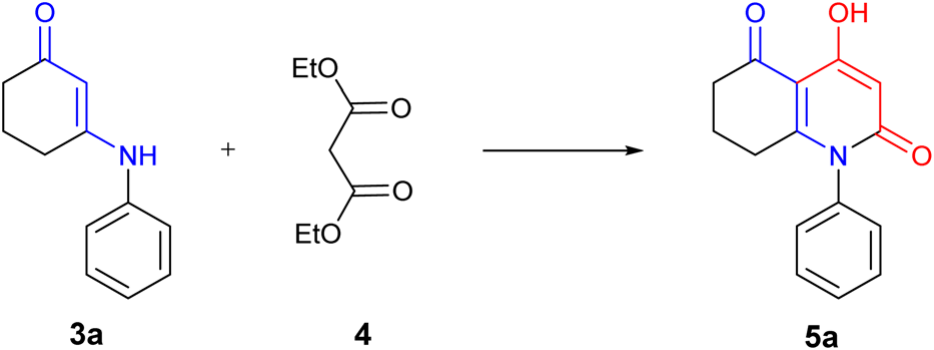
Model reaction for the synthesis of 4-hydroxy-2-quinolone analogue.

Then, β-enaminone (3a) was selected as a model substrate (Scheme 3) and was reacted with diethylmalonate under different reaction conditions in which we used both classical and green chemistry in order to find the optimal synthetic method (Table 1). Our first attempt was to perform the reaction at room temperature (Table 1, entry 1). After 48 hours, no product was observed. We increased the temperature by using reflux conditions; a small amount of the desired compound was obtained within a period of 48 hours (Table 1, entry 2). Due to the fact that reflux gave the desired product 5a in low yield within a long period of reaction time, the use of microwave irradiation as an alternative method of heating was worth trying. Indeed, the reaction occurred more rapidly with a significant increase in yield (Table 1, entry 3).

**Table 1:** Optimization of reaction conditions

Entry	Method	Solvent	Time	Yield (%)
1	r.t.	EtOH	48 h	No reaction
2	Reflux	EtOH	48 h	6
3	MW	Solvent-free	12 min	20

Regardless of obtaining better results when using microwave irradiation, a 20% yield is considered moderate; that is what prompted us to try several catalysts (Table 2) in order to improve the reaction conditions.

**Table 2:** Optimization of reaction time and catalyst under MW irradiation

Entry	Catalyst	Time (min)	Yield (%)
1	BiCl_3_ (20%)	8	48
2	Zn[OOCCH_3_]_2_	8	35
3	SiO_2_	15	29
4	K-10	16	24
5	ZnCl_2_	9	40
6	CsI	10	35
7	CuBr	10	38
8	AgNO_3_	11	40

Among the catalysts tried, silica gel (Table 2, entry 3) and montmorillonite (Table 2, entry 4) engendered a minor improvement in yields by 9 and 4%, respectively, compared to the reaction conduction without a catalyst. This slight effect remained insignificant as it was accompanied by an increase in reaction time. Unlike the above-mentioned catalysts, zinc acetate (Table 2, entry 2), cesium iodide (Table 2, entry 6), copper bromide (Table 2, entry 7), and silver nitrate (Table 2, entry 8) promoted the formation of final product in a better yield from 35 to 40% and a shorter time (8–11 min).

In the search for efficient catalysts, our attention was directed to BiCl_3_, a bismuth salt recognized for its availability and low toxicity, moreover, it is environmentally benign, criteria that are highly recommended from a green chemistry perspective.^57^ This Lewis acid catalyst and other bismuth-based catalysts have attracted wide interest and had extensive applications as activators in many chemical transformations, especially in the synthesis of heterocycles.^57,58^ These benefits encouraged us to explore the influence of bismuth(iii) chloride on reaction progress (Table 2, entry 1). The most promising results were perceived when using BiCl_3_, since we noticed a significant enhancement in the yield (48%) and a drop in reaction time (8 min).

Polar solvents play a key role in the generation of microwave heat that resides in the dipolar polarization mechanism; when subjected to the electric field produced by microwaves, molecules with substantial dipolar moments will tend to constantly rotate and consequently engender thermal energy.^59^ We have studied the effect of solvents on the reaction rate by testing different polar solvents starting from the safest and greenest one: H_2_O. The reaction did not occur as expected since the components of the reaction are not miscible with water. Other polar solvents were chosen for testing in our reaction, as shown in Table 3, including ethanol, methanol, and acetone. This choice was made based on the fact that these solvents are less toxic.

**Table 3:** Optimization of solvents using BiCl_3_ under MW

Entry	Solvent	Time (min)	Yield (%)
1	EtOH	5	55
2	MeOH	6	50
3	Acetone	8	46
4	Solvent-free	8	48

Unexpectedly, despite its polarity, acetone did not improve the yields nor the reaction time (Table 3, entry 3); methanol had a negligible impact on reaction time (Table 3, entry 2). In contrast, the yield was increased and the time was reduced when using ethanol (Table 3, entry 1).

Under these optimized conditions (microwave irradiation, catalyst (BiCl_3_ 20%), solvent (EtOH)), targeting potentially active compounds, several medicinally important substituents such as halogens (F, Cl), electron-donating groups (OCH_3_, CH_3_), and electron-withdrawing group (NO_2_) were introduced in different positions of the aromatic ring of β-enaminones. Both cyclohexanedione and dimedone were used as dicarbonylic precursors leading to β-enaminones (Scheme 2).

The obtained yields were significantly influenced by the nature of the substituents. Generally, dimedone-based β-enaminones led to higher yields, which can be explained by the presence of the two methyl groups. Additionally, electron-donating groups such as methyl and methoxy groups present in *para* and *ortho* positions (5c, 5g, 5k, 5l) improved yields by enhancing NH nucleophilicity. However, the presence of nitro groups in *para* positions (5f, 5m) reduced the NH reactivity and resulted in lower yields.

The main reason why the yields are moderate in most cases is the fact that the reaction is not complete; an amount of the β-enaminone used as a starting material remains in the reaction, and a prolongation of the reaction time to more than 15 minutes is not appropriate since it can cause degradation of the final product.

#### Spectral characterization

The structures of the synthesized compounds were confirmed using spectroscopic methods, including ^1^H, ^13^C NMR, and IR as well as elemental analysis. All spectra are available in the ESI file.†

The FT-IR spectrum showed all the bands of the characteristic functions present in the structures of the final products: namely, enolic OH function characterized by stretching at 3236–3449 cm^−1^, ketone and amide functions confirmed by C=O stretching bands at 1647–1738 cm^−1^, and C=C bonds absorbing in a range between 1511 and 1650 cm^−1^.

In the ^1^H-NMR spectrum, the formation of the enolic form was confirmed by a signal appearing as a singlet in deshielded chemical shifts (12.37–12.78 ppm) that correspond to enolic OH. Additionally, the proton attached to the C(α) (the carbon adjacent to C(OH)) appeared as a singlet at 5.61–5.87 ppm. The ^13^C NMR spectrum always exhibited signals in the range 95.58–98.16 ppm that indicates C(α).

Carbonyl groups signals of ketone and amide functions appeared at 201.27–202.60 ppm and 162.36–164.41 ppm, respectively, while the C–OH carbon signal appeared at 166.71–168.20 ppm.

**Fig. 1:**
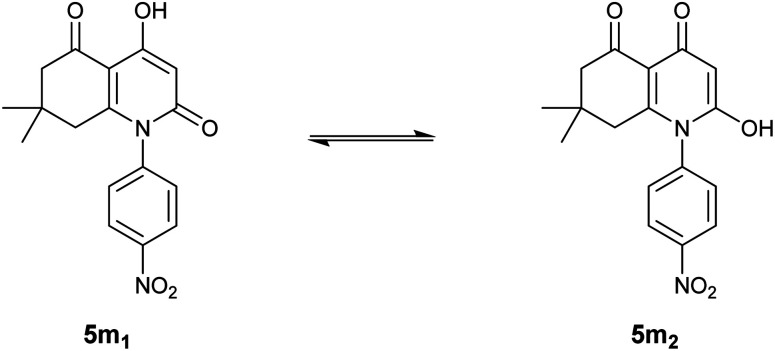
Obtained tautomeric forms for compound 5m.

Unlike the other compounds, we obtained *para*-nitrosubstituted derivative 5m as a mixture of two tautomers, as presented in Fig. 1, which indicates an equilibrium between two possible enolic forms: 4-hydroxyhydroquinoline-2,5-dione 5m_1_ and 2-hydroxyhydroquinoline-4,5-dione 5m_2_.

The presence of the two forms was concluded based on a general observation of the ^1^H-NMR spectrum that exhibited all the expected signals; moreover, identical signals were also observed in the spectrum in slightly different shifts and in lower intensities.

The tautomeric ratio between the two enolic forms was estimated by a simple analysis of integrals in the ^1^H-NMR spectrum of compound 5m. The results indicate a ratio of 5 : 1 in which 4-hydroxyhydroquinoline-2,5-dione 5m_1_ is the major form with a percentage of nearly 83%.

^1^H-NMR results for the 4-hydroxyhydroquinoline-2,5-dione 5m1 form showed two singlets at 5.87 and 12.36 ppm that correspond to enolic OH in position 4 and the proton attached to C(α), respectively. These findings are in perfect agreement with the NMR results for the rest of the synthesized compounds.

However, the enolic proton in the minor form, 2-hydroxyhydroquinoline-4,5-dione 5m_2_, appeared in more deshielded chemical displacement (13.99 ppm) which can be related to the negative mesomeric electron delocalization engendered by the electron-withdrawing nitro group present in the *para* position of the aromatic ring.

### Mechanistic proposal

**Scheme 4:**
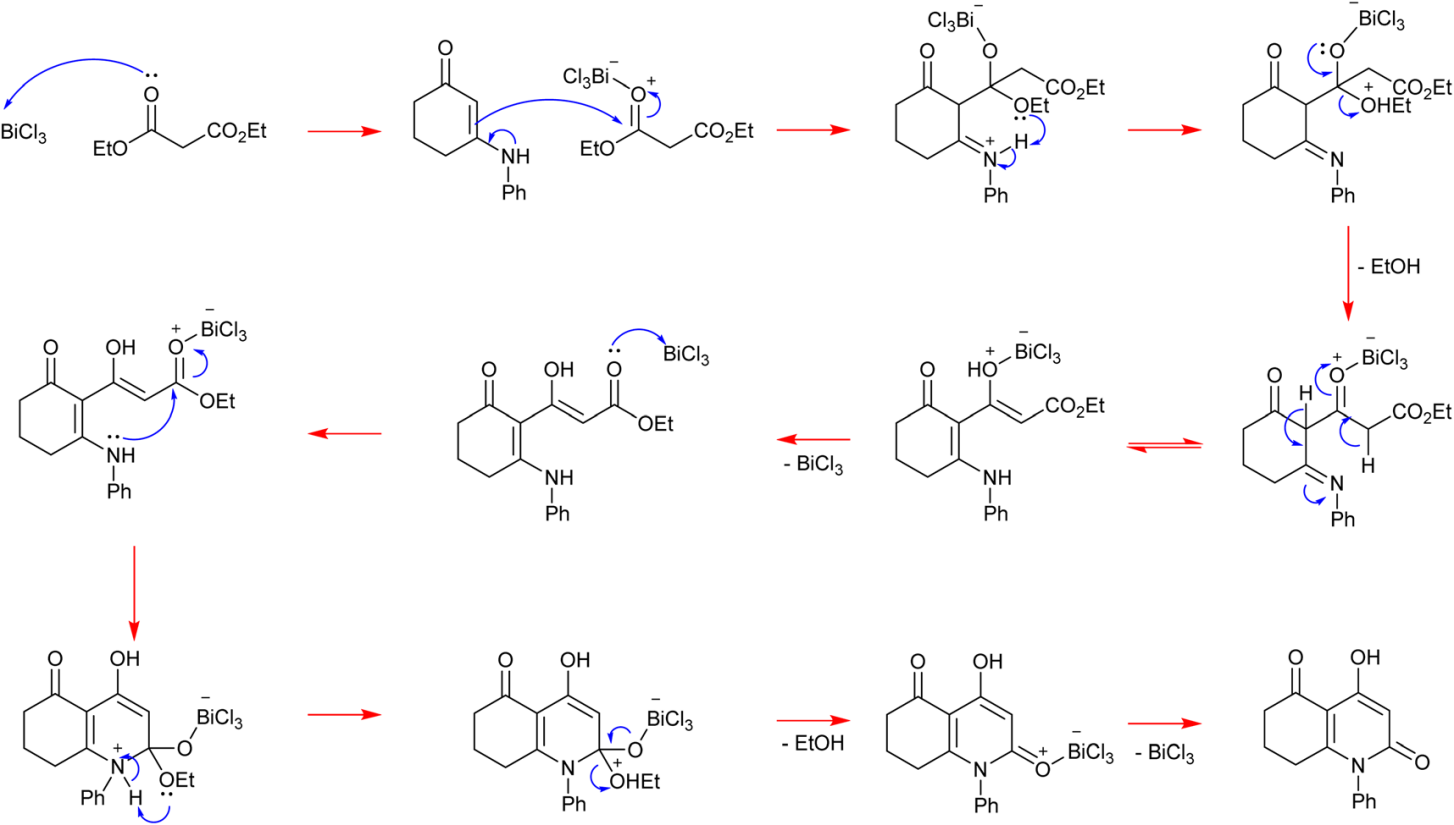
Mechanistic proposal for the BiCl_3_-catalyzed synthesis of 4-hydroxy-2-quinolone analogues.

Initially, the Lewis acid catalyst BiCl_3_ activates the carbonyl of the ester function in diethylmalonate, contributing to enhancing its electrophilicity. Then, the β-enaminone that contains two active sites performs a nucleophilic attack with its double bond activated by delocalization of electrons on the azote. This step is followed by the liberation of one ethanol molecule. After recovery of the catalyst we obtain an intermediate containing an ester function. This latter is activated by BiCl_3_ as well giving an electrophilic site that is attacked by the secondary amine of the β-enaminone, leading to the formation of a heterocyclic compound. Finally, a second molecule of ethanol is released and the catalyst is recovered, affording the heterocyclic final product (Scheme 4).

### Crystal characterization

A suitable crystal of compound 5i was subjected to a complete structural elucidation using single crystal X-ray diffraction. The structural resolution showed that the asymmetric unit consists of 8-hydroxy-3,3-dimethyl-5-(phenylamino)-3,4-dihydronaphthalene-1,6(2*H*,5*H*)-dione 5i which crystallizes in the triclinic crystal system with *P*1̄ space group (Table 5).

**Fig. 2:**
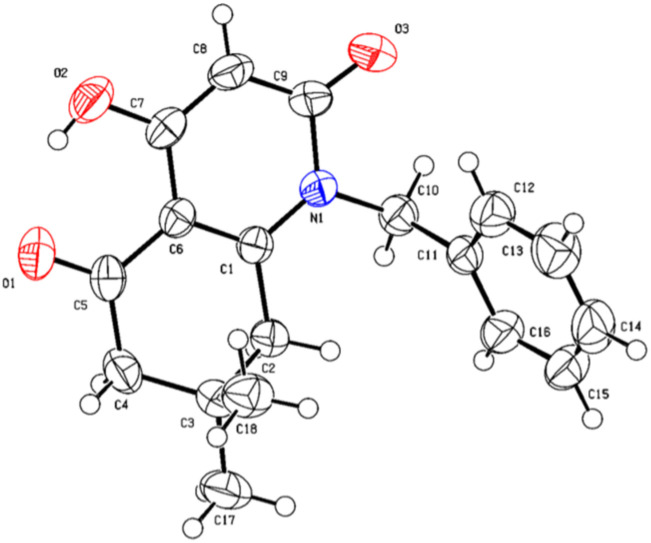
ORTEP diagram of compound 5i.

The ORTEP diagram is represented in Fig. 2. It is worth noting that the reaction of β-enaminone and diethyl malonate produced the enolic tautomer instead of the dicarbonylic one. The presence of the enol group allowed the formation of an intramolecular hydrogen bond O2–H2⋯O1 between the enolic proton and the carbonyl present in the substituted cyclohexenone ring with a length of 1.818 Å; this interaction gave a pseudocycle with S(6) graph-set motif.

**Fig. 3:**
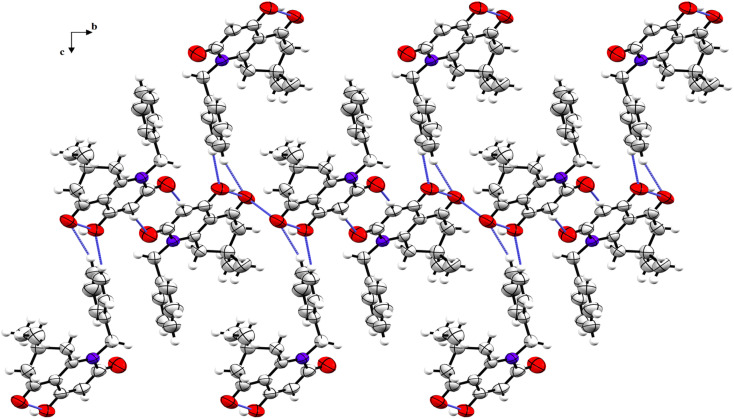
Crystal packing diagram of compound 5i viewed along the a-axis (H-bonds and short contacts are represented as blue dashed sticks).

The crystal structure is supported by intermolecular interactions of C–H⋯O type (Table 4) with lengths ranging between 2.424 and 2.695 Å forming three graph sets: two infinite chains and a ring with *R*_2_^2^(8) graph-set motif. An additional intermolecular interaction is perceived between the two identical oxygen atoms O1⋯O1 with a length equal to 3.008 Å. These interactions reinforce the cohesion of the crystal structure and keep the components linked together. A crystal packing diagram is represented to explore the repartition of the structural components in the crystal (Fig. 3). A hydrophobic interaction is also present in the structure and consists of π–π stacking between the benzylic aromatic rings.

**Table 4:** Distances (Å) and angles (°) of hydrogen bonds for compound 5i

D–H⋯A	*d*(D–H)	*d*(H⋯A)	*d*(D–A)	D–H–A	Symmetry
O2–H2⋯O1	0.820	1.818	2.553(2)	148.46	*x*, *y*, *z*
C14–H14⋯O1	0.930	2.593	3.495(2)	163.7	*x*, *y*, *z*, *x*, *y*, −1 + *z*
C15–H15⋯O2	0.930	2.695	3.595(2)	163.0	*x*, *y*, *z*, −1 + *x*, *y*,−1 + *z*
C8–H8⋯O3	0.930	2.424	3.344(2)	169.94	*x*, *y*, *z*, 2 − *x*, 1 − *y*, 2 − *z*

## Experimental

### Chemicals and methods

All chemicals and solvents were purchased from Sigma-Aldrich and Thermo-Fisher Scientific and were used as received without any further purification. All reactions were monitored by TLC on silica Merck 60 F_254_ percolated aluminium plates and were developed by spraying with ninhydrin solution (10% in EtOH). Proton nuclear magnetic resonance (^1^H-NMR) spectra were recorded on a Bruker spectrometer at 400 MHz. Chemical shifts are reported in *δ* units (ppm) with TMS as reference (*δ* 0.00). All coupling constants (*J*) are reported in Hertz. Multiplicity is indicated by one or more of the following: s (singlet), d (doublet), t (triplet), p (pentet), m (multiplet), dd (doublet of doublets), td (triplet of doublets), ddd (doublet of doublets of doublets). Carbon nuclear magnetic resonance (^13^C-NMR) spectra were recorded on a Bruker spectrometer at 100 MHz. Chemical shifts are reported in *δ* units (ppm) relative to CDCl_3_ or DMSO (*δ* 77.0 and 39.0–40.0). Infrared spectra were recorded on a PerkinElmer 600 spectrometer. The purity of the final compounds was determined by HPLC-MS analyses which were performed on a Shimadzu Prominence LC analytical system consisting of a Shimadzu LC-20AD binary HPLC pump, a Shimadzu CTO-10AS column oven, a Shimadzu SIL-20ACHT cooling autosampler, a Shimadzu CBM-20A system controller, a Shimadzu SPD-20MA diode array detector; and an LC-MS-2020 mass detector with single quadrupole equipped with electrospray ionization (all Shimadzu, Kyoto, Japan). The quantification was performed on a monolithic Chromolith RP-C18 column (2.1 mm × 50 mm, 1.8 μm particle size) with a gradient mobile phase of H_2_O/CH_3_CN (70 : 30, v/v) with 0.1% of formic acid to H_2_O/CH_3_CN (10 : 90, v/v) with 0.1% of formic acid at a flow rate of 0.5 mL min^−1^, with UV monitoring at a wavelength of 254 nm with a run time of 30 min. Microanalysis spectra were performed by an elemental analyser (Euro E.A. 3000-V3.0-single-2007), and the determined values were within the acceptable limits of the calculated values. Melting points were recorded on a Büchi B-545 apparatus in open capillary tubes.

Microwave-assisted reactions were carried out using a Biotage Initiator Microwave Synthesizer 2.0 with a nominal power of 400 W. The reactions were carried out in a reactor to microwave (volume: 10 mL) under pressure.

### Crystallography

Crystallographic data for the studied compound 8-hydroxy-3,3-dimethyl-5-(phenylamino)-3,4-dihydronaphthalene-1,6(2*H*,5*H*)-dione 5i was collected on a SuperNova, Dual, Cu at home/near, AtlasS2 four-circle diffractometer equipped with an AtlasS2 CCD detector using Mo K\α (micro-focus sealed tube) radiation (*λ* = 0.71073 Å). The crystal was kept at a temperature of 295 K during data collection.

The crystallographic data and experimental details for structural analysis are summarized in Table 5. The reported structure was solved with the SHELXT-2014/5 (ref. 49) solution program by Intrinsic Phasing with Olex2 (ref. 50) as the graphical interface. The model was refined with SHELXL-2018/3 (ref. 51) using full matrix least-squares minimization on *F*^2^. All absorption corrections were performed with CrysAlisPro 1.171.42.51a^52^ using spherical harmonics implemented in the SCALE3 ABSPACK scaling algorithm. Crystal structure visualization and construction of crystal packing diagrams were performed using Mercury 3.8 software.^53^

**Table 5:** Crystallographic data and refinement parameters of compound 5i

Formula	C_18_H_19_NO_3_	Absorption coefficient (mm^−1^)	0.089
Formula weight (g mol^−1^)	297.34	F(000)	316.0
Crystal habit, colour	Prism, colorless	Crystal size (mm)	0.32 × 0.14 × 0.08
Crystal system	Triclinic	*θ* range for data collection (°)	2.360–33.343
Space group	*P*1̄	Reflections collected	19 501
*a* (Å)	6.4370(3)	Independent reflections	5272
*b* (Å)	10.9513(4)	*R*_int_	0.0259
*c* (Å)	11.3400(6)	Number of parameters	202
*α* (°)	102.588(4)	Goodness-of-fit on *F*^2^	1.043
*β* (°)	102.906(4)	Final *R* indices [I ≥ 2*σ*(*I*)]	*R*_1_ = 0.0552, *wR*_2_ = 0.1414
*γ* (°)	91.799(3)	*R* Indices [all data]	*R*_1_ = 0.0799, *wR*_2_ = 0.1579
Volume (Å^3^)	757.77(6)	Largest difference peak and hole (Å^−3^)	0.24, −0.17
*Z*, *Z*′	2, 0	CCDC deposition no.	CCDC 2256921
Density (calculated) (g cm^−3^)	1.303		

### General procedure for the synthesis of β-enaminone derivatives

The synthesis of β-enaminones was done according to the method described by Redjemia *et al.*^54^

In a microwave reactor (volume: 20 mL) was taken a mixture of dimedone or cyclohexanedione (1 mmol), an amine (1 mmol), and CuBr (0.05 mmol). The reaction mixture was subjected to ultrasound with a frequency of 40 kHz for an appropriate time at room temperature. The progress of the reaction was monitored by TLC. After completion of the reaction, EtOAc (5 mL) or DCM (5 mL) was added. The catalyst was recovered from the residue and the filtrate was concentrated. A (1/1) mixture of diethyl ether and *n*-hexane was added to the reaction mixture and the pure product was crystallized to 6 °C overnight. The product was finally filtered and dried. This procedure was followed for the preparation of all the β-enaminones used in the synthesis of 4-hydroxyquinolin-2-one analogues.

### General procedure for the synthesis of 4-hydroxy-2-quinolone derivatives

To a glass tube (diameter: 25 mm; thickness: 1 mm; volume: 20 mL) was introduced a 3 : 1 mixture of diethyl malonate and β-enaminone in 1 mL of ethanol as a solvent. Then, 0.2 mmol of BiCl_3_ was added to the reaction mixture. The reaction content was subjected to microwave irradiation for an appropriate time varying between 5 and 13 minutes. The progress of the reaction was monitored by TLC. After completion of the reaction, 5 mL of ethanol was added and the catalyst was recovered by filtration. The synthesized derivatives were purified through column chromatography eluted with a 1 : 1 mixture of ethyl acetate and petroleum ether. Pure layers were then concentrated under vacuum.

#### 4-Hydroxy-1-phenyl-7,8-dihydroquinoline-2,5(1*H*,6*H*)-dione (Scheme 2, entry 5a)

Colorful powder; 55% yield; mp = 162–164 °C; *R*_f_ = 0.35 (CH_3_CO_2_C_2_H_5_/petroleum ether, 60 : 40). IR (KBr, cm^−1^): 3261.97, 3063.51, 2943.69, 2890.57, 1710.86, 1592.00, 1574.67, 1530.95, 1492.38; ^1^H-NMR (400 MHz, DMSO-*d*_6_): *δ* = 1.91 (p, 2H, *J* = 6.4 Hz, CH_2_), 2.43 (t, 2H, *J* = 6.2 Hz, CH_2_–C), 2.54 (t, 2H, *J* = 5.6 Hz, 2H, CH_2_–CO), 5.63 (s, 1H, CH), 7.23–7.42 (m, 2H, Ar–H), 7.42–7.64 (m, 3H, Ar–H), 12.71 (s, 1H, OH); ^13^C NMR (101 MHz, DMSO-*d*_6_): *δ* = 19.98, 28.86, 35.82, 95.86 (CH), 104.70, 128.14, 128.93, 129.48, 137.35, 162.12, 162.62 (N–C=O), 167.24 (C–OH), 202.53 (C=O); MS: (*m*/*z*) = 256 (M + 1); anal. calc. for C_15_H_13_NO_3_ C, 70.58; H, 5.13; N, 5.49; found: C, 70.62; H, 5.10; N, 5.44.

#### 1-Benzyl-4-hydroxy-7,8-dihydroquinoline-2,5(1*H*,6*H*)-dione (Scheme 2, entry 5b)

Crystal; 62% yield; mp = 178–180 °C; *R*_f_ = 0.41 (CH_3_CO_2_C_2_H_5_/petroleum ether, 60 : 40). IR (KBr, cm^−1^): 3373.50, 1647.22, 1590.66, 1530.19, 1453.38, 1421.55; ^1^H-NMR (400 MHz, DMSO-*d*_6_): *δ* = 1.94 (p, 2H, *J* = 6.3 Hz, CH_2_), 2.54 (t, 2H, *J* = 6.0 Hz, CH_2_–C), 2.91 (t, 2H, *J* = 6.1 Hz, 2H, CH_2_–CO), 5.35 (s, 2H, N–CH_2_), 5.68 (s, 1H, CH), 7.12–7.19 (m, 2H, Ar–H), 7.24–7.40 (m, 3H, Ar–H), 12.78 (s, 1H, OH); ^13^C NMR (101 MHz, DMSO-*d*_6_): *δ* = 19.91, 27.09, 35.62, 45.90, 95.62 (CH), 105.05, 126.15, 127.26, 128.76, 136.17, 162.22, 162.62 (N–C=O), 167.03 (C–OH), 202.60 (C=O); anal. calc. for C_16_H_15_NO_3_ C, 71.36; H, 5.61; N, 5.20; found: C, 71.38; H, 5.63; N, 5.18.

#### 4-Hydroxy-1-(*p*-tolyl)-7,8-dihydroquinoline-2,5(1*H*,6*H*)-dione (Scheme 2, entry 5c)

Crystal; 65% Yield; mp = 222–224 °C; *R*_f_ = 0.45 (CH_3_CO_2_C_2_H_5_/petroleum ether, 60 : 40). IR (KBr, cm^−1^): 3391.87, 2957.15, 1655.76, 1607.02, 1511.88, 1441.96; ^1^H-NMR (400 MHz, CDCl_3_): *δ* = 1.97–2.03 (m, 2H, CH_2_), 2.42 (s, 3H, CH_3_), 2.47 (t, 2H, *J* = 6.2 Hz, CH_2_–C), 2.57 (t, 2H, *J* = 6.0 Hz, CH_2_–CO), 5.87 (s, 1H, CH), 7.05 (d, 2H, *J* = 8.2 Hz, Ar–H), 7.32 (d, 2H, *J* = 8.0 Hz, Ar–H), 12.43 (s, 1H, OH); ^13^C NMR (101 MHz, CDCl_3_): *δ* = 20.81, 21.36, 29.41, 36.59, 98.01 (CH), 105.98, 127.63, 130.82, 134.77, 139.70, 160.40, 164.05 (N–C=O), 167.78 (C–OH), 201.59 (C=O); MS: (*m*/*z*) = 270 (M + 1); anal. calc. for C_16_H_15_NO_3_ C, 71.36; H, 5.61; N, 5.20; C, 71.31; H, 5.64; N, 5.23.

#### 1-(4-Chlorophenyl)-4-hydroxy-7,8-dihydroquinoline-2,5(1*H*,6*H*)-dione (Scheme 2, entry 5d)

Crystal; 60% yield; mp = 240–242 °C; *R*_f_ = 0.62 (CH_3_CO_2_C_2_H_5_/petroleum ether, 60 : 40). IR (KBr, cm^−1^): 3258.20, 2924.99, 1673.74, 1532.55, 1489.79, 1403.58; ^1^H-NMR (400 MHz, DMSO-*d*_6_): *δ* = 1.92 (p, 2H, *J* = 6.3 Hz, CH_2_), 2.44 (t, 2H, *J* = 6.2 Hz, CH_2_–C), 2.54 (t, 2H, *J* = 6.0 Hz, CH_2_–CO), 5.64 (s, 1H, CH), 7.32–7.40 (m, 2H, Ar–H), 7.58–7.66 (m, 2H, Ar–H), 12.70 (s, 1H, OH); ^13^C NMR (101 MHz, DMSO-*d*_6_): *δ* = 19.96, 28.86, 35.82, 95.81 (CH), 104.82, 129.53, 130.21, 133.64, 136.18, 162.10, 162.52 (N–C=O), 167.33 (C–OH), 202.53 (C=O); MS: (*m*/*z*) = 290 (M + 1); anal. calc. for C_15_H_12_ClNO_3_ C, 62.19; H, 4.18; N, 4.83; found: C, 62.15; H, 4.14; N, 4.80.

#### 1-(4-Fluorophenyl)-4-hydroxy-7,8-dihydroquinoline-2,5(1*H*,6*H*)-dione (Scheme 2, entry 5e)

Crystal; 60% yield; mp = 226–228 °C; *R*_f_ = 0.49 (CH_3_CO_2_C_2_H_5_/petroleum ether, 60 : 40). IR (KBr, cm^−1^): 3398.70, 2921.08, 1728.10, 1661.78, 1605.05, 1584.15, 1559.12, 1401.03; ^1^H-NMR (400 MHz, CDCl_3_): *δ* = 1.97–2.08 (m, 2H, CH_2_), 2.48 (t, 2H, *J* = 6.2 Hz, CH_2_–C), 2.58 (t, 2H, *J* = 6.0 Hz, CH_2_–CO), 5.86 (s, 1H, CH), 7.13–7.19 (m, 2H, Ar–H), 7.19–7.25 (m, 2H, Ar–H), 12.43 (s, 1H, OH); ^13^C NMR (101 MHz, CDCl_3_): *δ* = 20.78, 29.45, 36.54, 98.02 (CH), 106.16, 117.17, 117.40, 129.82, 129.91, 133.19, 133.22, 160.13, 163.89 (N–C=O), 167.89 (C–OH), 201.58 (C=O); MS: (*m*/*z*) = 274 (M + 1); anal. calc. for C_15_H_12_FNO_3_ C, 65.93; H, 4.43; N, 5.13; found: C, 65.99; H, 4.47; N, 5.10.

#### 4-Hydroxy-1-(4-nitrophenyl)-7,8-dihydroquinoline-2,5(1*H*,6*H*)-dione (Scheme 2, entry 5f)

Crystal; 51% yield; mp = 120–122 °C; *R*_f_ = 0.5 (CH_3_CO_2_C_2_H_5_/petroleum ether, 60 : 40). IR (KBr, cm^−1^): 3351.01, 2924.80, 1668.38, 1644.20, 1525.04; ^1^H-NMR (400 MHz, CDCl_3_): *δ* = 2.03–2.09 (m, 2H, CH_2_), 2.47 (t, 2H, *J* = 6.2 Hz, CH_2_–C), 2.62 (t, 2H, *J* = 6.6 Hz, CH_2_–CO), 5.88 (s, 1H, CH), 7.42 (d, 2H, *J* = 8.4 Hz, Ar–H), 8.40 (d, 2H, *J* = 8.3 Hz, Ar–H), 12.44 (s, 1H, OH); ^13^C NMR (101 MHz, CDCl_3_): *δ* = 20.77, 29.43, 36.53, 98.16 (CH), 106.46, 125.47, 129.69, 143.41, 148.40, 160.21, 163.41 (N–C=O), 168.25 (C–OH), 201.46 (C=O); anal. calc. for C_15_H_12_N_2_O_5_ C, 60.00; H, 4.03; N, 9.33; found: C, 60.05; H, 4.08; N, 9.37.

#### 4-Hydroxy-1-(2-methoxyphenyl)-7,8-dihydroquinoline-2,5(1*H*,6*H*)-dione (Scheme 2, entry 5g)

Crystal; 65% Yield; mp = 171–173 °C; *R*_f_ = 0.29 (CH_3_CO_2_C_2_H_5_/petroleum ether, 60 : 40). IR (KBr, cm^−1^): 3401.19, 2952.72, 1682.96, 1650.30, 1528.11, 1503.17, 1410.66; ^1^H-NMR (400 MHz, DMSO-*d*_6_): *δ* = 1.66–2.05 (m, 2H, CH_2_), 2.22–2.35 (m, 2H, CH_2_–C), 2.4–2.68 (m, 2H, CH_2_–CO), 3.76 (s, 3H, CH_3_), 5.61 (s, 1H, CH), 7.10 (td, 1H, *J* = 1.2, 7.6 Hz, Ar–H), 7.23 (dd, 2H, *J* = 1.7, 7.7 Hz, Ar–H), 7.49 (ddd, 1H, *J* = 1.7, 7.4, 8.3 Hz, Ar–H), 12.68 (s, 1H, OH); ^13^C NMR (101 MHz, DMSO-*d*_6_): *δ* = 19.97, 27.77, 35.82, 55.86, 95.83 (CH), 104.65, 112.57, 120.93, 125.47, 129.27, 130.72, 154.09, 162.10, 162.36 (N–C=O), 167.19 (C–OH), 202.42 (C=O); MS: (*m*/*z*) = 286 (M + 1); anal. calc. for C_16_H_15_NO_4_ C, 67.36; H, 5.30; N, 4.91; found: C, 67.31; H, 5.25; N, 4.87.

#### 4-Hydroxy-7,7-dimethyl-1-phenyl-7,8-dihydroquinoline-2,5(1*H*,6*H*)-dione (Scheme 2, entry 5h)

Crystal; 68% yield; mp = 210–212 °C; *R*_f_ = 0.47 (CH_3_CO_2_C_2_H_5_/petroleum ether, 60 : 40). IR (KBr, cm^−1^): 3429.88, 2963.91, 1676.72, 1592.40, 1536.24, 1455.51, 1405.54; ^1^H-NMR (400 MHz, CDCl_3_): *δ* = 1.03 (s, 6H, 2CH_3_), 2.32 (s, 2H, CH_2_–C), 2.44 (s, 2H, CH_2_–CO), 5.87 (s, 1H, CH), 7.13 (d, 2H, *J* = 7.2 Hz, Ar–H), 7.11–7.18 (m, 2H, Ar–H), 7.46–7.59 (m, 3H, Ar–H), 12.39 (s, 1H, OH); ^13^C NMR (101 MHz, CDCl_3_): *δ* = 28.14, 32.63, 42.81, 50.16, 97.91 (CH), 104.99, 128.03, 129.58, 130.26, 137.45, 158.73, 164.13 (N–C=O), 167.62 (C–OH), 201.28 (C=O); MS: (*m*/*z*) = 284 (M + 1); anal. calc. for C_17_H_17_NO_3_ C, 72.07; H, 6.05; N, 4.94; found: C, 72.10; H, 6.08; N, 4.99.

#### 1-Benzyl-4-hydroxy-7,7-dimethyl-7,8-dihydroquinoline-2,5(1*H*,6*H*)-dione (Scheme 2, entry 5i)

Crystal; 69% yield; mp = 168–170 °C; *R*_f_ = 0.64 (CH_3_CO_2_C_2_H_5_/petroleum ether, 60 : 40). IR (KBr, cm^−1^): 3350.20, 3031.30, 2954.97, 1659.17, 1632.99, 1586.34, 1443.85; ^1^H-NMR (400 MHz, DMSO-*d*_6_): *δ* = 0.92 (s, 6H, 2CH_3_), 2.48 (s, 2H, CH_2_–C), 2.84 (s, 2H, CH_2_–CO), 5.37 (s, 2H, N–CH_2_), 5.68 (s, 1H, CH), 7.13 (d, 2H, *J* = 7.2 Hz, Ar–H), 7.25–7.30 (m, 1H, Ar–H), 7.36 (dd, 2H, *J* = 4.6, 10.1 Hz, Ar–H), 12.65 (s, 1H, OH); ^13^C NMR (101 MHz, DMSO-*d*_6_): *δ* = 27.33, 31.85, 45.70, 48.78, 95.58 (CH), 104.08, 125.91, 127.21, 128.69, 136.27, 160.56, 162.70 (N–C=O), 166.71 (C–OH), 202.00 (C=O); MS: (*m*/*z*) = 298 (M + 1); anal. calc. for C_18_H_19_NO_3_ C, 72.71; H, 6.44; N, 4.71; found: C, 72.74; H, 6.40; N, 4.65.

#### 1-(4-Fluorophenyl)-4-hydroxy-7,7-dimethyl-7,8-dihydroquinoline-2,5(1*H*,6*H*)-dione (Scheme 2, entry 5j)

Yellow powder; 70% yield; mp = 193–195 °C; *R*_f_ = 0.66 (CH_3_CO_2_C_2_H_5_/petroleum ether, 60 : 40). IR (KBr, cm^−1^): 3373.70, 2956.29, 1665.73, 1625.38, 1526.83, 1508.41; ^1^H-NMR (400 MHz, CDCl_3_): *δ* = 1.04 (s, 6H, 2CH_3_), 2.31 (s, 2H, CH_2_–C), 2.44 (s, 2H, CH_2_–CO), 5.85 (s, 1H, CH), 7.11–7.14 (m, 2H, Ar–H), 7.20–7.25 (m, 2H, Ar–H), 12.37 (s, 1H, OH); ^13^C NMR (101 MHz, CDCl_3_): *δ* = 28.15, 32.63, 42.89, 50.07, 97.84 (CH), 105.11, 117.27, 117.50, 129.92, 133.20, 158.68, 161.63, 164.11 (N–C=O), 167.67 (C–OH), 201.27 (C=O); MS: (*m*/*z*) = 302 (M + 1); anal. calc. for C_17_H_16_FNO_3_ C, 67.76; H, 5.35; N, 4.65; found: C, 67.71; H, 5.34; N, 4.61.

#### 4-Hydroxy-1-(4-methoxyphenyl)-7,7-dimethyl-7,8-dihydroquinoline-2,5(1*H*,6*H*)-dione (Scheme 2, entry 5k)

Crystal; 71% yield; mp = 186–188 °C; *R*_f_ = 0.44 (CH_3_CO_2_C_2_H_5_/petroleum ether, 60 : 40). IR (KBr, cm^−1^): 3431.97, 2960.15, 1676.28, 1609.50, 1534.74, 1510.66, 1457.69, 1403.88; ^1^H-NMR (400 MHz, CDCl_3_): *δ* = 1.03 (s, 6H, 2CH_3_), 2.34 (s, 2H, CH_2_–C), 2.43 (s, 2H, CH_2_–CO), 3.86 (s, 3H, CH_3_), 5.86 (s, 1H, CH), 7.04 (s, 4H, Ar–H), 12.37 (s, 1H, OH); ^13^C NMR (101 MHz, CDCl_3_): *δ* = 28.15, 32.58, 42.87, 50.13, 55.68, 97.79 (CH), 104.98, 115.48, 128.98, 129.85, 159.26, 160.16, 164.41 (N–C=O), 167.55 (C–OH), 201.28 (C=O); MS: (*m*/*z*) = 314 (M + 1); anal. calc. for C_18_H_19_NO_4_ C, 69.00; H, 6.11; N, 4.47; found: C, 69.04; H, 6.13; N, 4.49.

#### 4-Hydroxy-1-(2-methoxyphenyl)-7,7-dimethyl-7,8-dihydroquinoline-2,5(1*H*,6*H*)-dione (Scheme 2, entry 5l)

Yellow powder; 61% yield; mp = 176–178 °C; *R*_f_ = 0.52 (CH_3_CO_2_C_2_H_5_/petroleum ether, 60 : 40). IR (KBr, cm^−1^): 3236.20, 2928.01, 1738.11 1668.33, 1532.10, 1496.44, 1455.64; ^1^H-NMR (400 MHz, CDCl_3_): *δ* = 1.03 (d, 6H, *J* = 2.2 Hz, 2CH_3_), 2.23 (d, 1H, *J* = 17.6, CH–C), 2.36 (d, 2H, *J* = 17.6, CH–C), 2.43 (s, 2H, CH_2_–CO), 3.80 (s, 3H, CH_3_), 5.86 (s, 1H, CH), 7.04–7.17 (m, 3H, Ar–H), 7.47 (ddd, 1H, *J* = 3.5, 5.8, 8.3 Hz, Ar–H), 12.38 (s, 1H, OH); ^13^C NMR (101 MHz, CDCl_3_): *δ* = 27.74, 28.61, 32.47, 41.82, 50.19, 55.99, 97.76 (CH), 104.94, 112.55, 121.67, 125.90, 129.31, 131.17, 154.41, 159.75, 163.85 (N–C=O), 167.63 (C–OH), 201.31 (C=O); MS: (*m*/*z*) = 314 (M + 1); anal. calc. for C_18_H_19_NO_4_ C, 69.00; H, 6.11; N, 4.47; found: C, 69.05; H, 6.17; N, 4.50.

#### 4-Hydroxy-7,7-dimethyl-1-(4-nitrophenyl)-7,8-dihydroquinoline-2,5(1*H*,6*H*)-dione (5m1) + 2-hydroxy-7,7-dimethyl-1-(4-nitrophenyl)-7,8-dihydroquinoline-4,5(1*H*,6*H*)-dione (5m2) (Scheme 2, entry 5m)

Oil; 53% Yield; *R*_f_ = 0.6 (CH_3_CO_2_C_2_H_5_/petroleum ether, 60 : 40). IR (KBr, cm^−1^): 3449.20, 3380.30, 2962.36, 1737.93, 1663.83, 1598.51, 1563.30, 1529.11, 1510.45; ^1^H-NMR (400 MHz, CDCl_3_): (5m1:5m2) (5 : 1); 5m1*δ* = 1.06 (s, 6H, 2CH_3_), 2.30 (s, 2H, CH_2_–C), 2.47 (s, 2H, CH_2_–CO), 5.88 (s, 1H, CH), 7.30–7.43 (m, 2H, Ar–H), 8.38–8.46 (m, 2H, Ar–H), 12.37 (s, 1H, OH); 5m2*δ* = 1.09 (s, 6H, 2CH_3_), 2.33 (s, 2H, CH_2_–C), 2.67 (s, 2H, CH_2_–CO), 5.52 (s, 1H, CH), 6.96–7.04 (m, 2H, Ar–H), 8.26–8.32 (m, 2H, Ar–H), 13.59 (s, 1H, OH); ^13^C NMR (101 MHz, CDCl_3_): *δ* = 28.01, 28.16, 31.72, 32.81, 41.41, 42.88, 50.07, 62.43, 90.50 (CH), 98.02 (CH), 104.83, 105.37, 119.59, 125.17, 125.59, 129.70, 142.95, 143.39, 148.40, 151.77, 157.53, 163.58 (N–C=O), 167.95 (C–OH), 170.05, 170.14, 170.41 (N–C=O), 171.19 (C–OH), 201.20 (C=O), 204.49 (C=O); anal. calc. for C_17_H_16_N_2_O_5_ C, 62.19; H, 4.91; N, 8.53; found: C, 62.23; H, 4.97; N, 8.58.

## Conclusions

We have developed a new synthetic method leading to nitrogen-based heterocycles structurally analogous to 4-hydroxy-2-quinolones. We adopted a simple, benign synthesis which is respectful to the requirements of green chemistry by using microwaves as an effective source of heat and BiCl_3_ as a non-toxic, safe, and accessible Lewis acid catalyst that activated the transformation between β-enaminones and diethyl malonate. Another positive aspect of our synthesis is the use of available and easily prepared starting materials. We obtained the desired compounds in good yields within a short time, which was ensured by the successful combination of microwave-assisted synthesis and heterogeneous catalysis. A spectral characterization of the structures was carried out using IR, ^1^H, and ^13^C spectroscopy as well as elemental analysis. The structure of compound 5i was deduced *via* the single crystal X-ray diffraction method that confirmed the obtention of the enolic tautomer.

## Author contributions

Yousra Ouafa Bouone: investigation, writing – original draft, methodology. Abdeslem Bouzina: conceptualization, validation, supervision, writing – review & editing. Rayene Sayad: data curation, formal analysis. Abdelhak Djemel, Farouk Benaceur, Abdelhalim Zoukel and Malika Ibrahim-Ouali: resources. Nour-Eddine Aouf and Fouzia Bouchareb: review & editing.

## Conflicts of interest

The authors declare that they have no competing interests.

## Supplementary Material

RA-013-D3RA05289C-s001

RA-013-D3RA05289C-s002
