# Supplementary material for: BiCl3-catalyzed green synthesis of 4-hydroxy-2-quinolone analogues under microwave irradiation
Source: RSC Adv. 2023 Sep 21;13(40):28030–41. doi: 10.1039/d3ra05289c (PMC10517106; doi:10.1039/d3ra05289c)
Supplement: RA-013-D3RA05289C-s001 [file RA-013-D3RA05289C-s001.pdf]

Electronic Supplementary Material (ESI) for RSC Advances.
This journal is © The Royal Society of Chemistry 2023

$BiCl_3$-catalyzed green synthesis of 4-hydroxy-2-quinolone analogues under microwave irradiation

Yousra Ouafa Bouone[a,b], Abdeslem Bouzina[a*], Rayene Sayad[a], Abdelhak Djemel[c], Farouk Benaceur[c], Abdelhalim Zoukel[d], Malika Ibrahim-Ouali[e], Nour-Eddine Aouf[a], Fouzia Bouchareb[b,f]

[a]Laboratory of Applied Organic Chemistry, Bioorganic Chemistry Group, Department of Chemistry, Sciences Faculty, Badji-Mokhtar - Annaba University, Box 12, 23000 Annaba, Algeria.

[b]Laboratory of Applied Organic Chemistry, Synthesis of Biomolecules and Molecular Modelling Group, Department of Chemistry, Sciences Faculty, Badji-Mokhtar - Annaba University, Box 12, 23000 Annaba, Algeria.

[c]Research Unit in Medicinal Plants, URPM, 3000 Laghouat, attached to Research Center of Biotechnology, CRBt, 25000 Constantine, Algeria.

[d]Technical platform of physico-chemical analysis (PTAPC-Laghout-CRAPC), University of Laghouat, Laghouat 03000, Algeria.

[e]Aix Marseille Univ, CNRS, Centrale Marseille, iSm2, F-13397 Marseille, France.

[f]Faculty of Sciences and Technology, Department of Chemistry, Chadli Bendjedid - EL Tarf University, P.O.Box: 73, El Tarf 36000 Algeria.

*Corresponding Author. Email: abdeslem.bouzina@univ-annaba.dz; bouzinaabdeslem@yahoo.fr

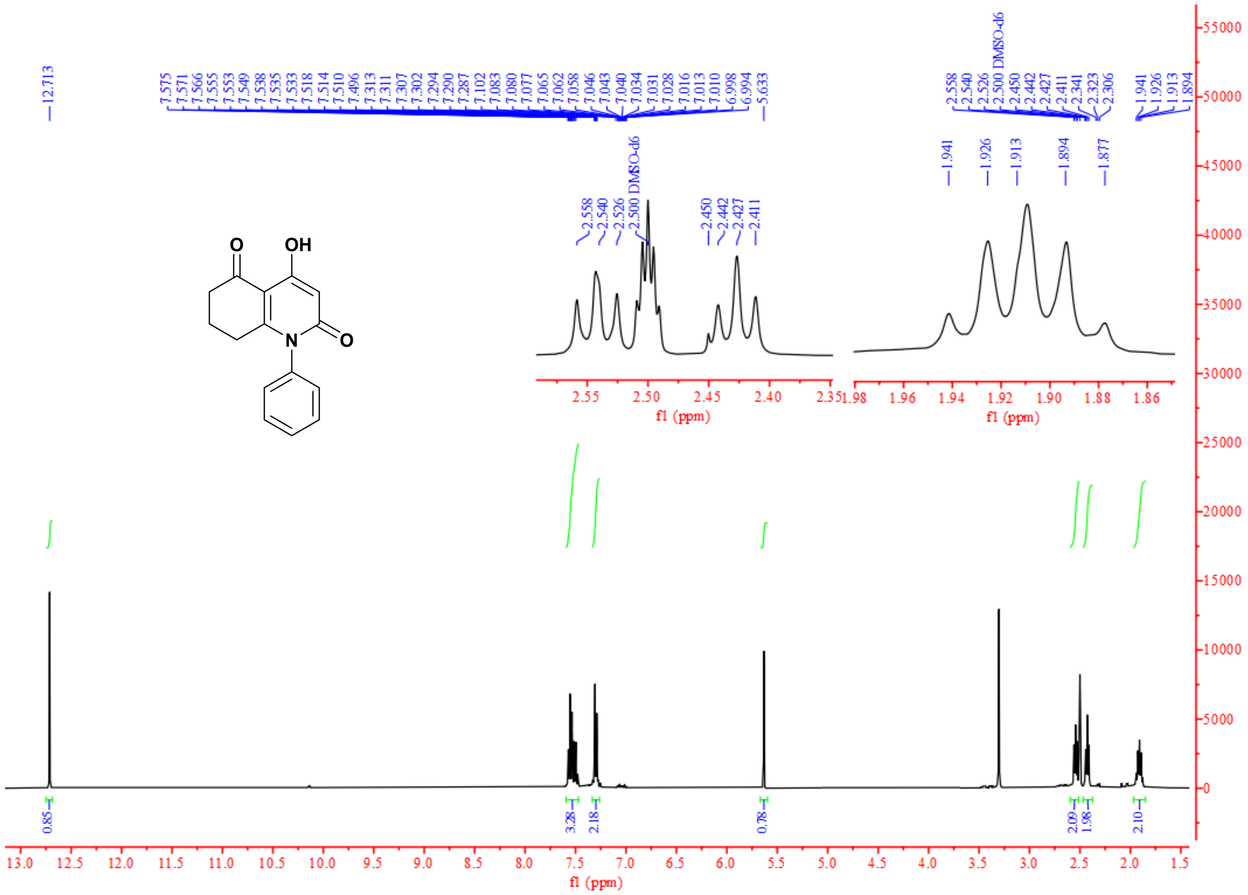

NMR ^{1}H spectrum (400 MHz, DMSO-d_6): 4-hydroxy-1-phenyl-7,8-dihydroquinoline-2,5(1H,6H)-dione

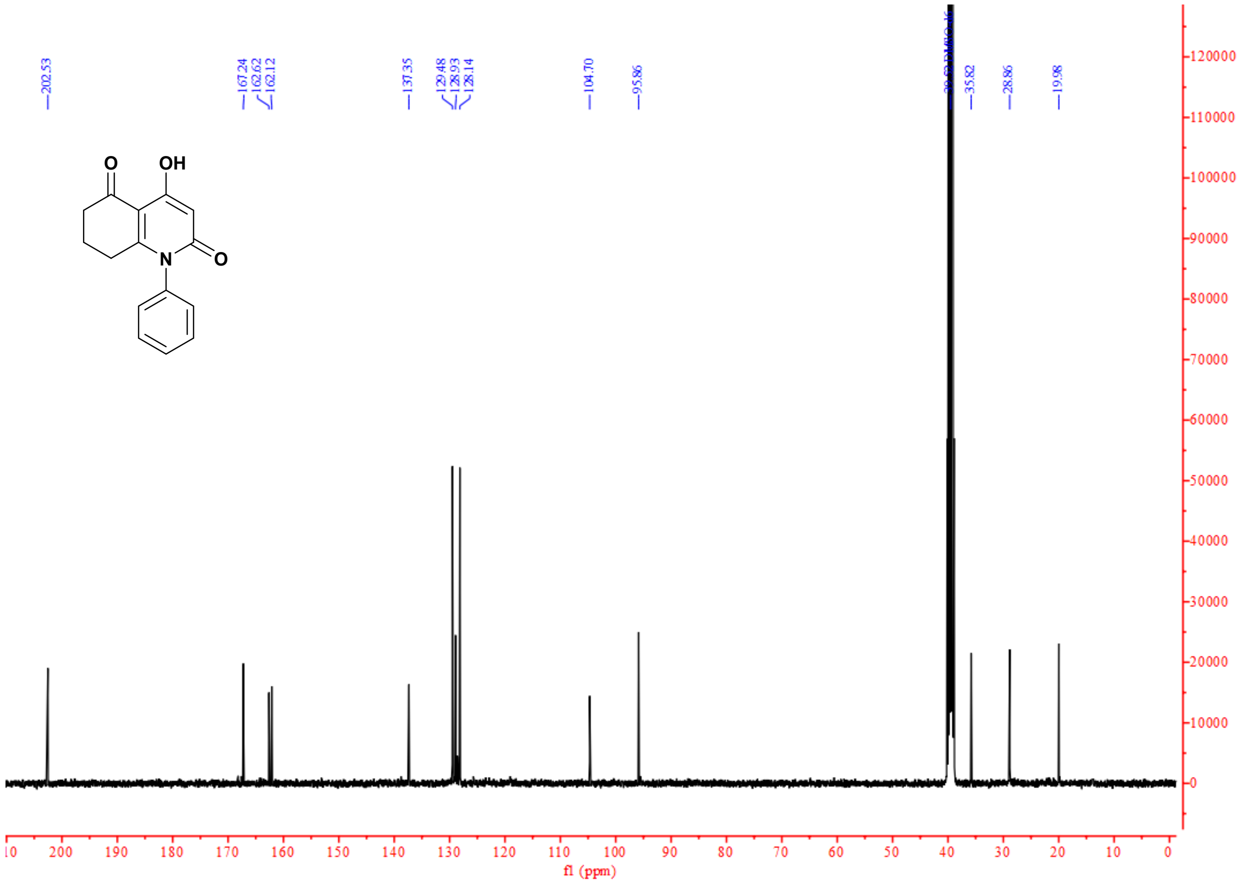

NMR ^{13}C spectrum (101 MHz, DMSO-d_6): 4-hydroxy-1-phenyl-7,8-dihydroquinoline-2,5(1H,6H)-dione

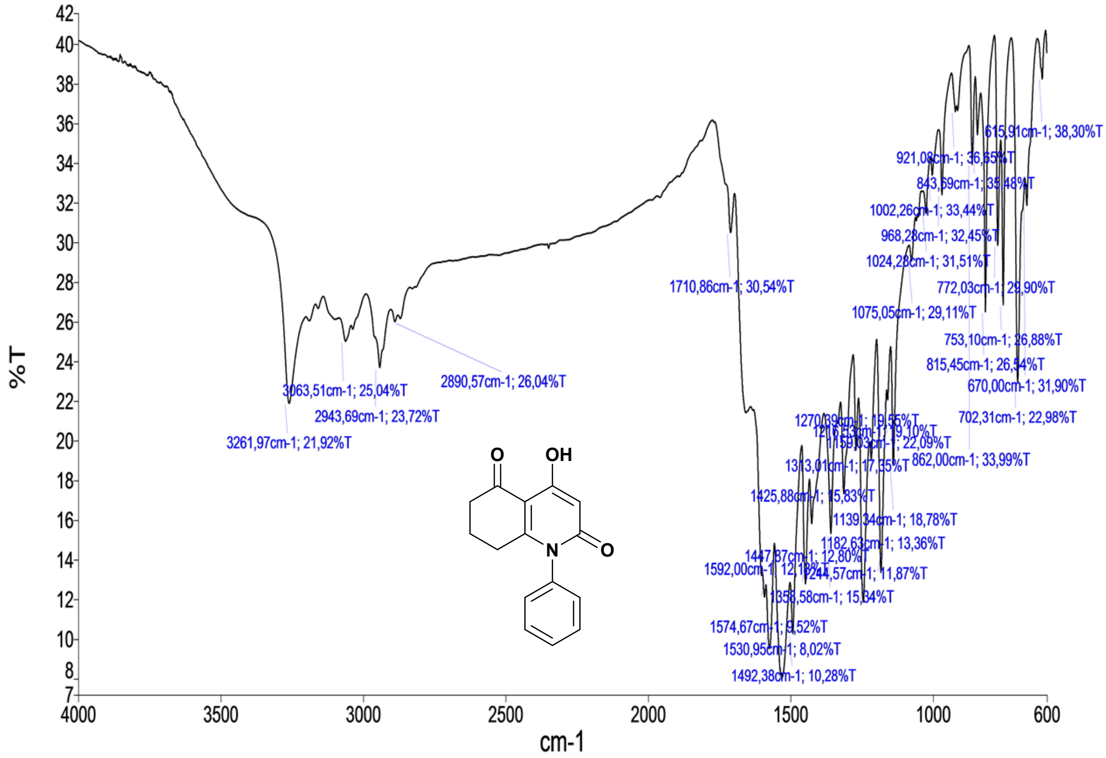

IR spectrum: 4-hydroxy-1-phenyl-7,8-dihydroquinoline-2,5(1H,6H)-dione

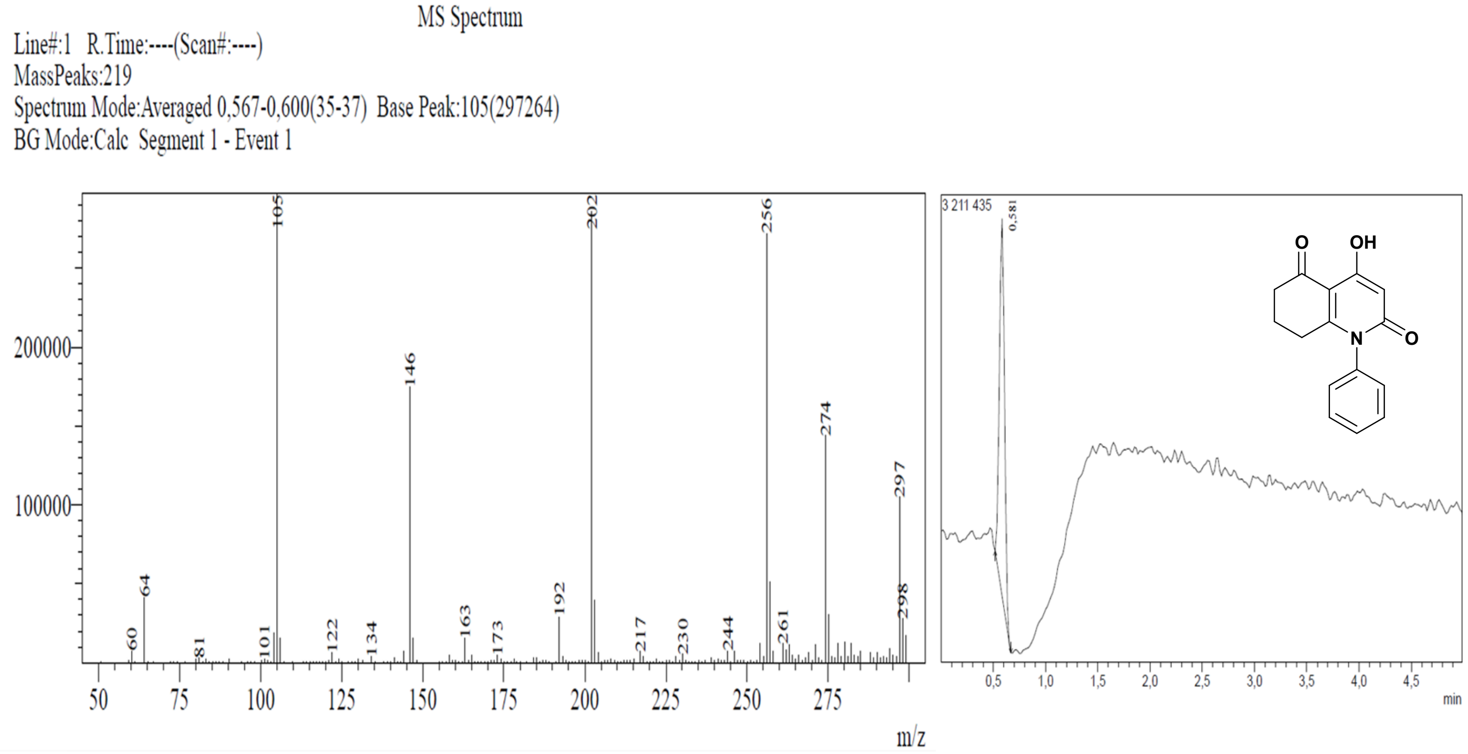

LC-MS spectrum: 4-hydroxy-1-phenyl-7,8-dihydroquinoline-2,5(1H,6H)-dione

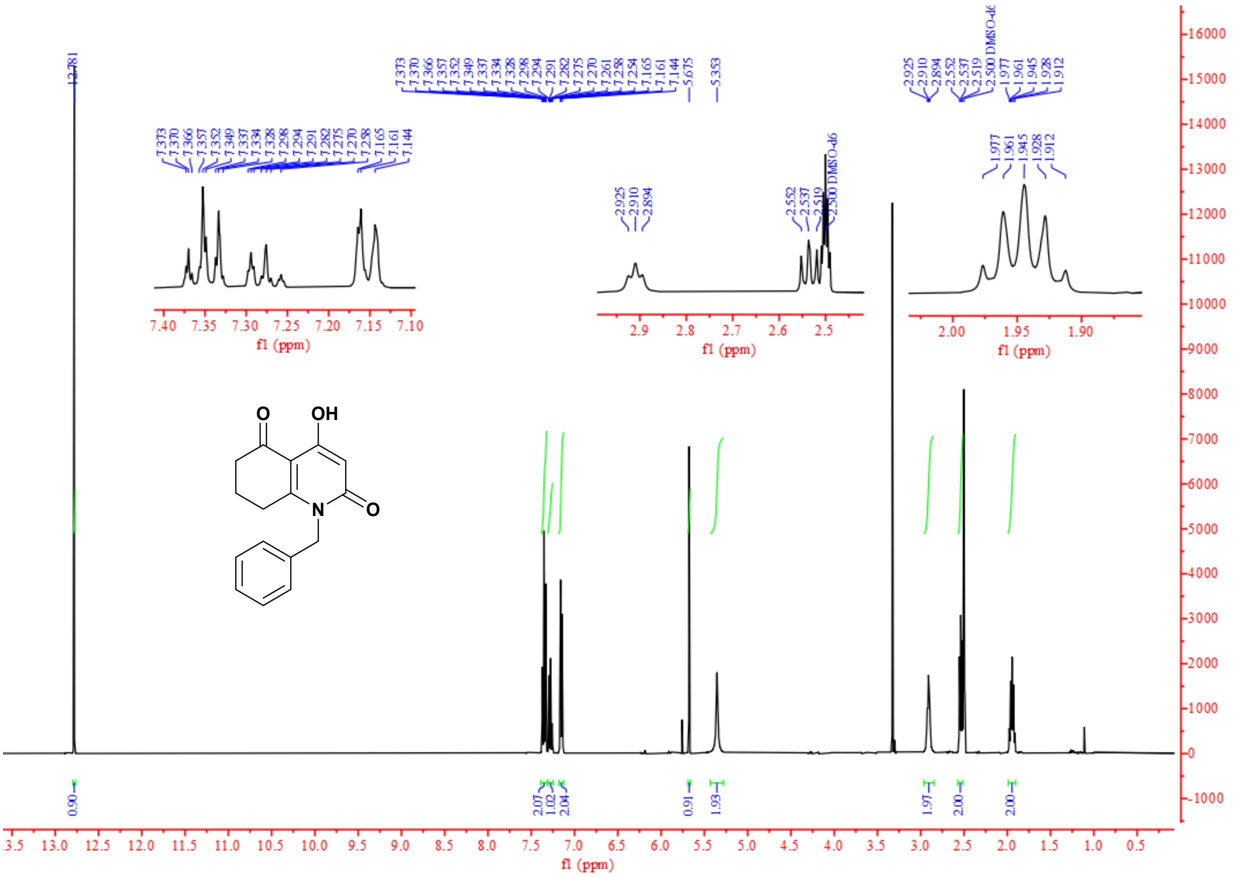

NMR ^{1}H spectrum (400 MHz, DMSO-d$_6$): 1-benzyl-4-hydroxy-7,8-dihydroquinoline-2,5(1H,6H)-dione

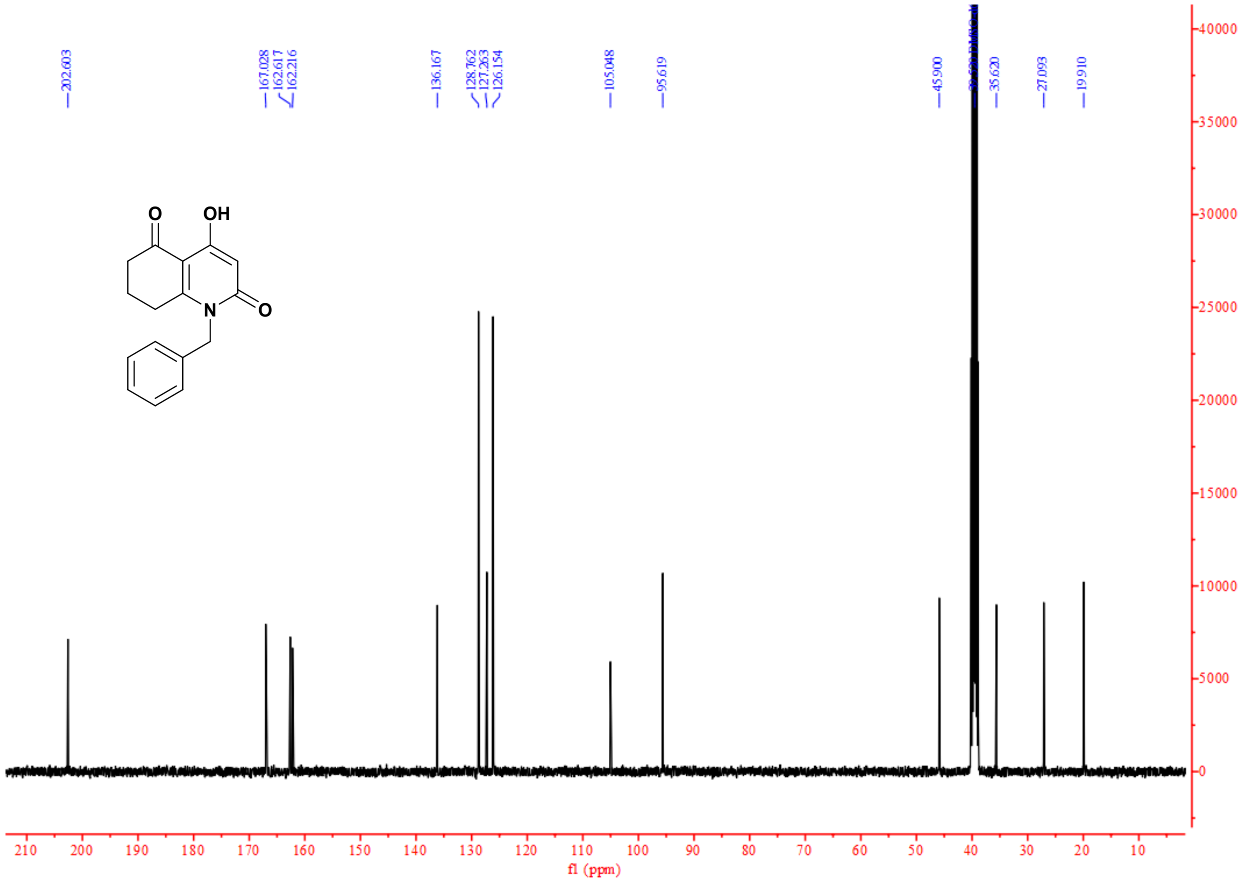

NMR ^{13}C spectrum (101 MHz, DMSO-d$_6$): 1-benzyl-4-hydroxy-7,8-dihydroquinoline-2,5(1H,6H)-dione

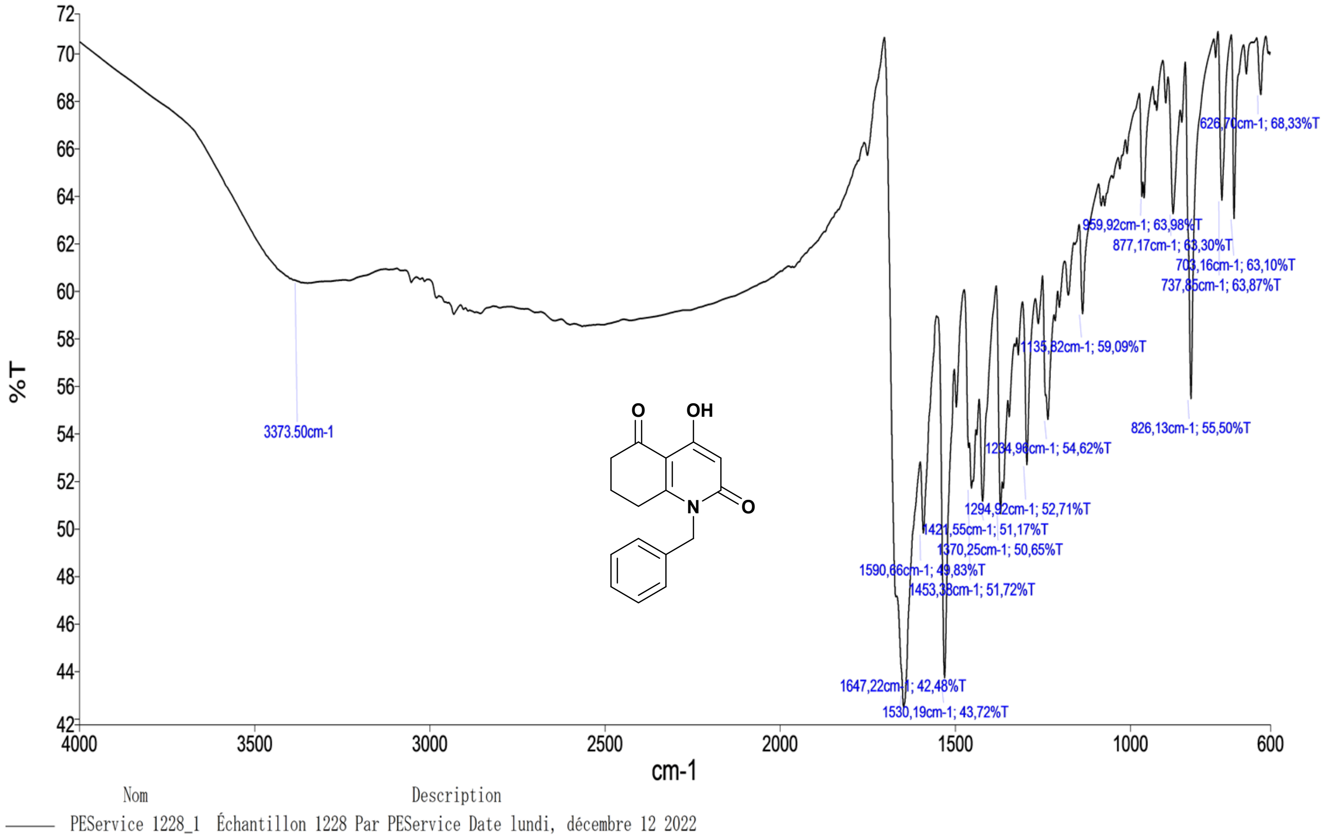

IR spectrum: 1-benzyl-4-hydroxy-7,8-dihydroquinoline-2,5(1H,6H)-dione

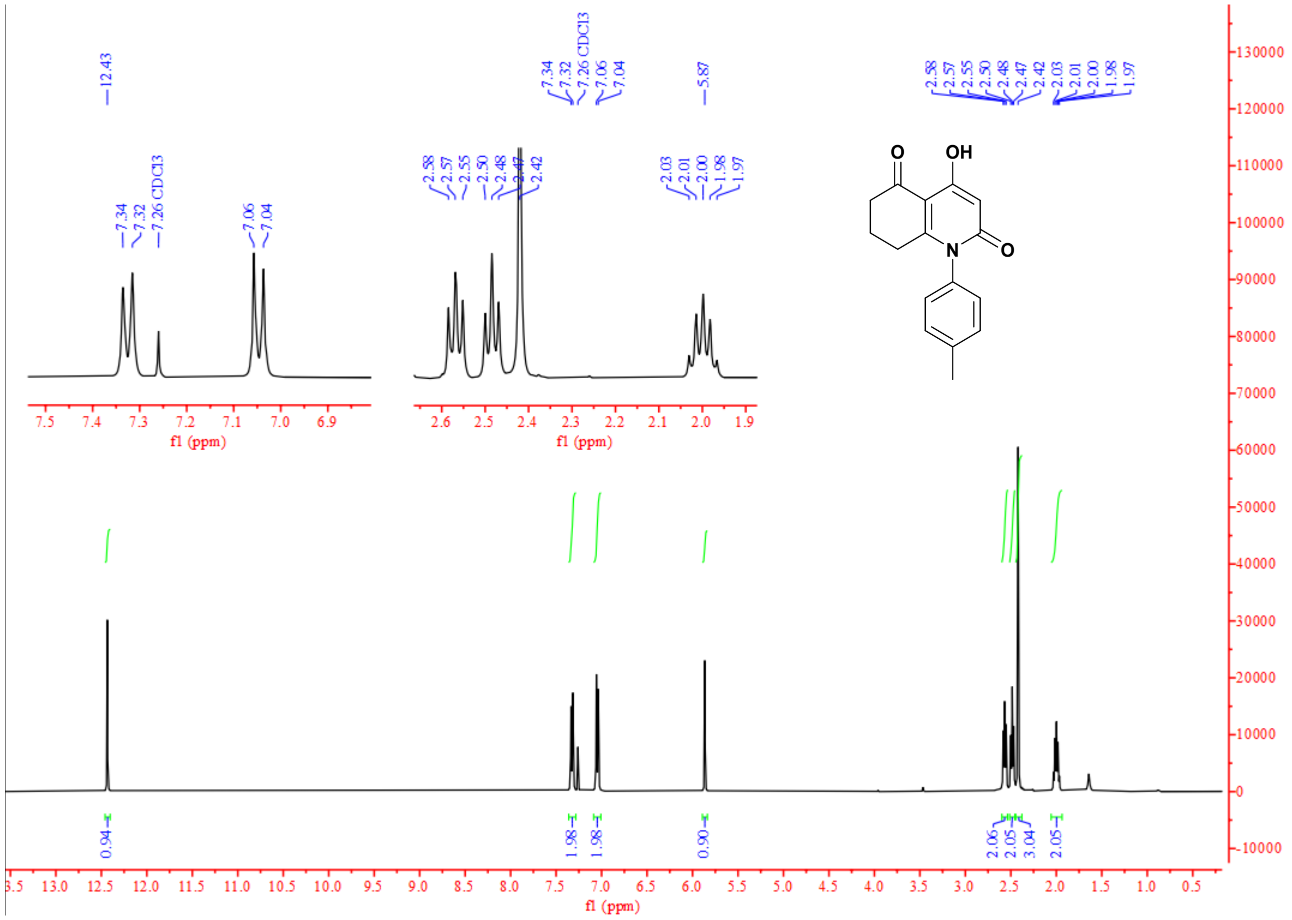

NMR ^{1}H spectrum (400 MHz, $CDCl_3$): 4-hydroxy-1-(p-tolyl)-7,8-dihydroquinoline-2,5(1H,6H)-dione

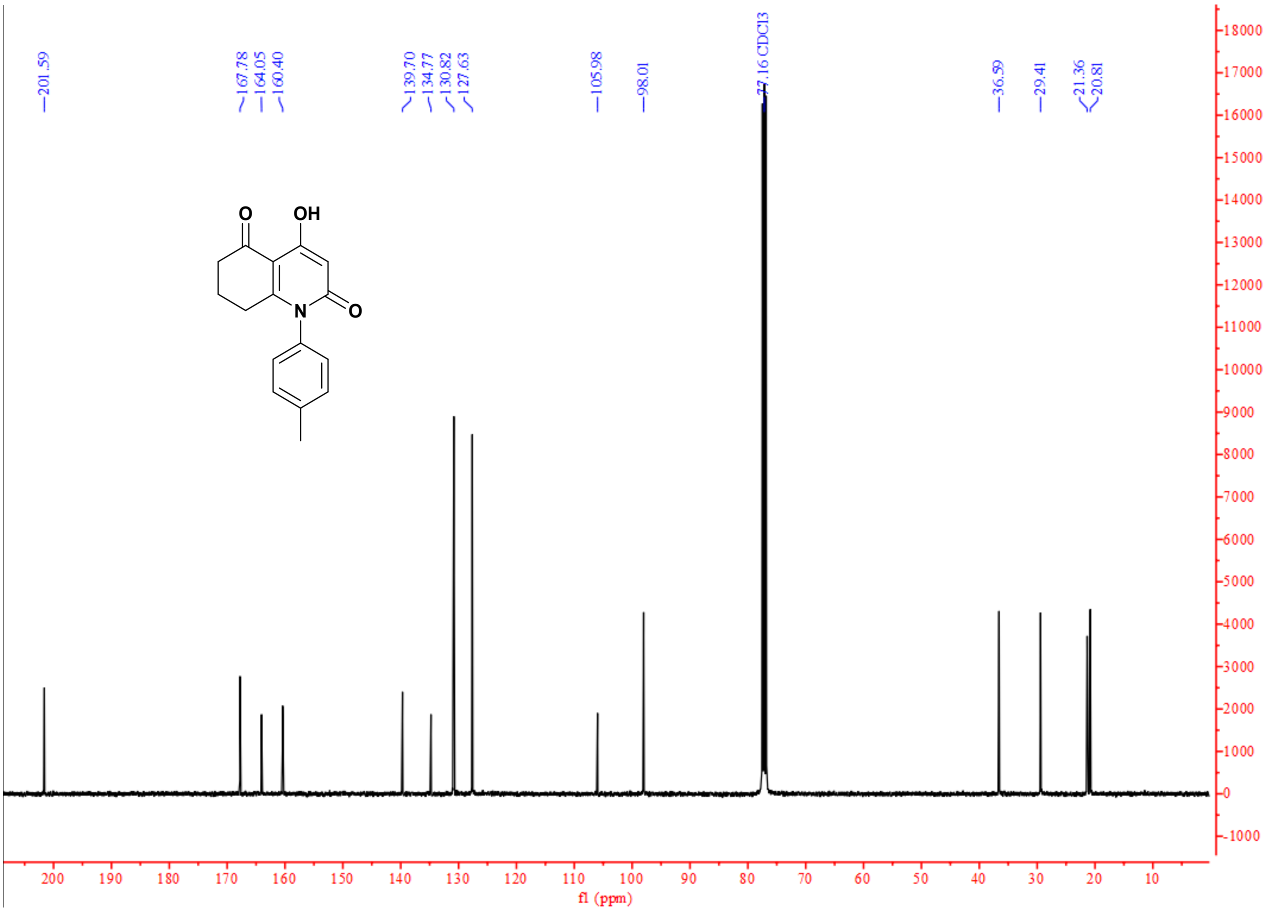

NMR ^{13}C spectrum (101 MHz, $CDCl_3$): 4-hydroxy-1-(p-tolyl)-7,8-dihydroquinoline-2,5(1H,6H)-dione

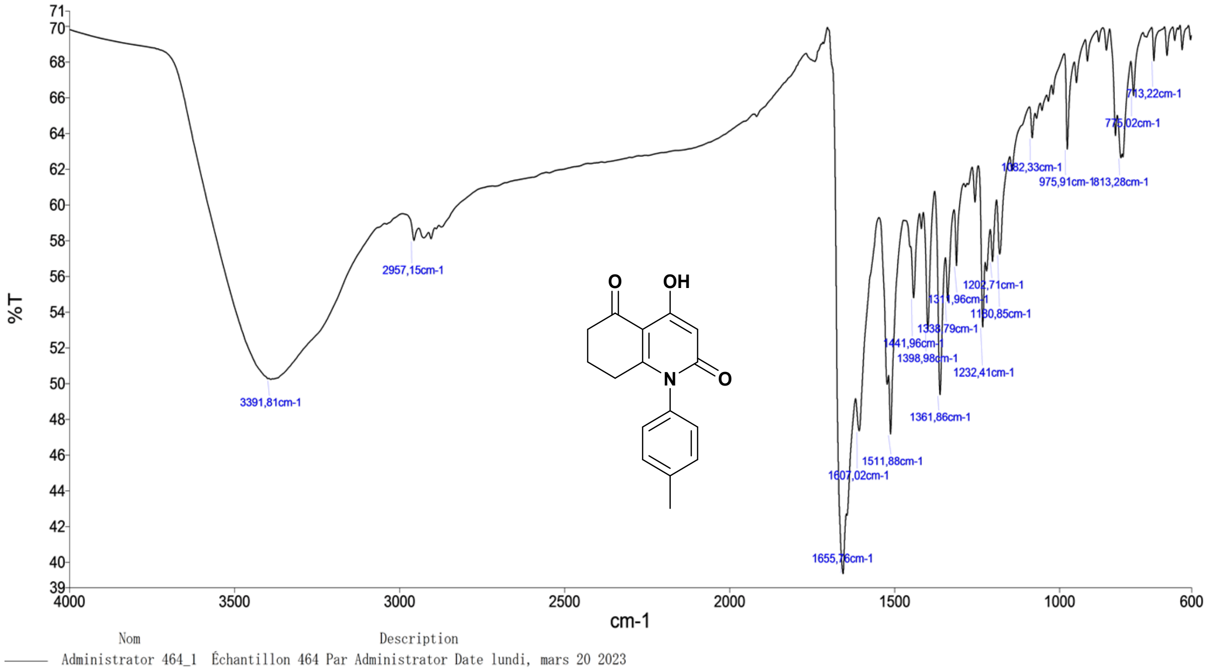

IR spectrum: 4-hydroxy-1-(p-tolyl)-7,8-dihydroquinoline-2,5(1H,6H)-dione

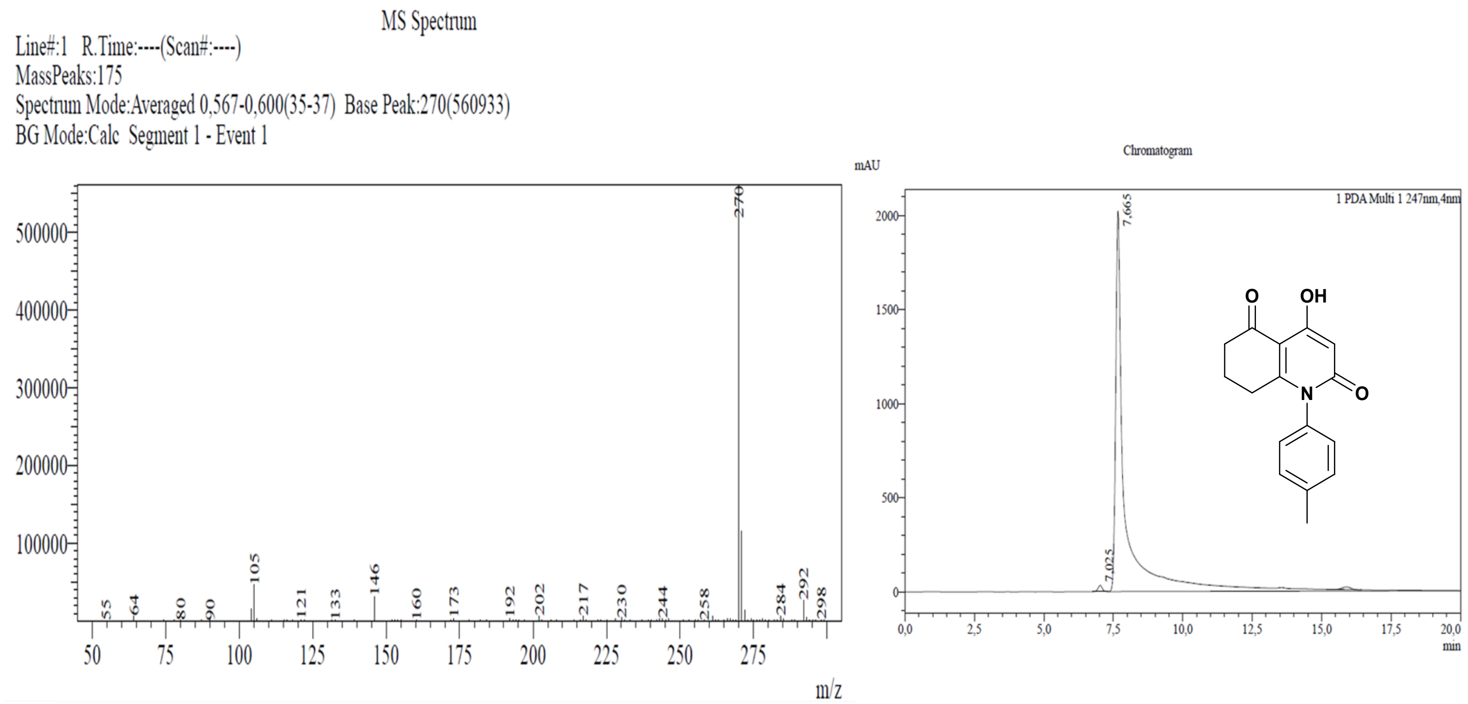

LC-MS spectrum: 4-hydroxy-1-(p-tolyl)-7,8-dihydroquinoline-2,5(1H,6H)-dione

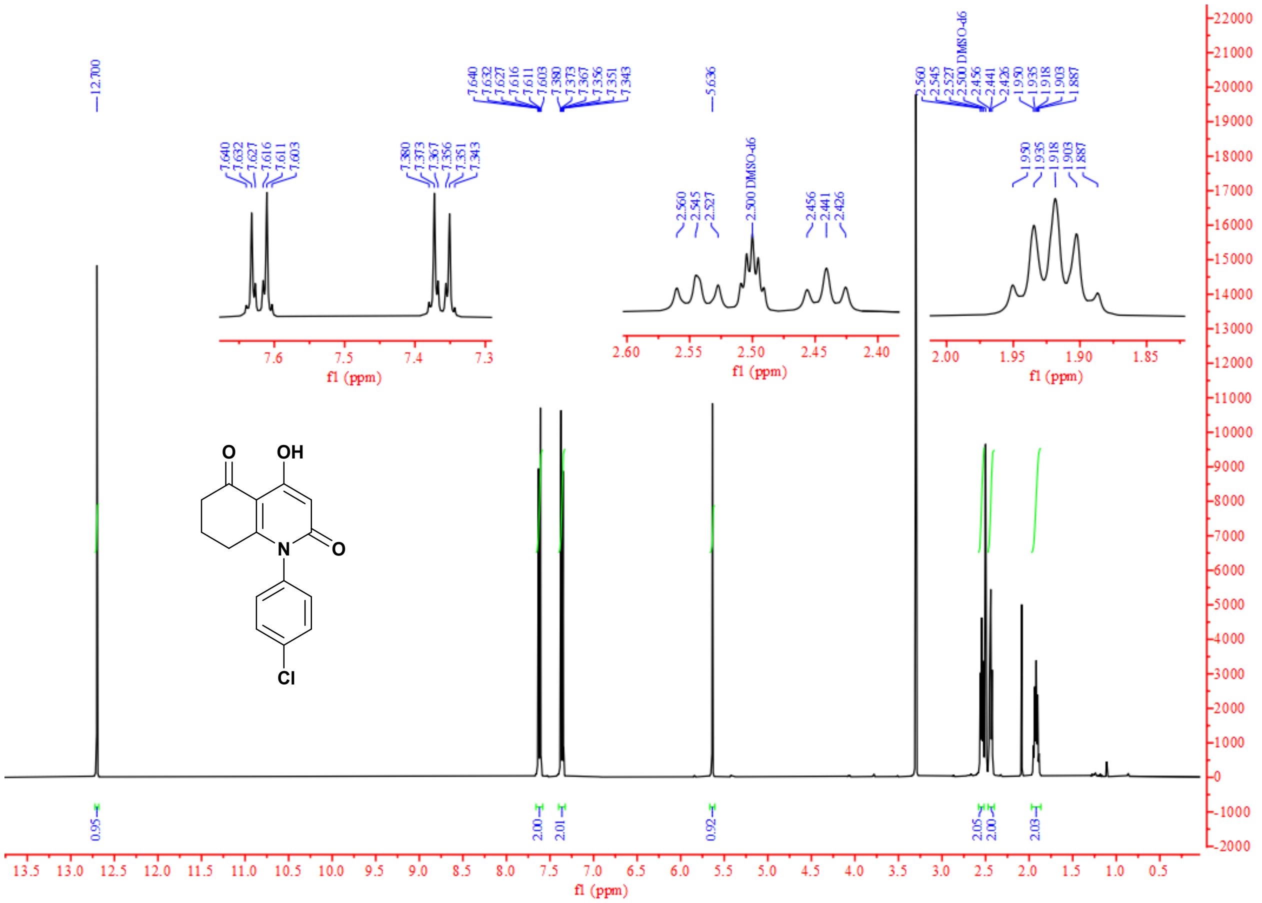

NMR ^{1}H spectrum (400 MHz, DMSO-d$_6$): 1-(4-chlorophenyl)-4-hydroxy-7,8-dihydroquinoline-2,5(1H,6H)-dione

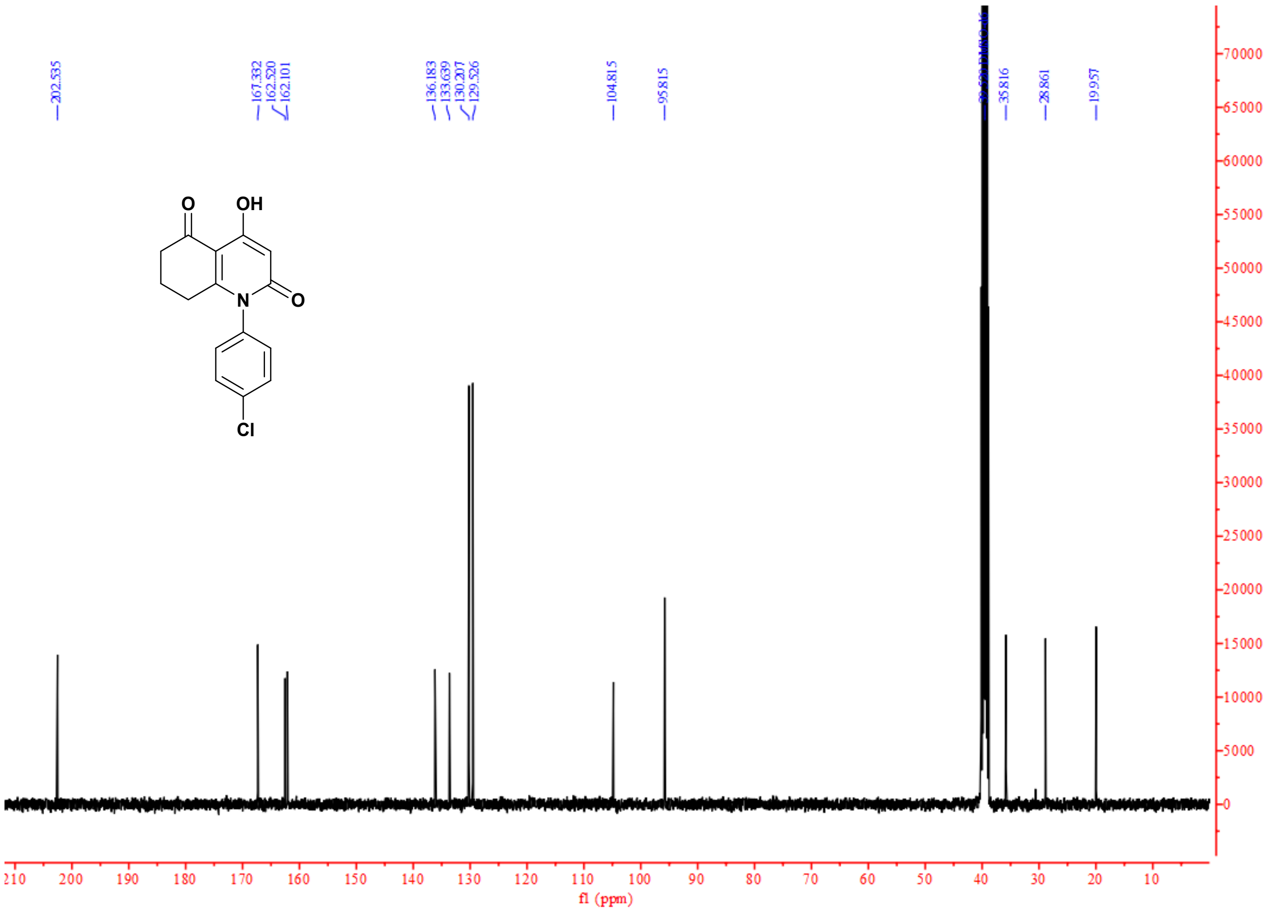

NMR ^{13}C spectrum (101 MHz, DMSO-d$_6$): 1-(4-chlorophenyl)-4-hydroxy-7,8-dihydroquinoline-2,5(1H,6H)-dione

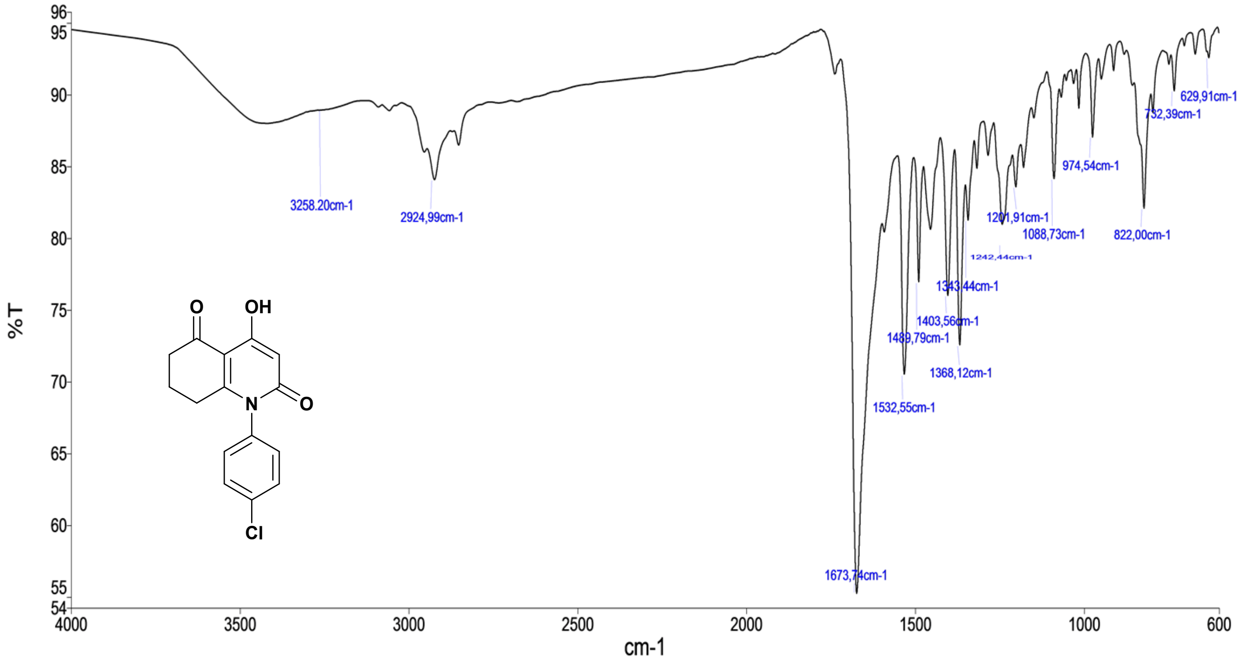

IR spectrum: 1-(4-chlorophenyl)-4-hydroxy-7,8-dihydroquinoline-2,5(1H,6H)-dione

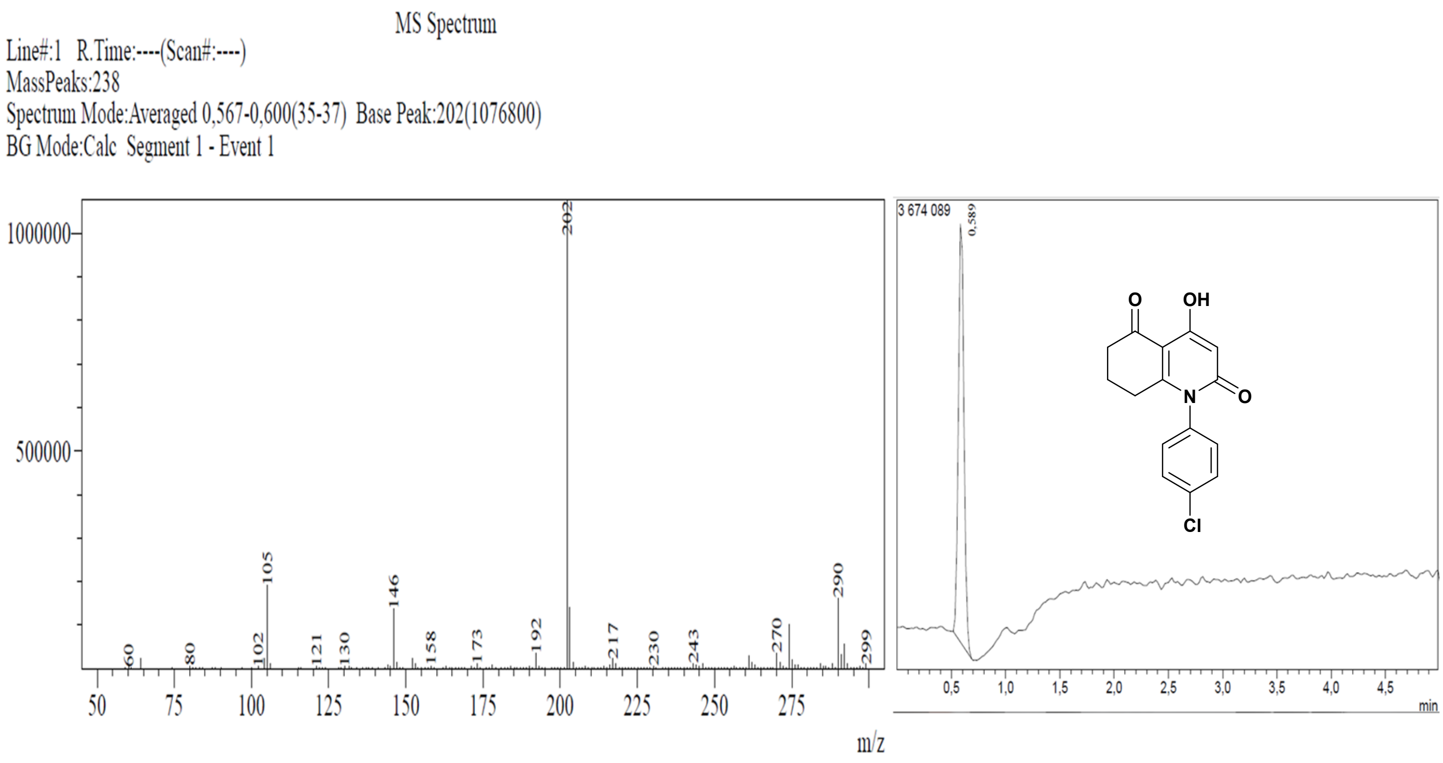

LC-MS spectrum: 1-(4-chlorophenyl)-4-hydroxy-7,8-dihydroquinoline-2,5(1H,6H)-dione

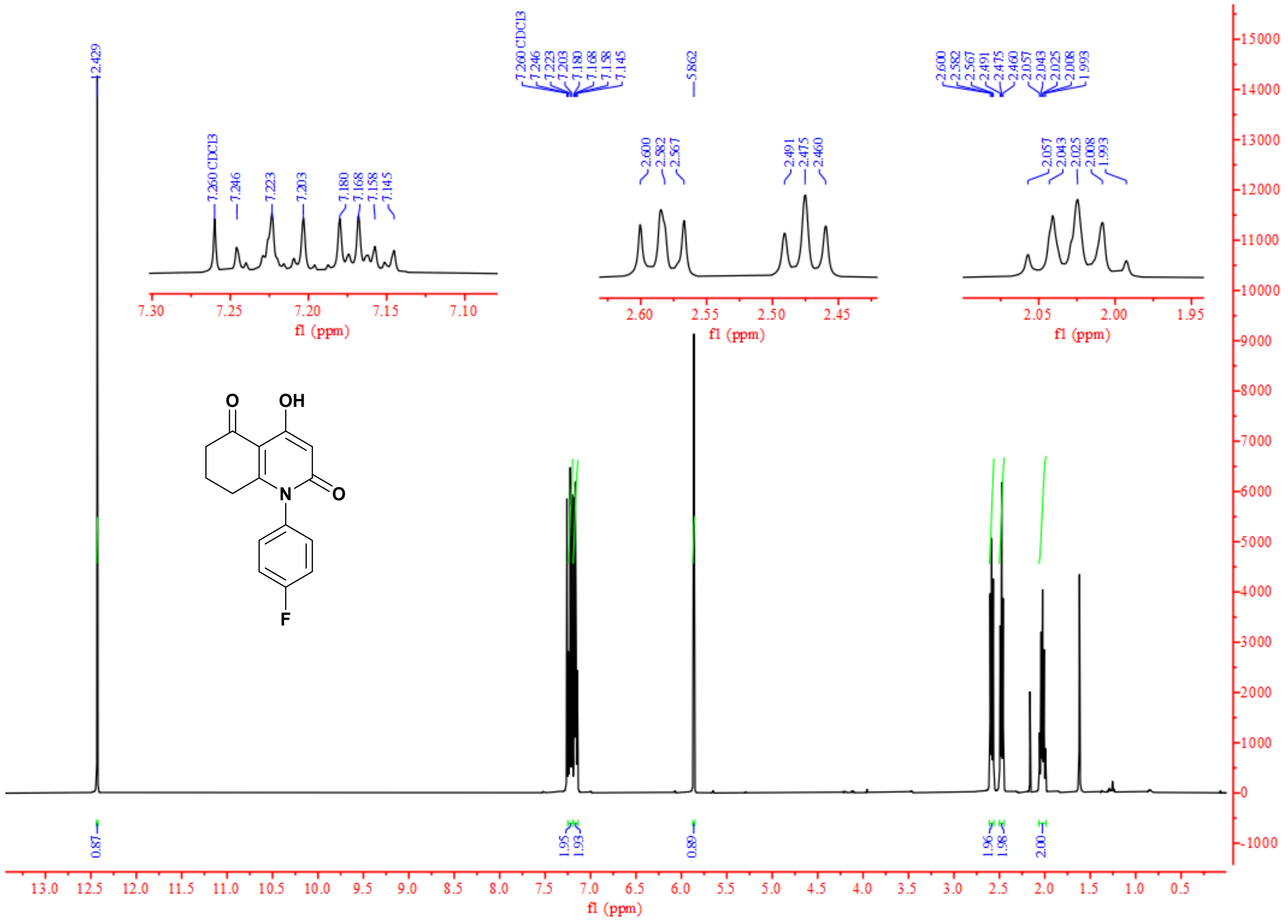

NMR ^{1}H spectrum (400 MHz, $CDCl_3$): 1-(4-fluorophenyl)-4-hydroxy-7,8-dihydroquinoline-2,5(1H,6H)-dione

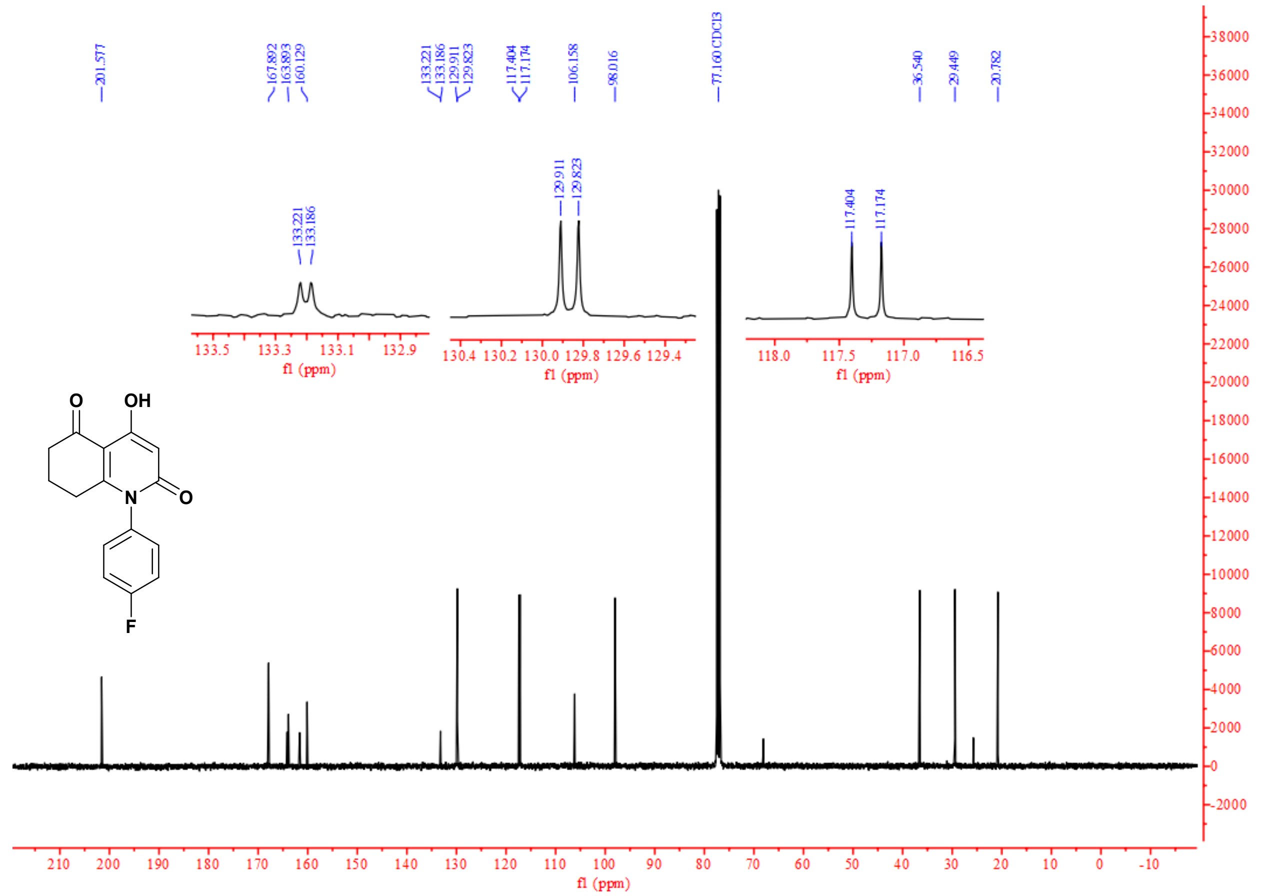

NMR ^{13}C spectrum (101 MHz, $CDCl_3$): 1-(4-fluorophenyl)-4-hydroxy-7,8-dihydroquinoline-2,5(1H,6H)-dione

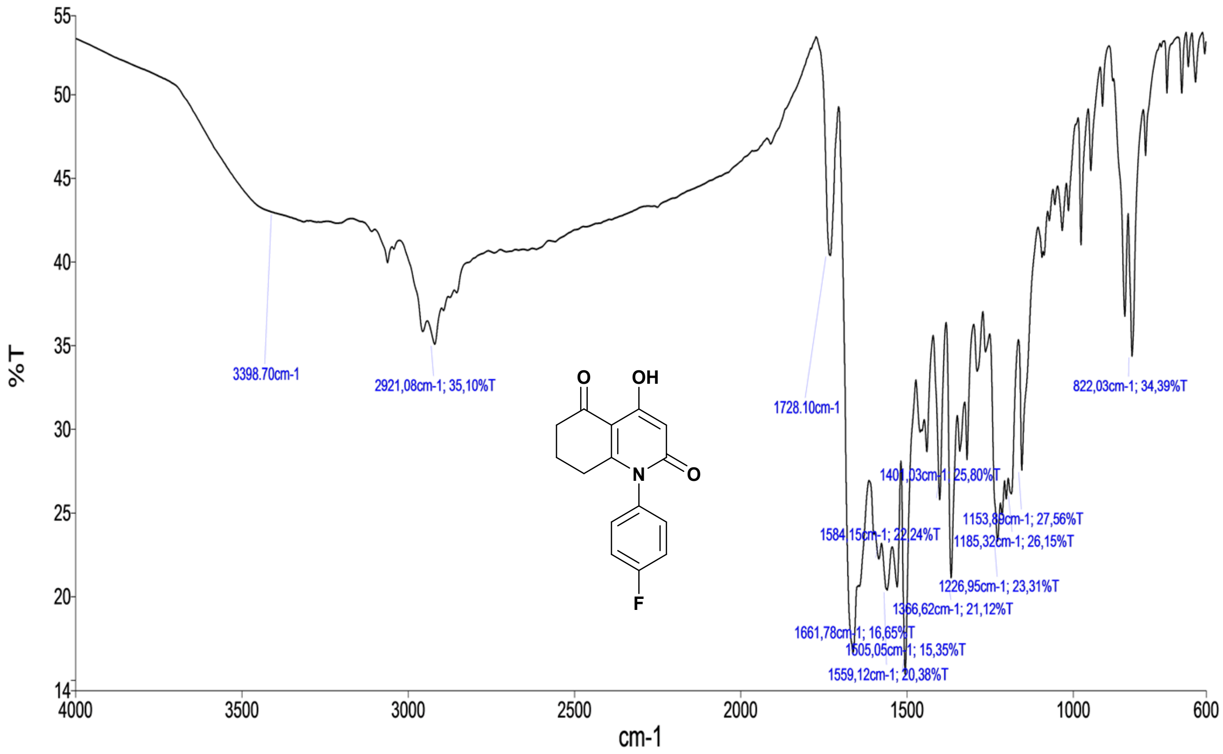

IR spectrum: 1-(4-fluorophenyl)-4-hydroxy-7,8-dihydroquinoline-2,5(1H,6H)-dione

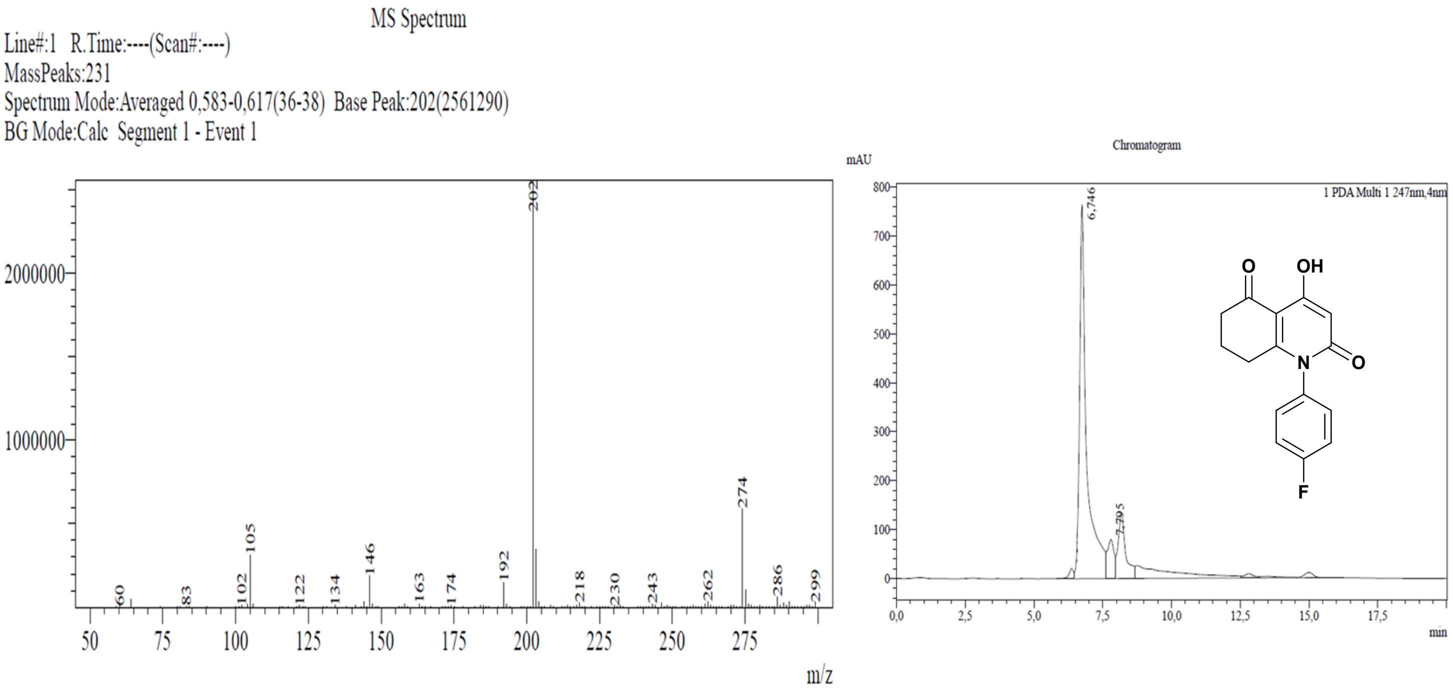

LC-MS spectrum: 1-(4-fluorophenyl)-4-hydroxy-7,8-dihydroquinoline-2,5(1H,6H)-dione

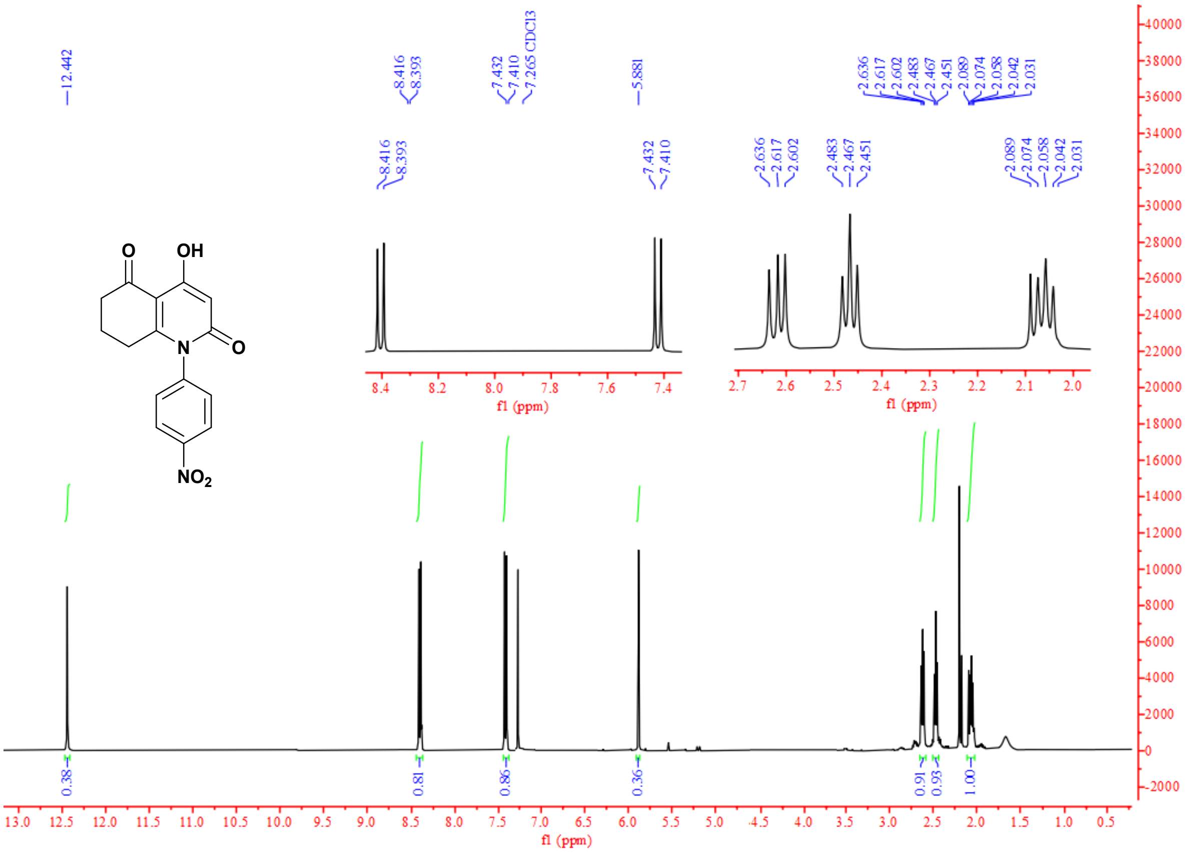

NMR ^{1}H spectrum (400 MHz, $CDCl_3$): 4-hydroxy-1-(4-nitrophenyl)-7,8-dihydroquinoline-2,5(1H,6H)-dione

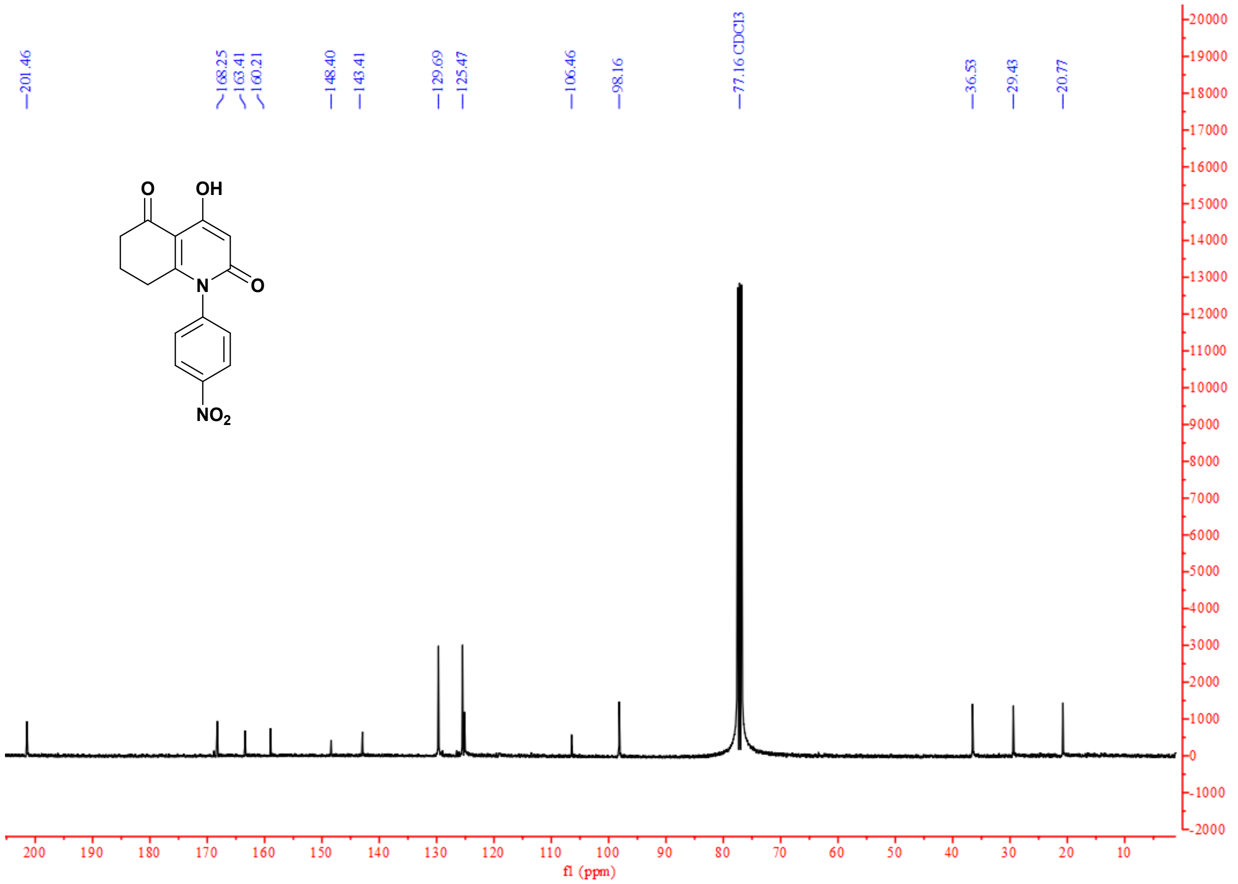

NMR ^{13}C spectrum (101 MHz, $CDCl_3$): 4-hydroxy-1-(4-nitrophenyl)-7,8-dihydroquinoline-2,5(1H,6H)-dione

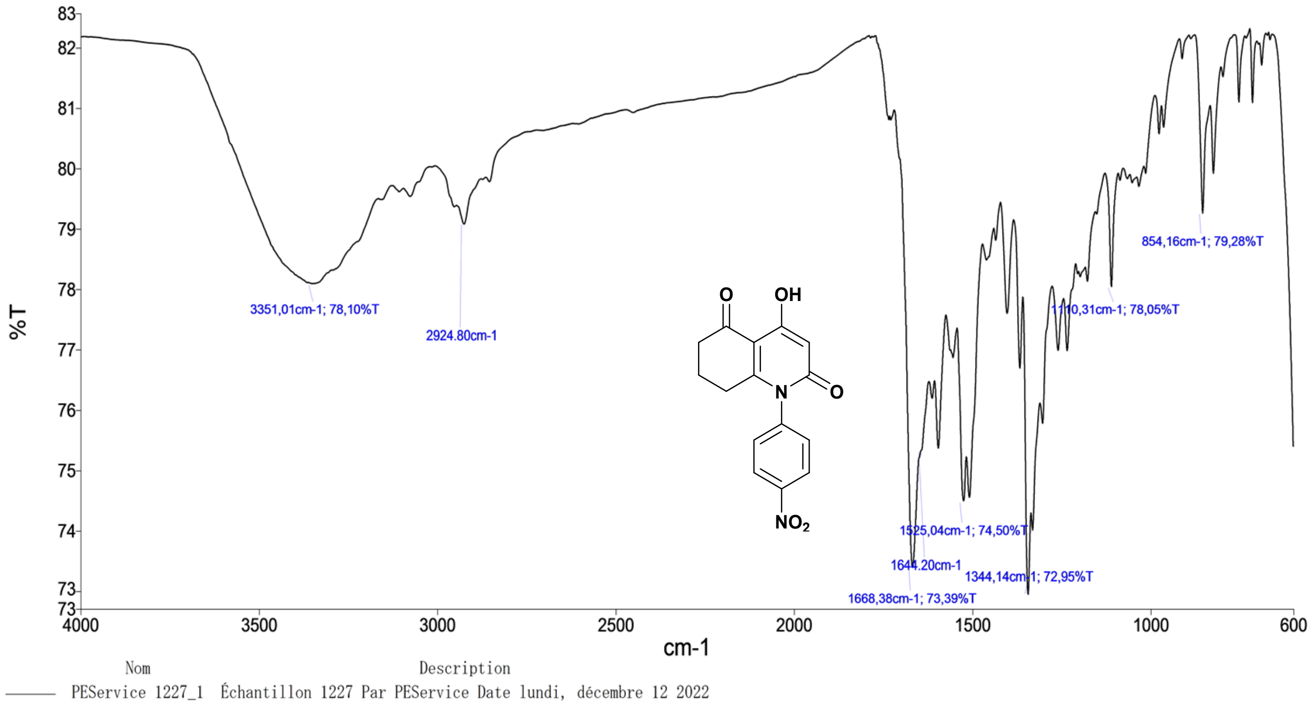

IR spectrum: 4-hydroxy-1-(4-nitrophenyl)-7,8-dihydroquinoline-2,5(1H,6H)-dione

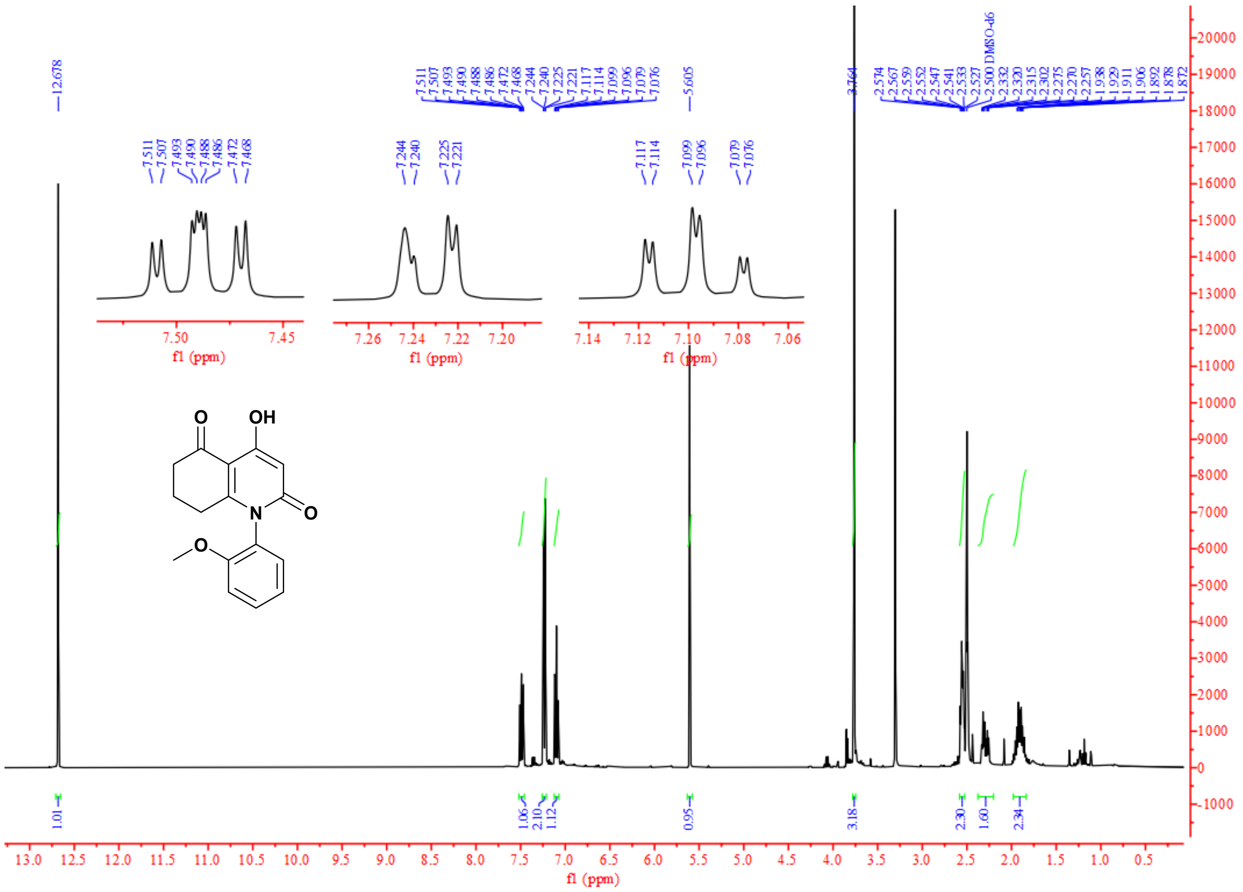

NMR ^{1}H spectrum (400 MHz, DMSO-d$_6$): 4-hydroxy-1-(2-methoxyphenyl)-7,8-dihydroquinoline-2,5(1H,6H)-dione

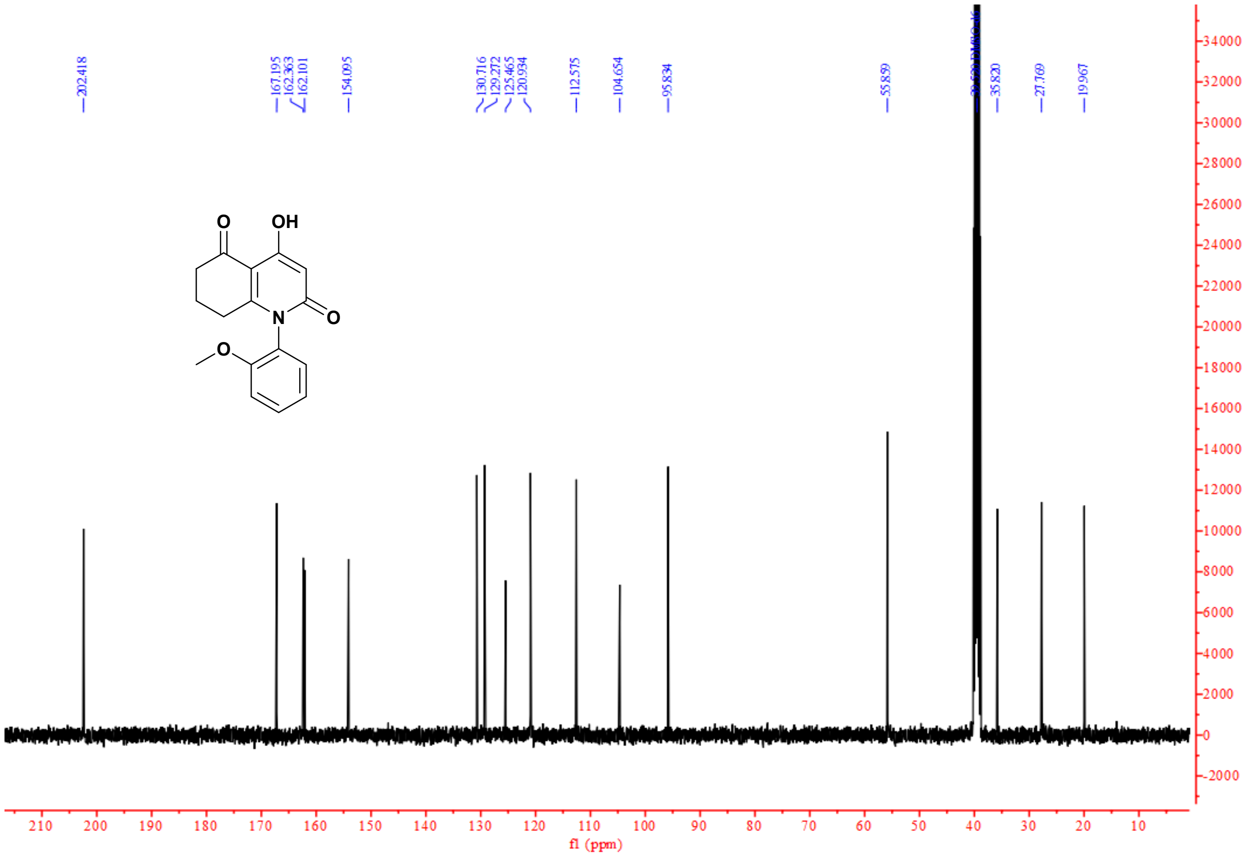

NMR ^{13}C spectrum (101 MHz, DMSO-d$_6$): 4-hydroxy-1-(2-methoxyphenyl)-7,8-dihydroquinoline-2,5(1H,6H)-dione

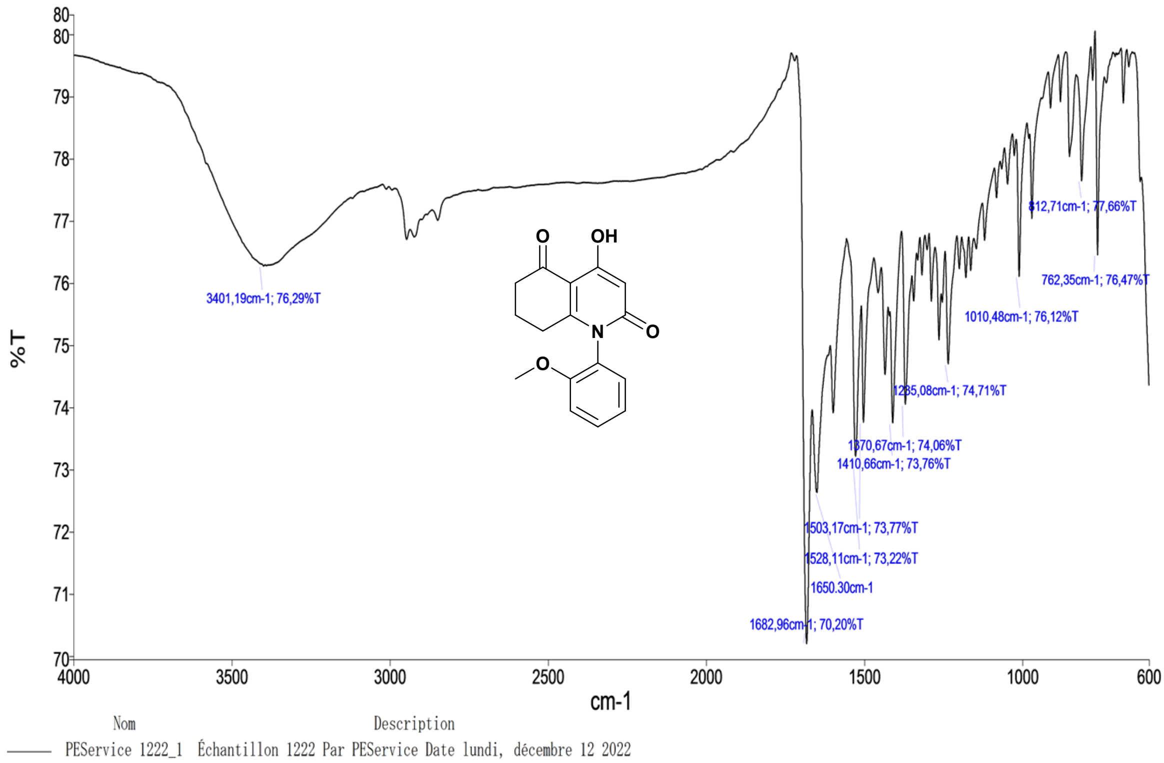

IR spectrum: 4-hydroxy-1-(2-methoxyphenyl)-7,8-dihydroquinoline-2,5(1H,6H)-dione

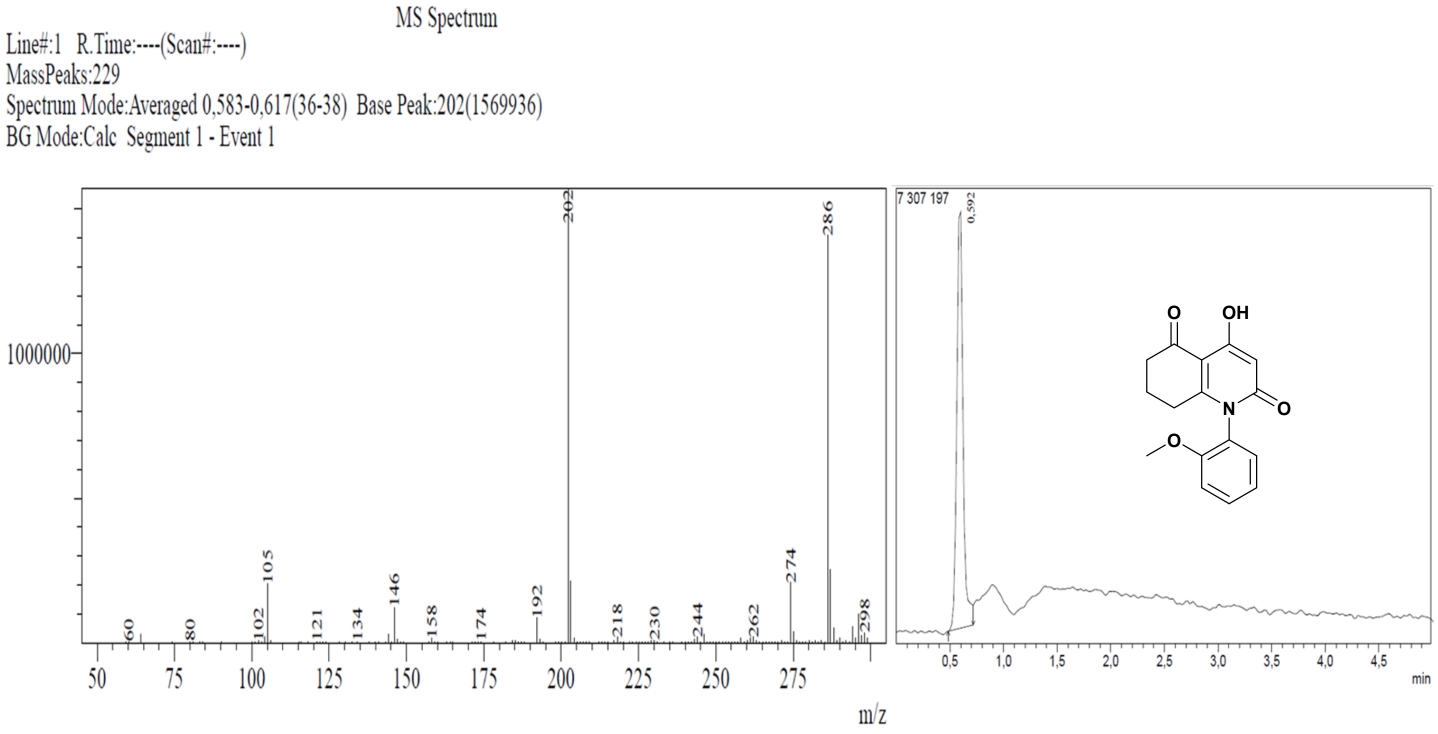

LC-MS spectrum: 4-hydroxy-1-(2-methoxyphenyl)-7,8-dihydroquinoline-2,5(1H,6H)-dione

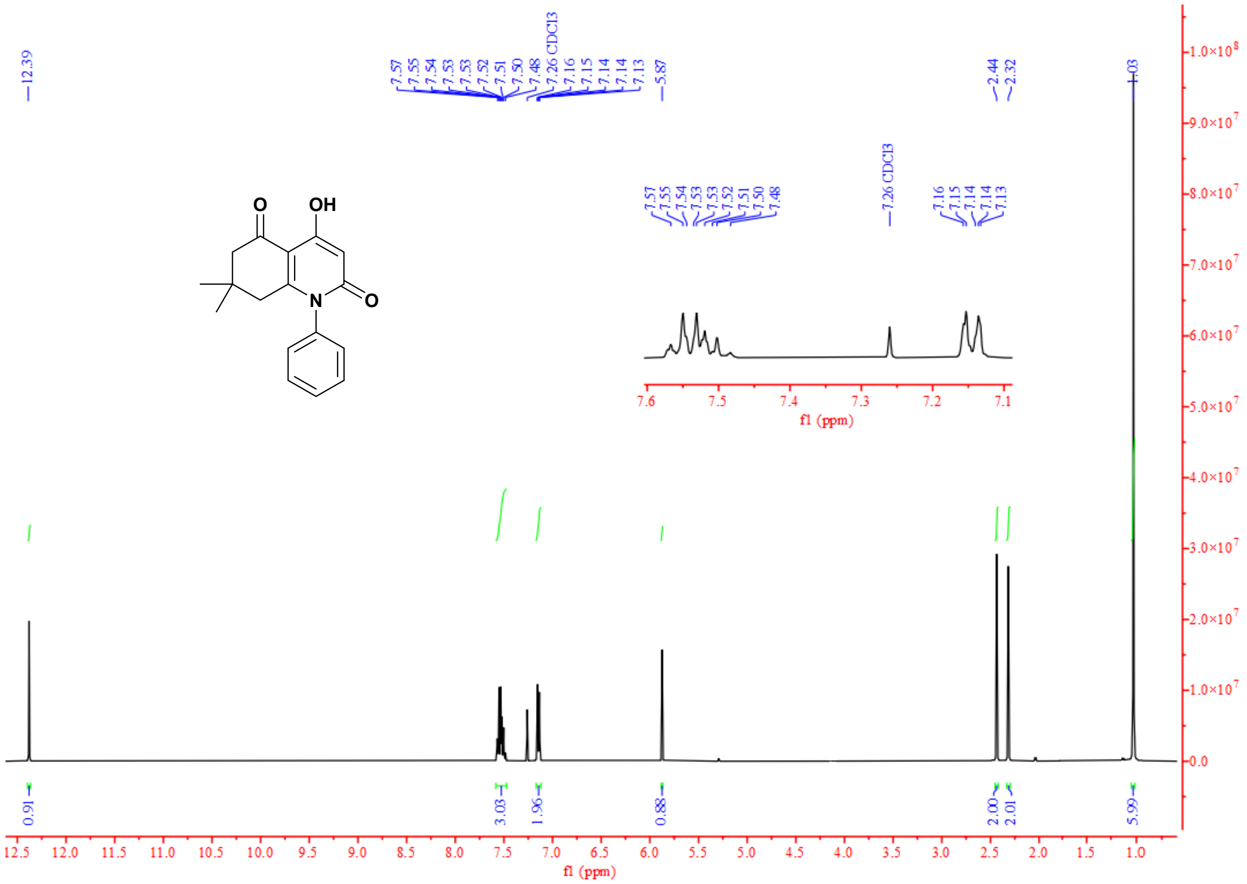

NMR ^{1}H spectrum (400 MHz, $CDCl_3$): 4-hydroxy-7,7-dimethyl-1-phenyl-7,8-dihydroquinoline-2,5(1H,6H)-dione

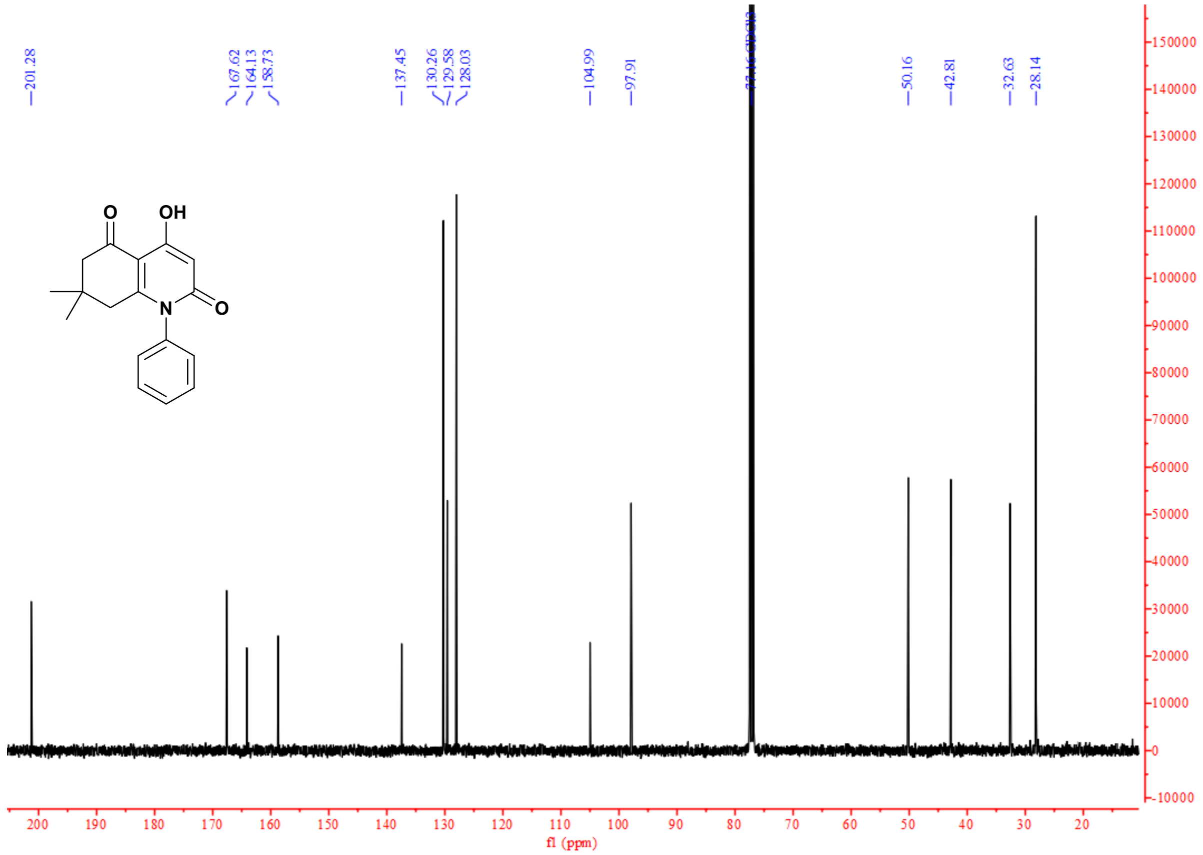

NMR ^{13}C spectrum (101 MHz, $CDCl_3$): 4-hydroxy-7,7-dimethyl-1-phenyl-7,8-dihydroquinoline-2,5(1H,6H)-dione

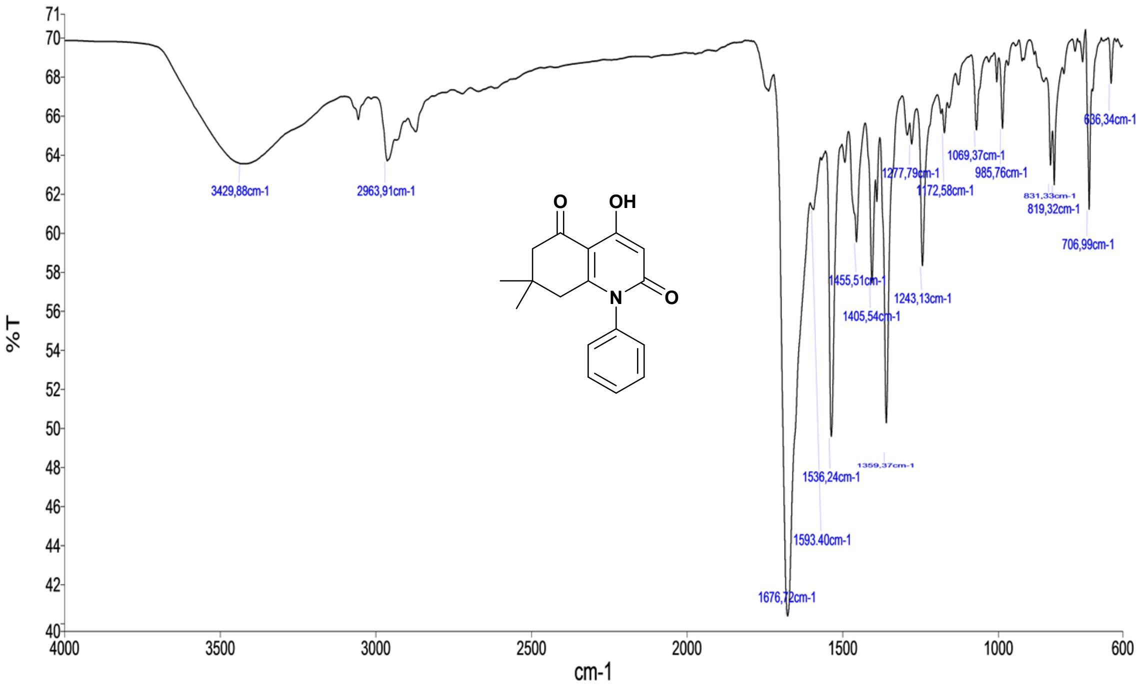

IR spectrum: 4-hydroxy-7,7-dimethyl-1-phenyl-7,8-dihydroquinoline-2,5(1H,6H)-dione

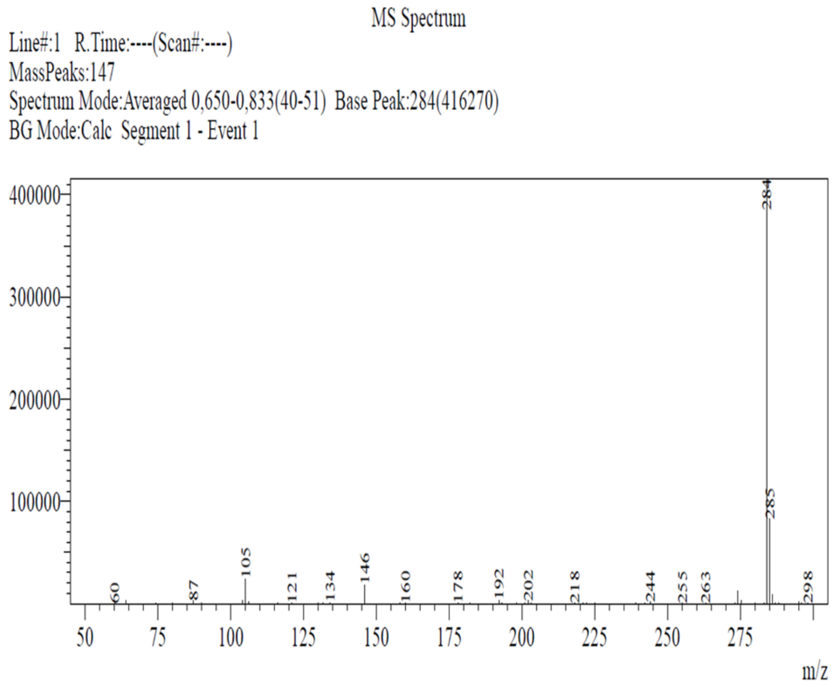

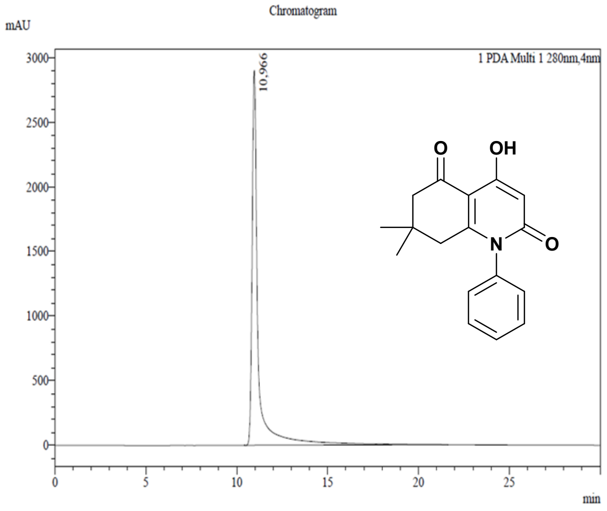

LC-MS spectrum: 4-hydroxy-7,7-dimethyl-1-phenyl-7,8-dihydroquinoline-2,5(1H,6H)-dione

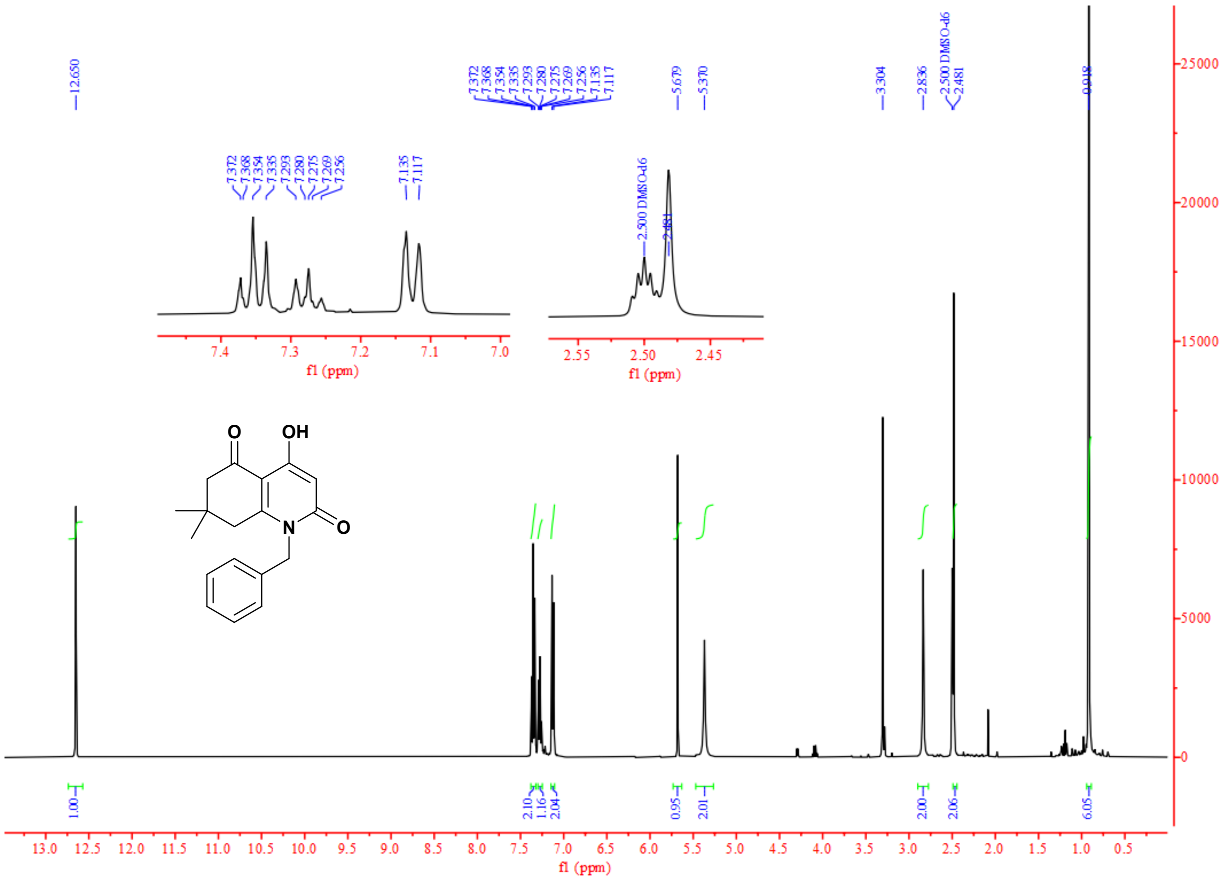

NMR ^{1}H spectrum (400 MHz, DMSO-d$_6$): 1-benzyl-4-hydroxy-7,7-dimethyl-7,8-dihydroquinoline-2,5(1H,6H)-dione

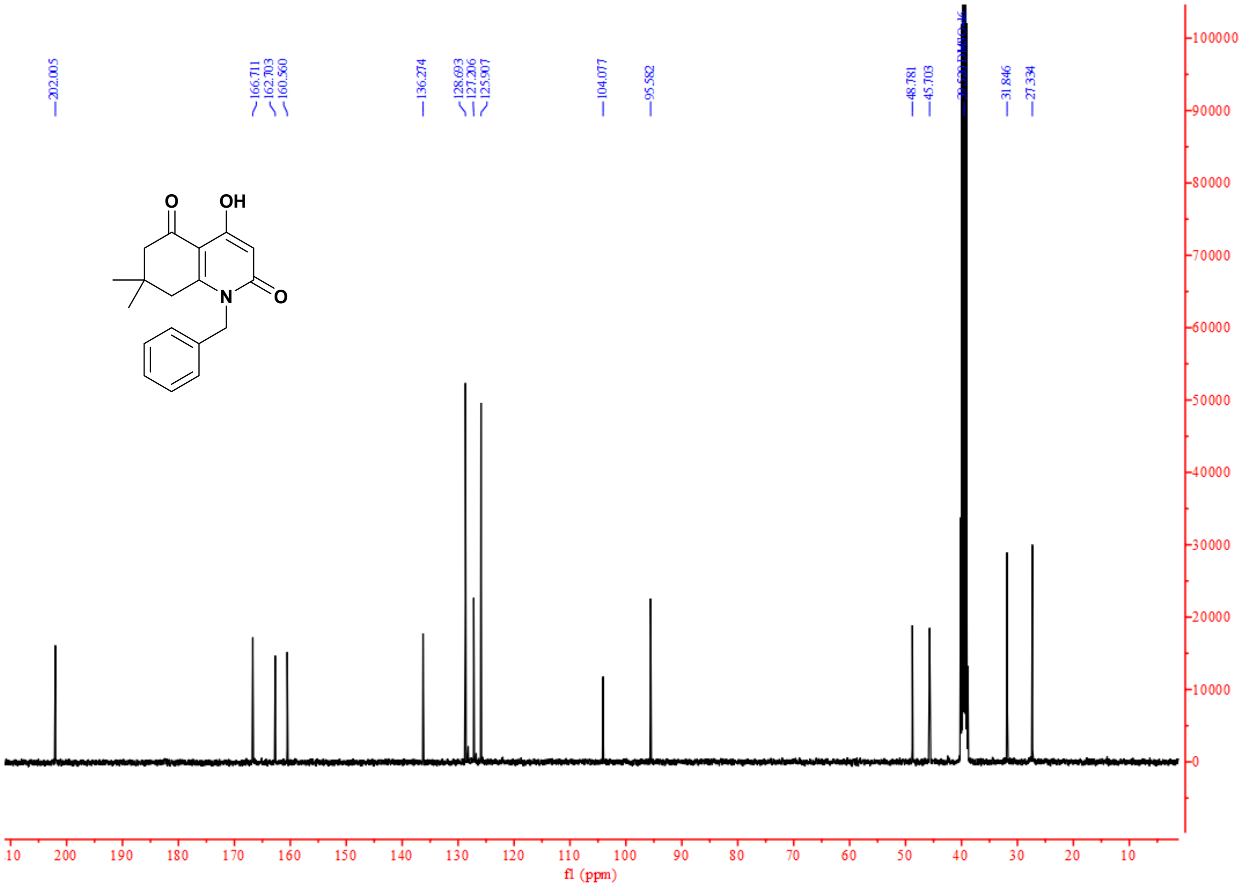

NMR ^{13}C spectrum (101 MHz, DMSO-d$_6$): 1-benzyl-4-hydroxy-7,7-dimethyl-7,8-dihydroquinoline-2,5(1H,6H)-dione

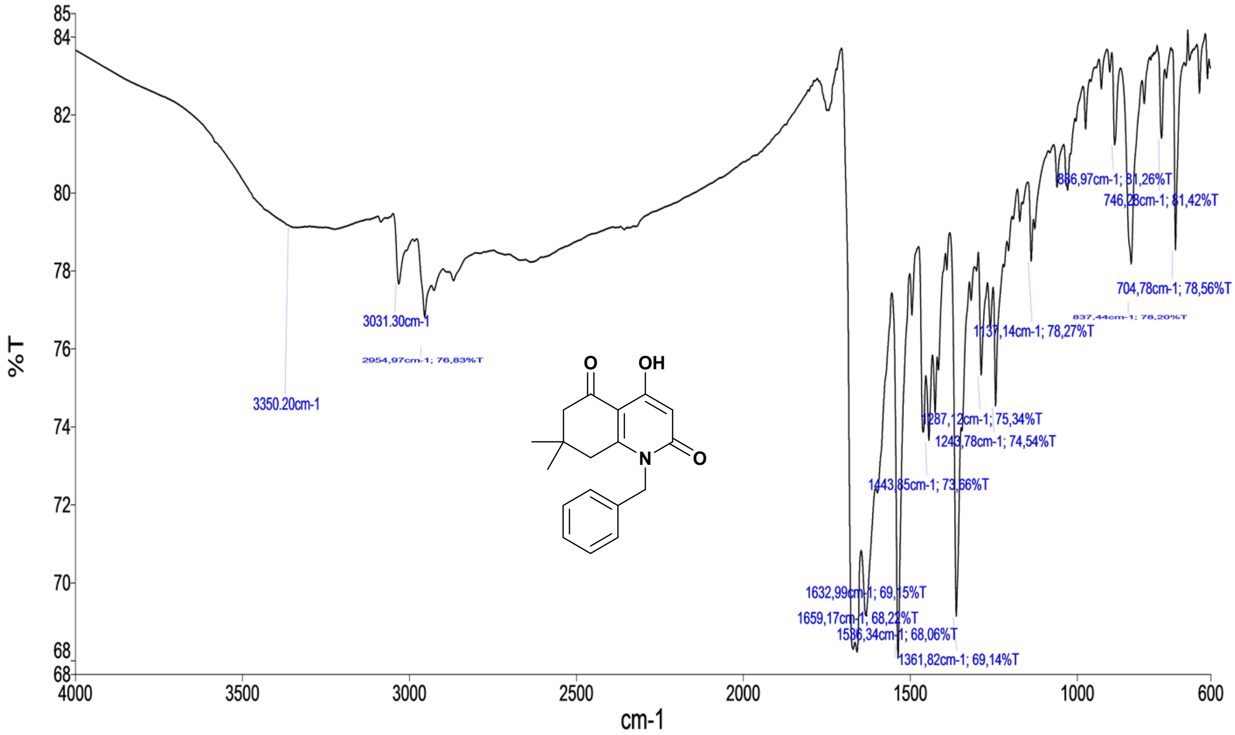

IR spectrum: 1-benzyl-4-hydroxy-7,7-dimethyl-7,8-dihydroquinoline-2,5(1H,6H)-dione

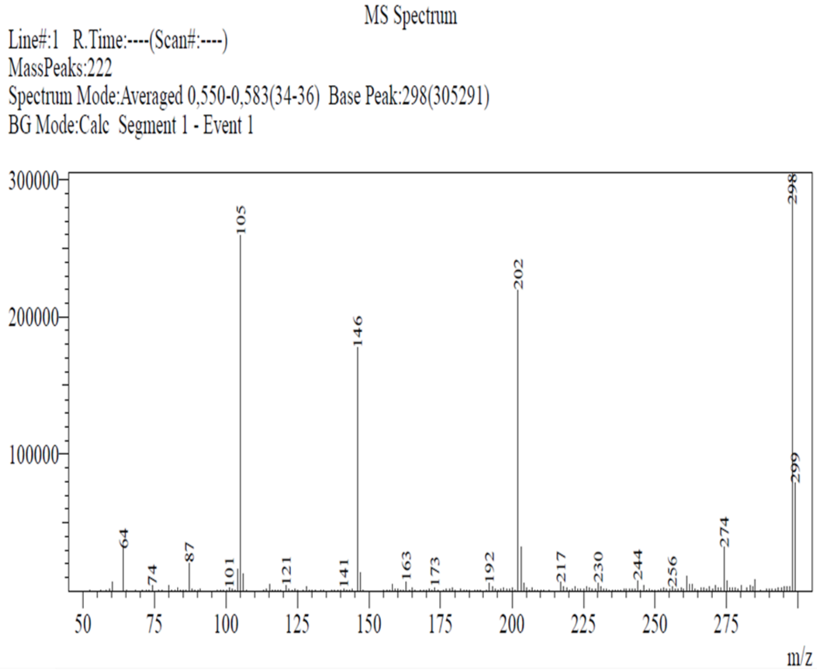

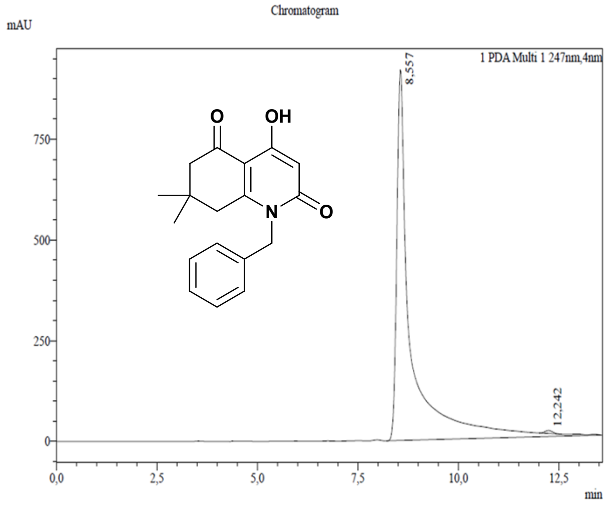

LC-MS spectrum: 1-benzyl-4-hydroxy-7,7-dimethyl-7,8-dihydroquinoline-2,5(1H,6H)-dione

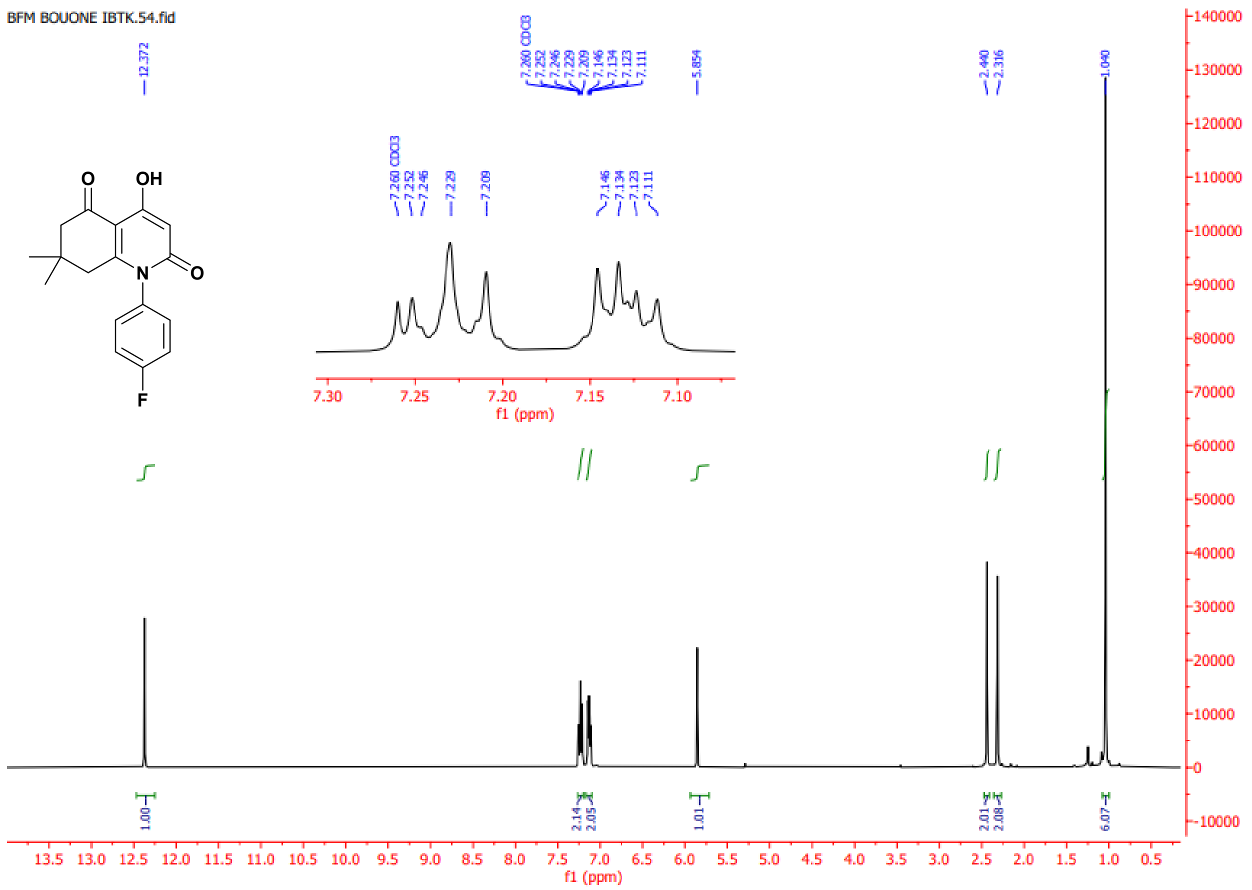

NMR ^{1}H spectrum (400 MHz, $CDCl_3$): 1-(4-fluorophenyl)-4-hydroxy-7,7-dimethyl-7,8-dihydroquinoline-2,5(1H,6H)-dione

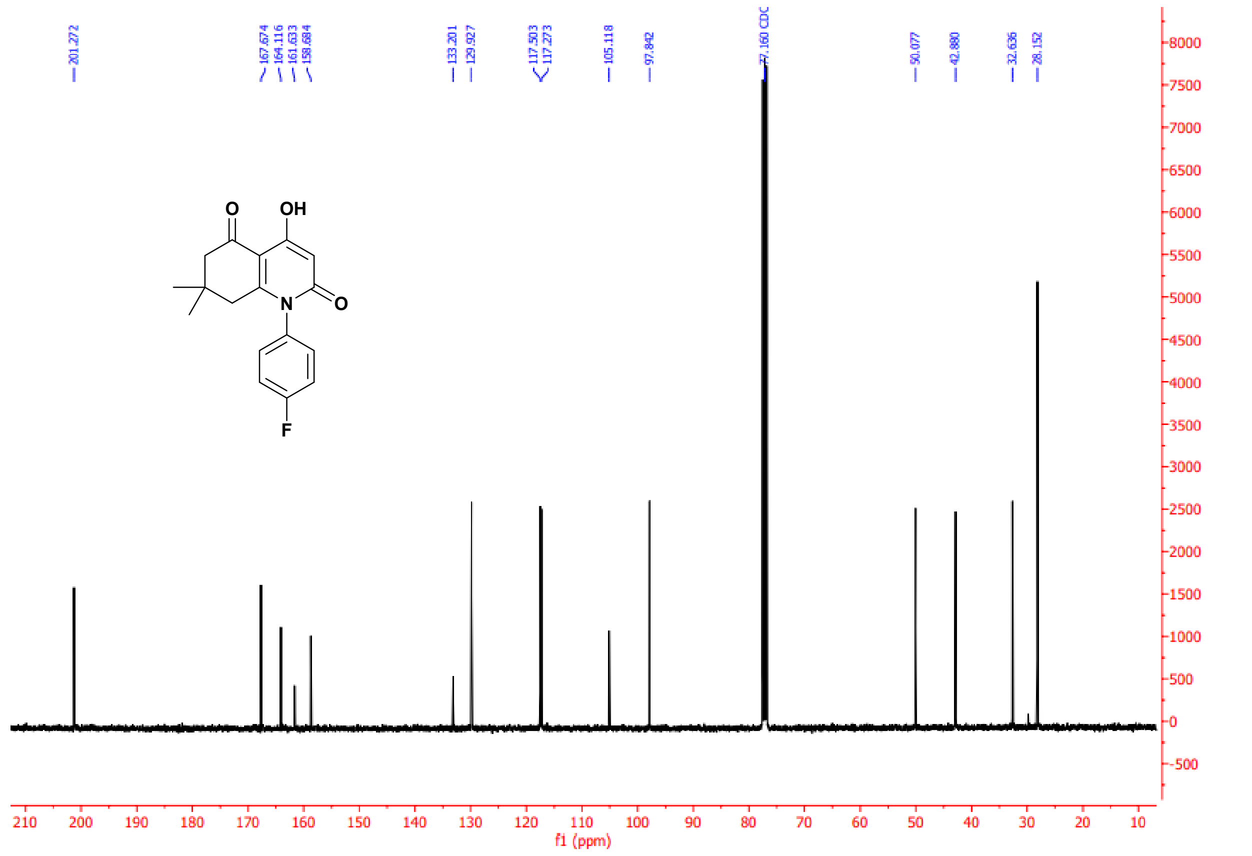

NMR ^{13}C spectrum (101 MHz, $CDCl_3$): 1-(4-fluorophenyl)-4-hydroxy-7,7-dimethyl-7,8-dihydroquinoline-2,5(1H,6H)-dione

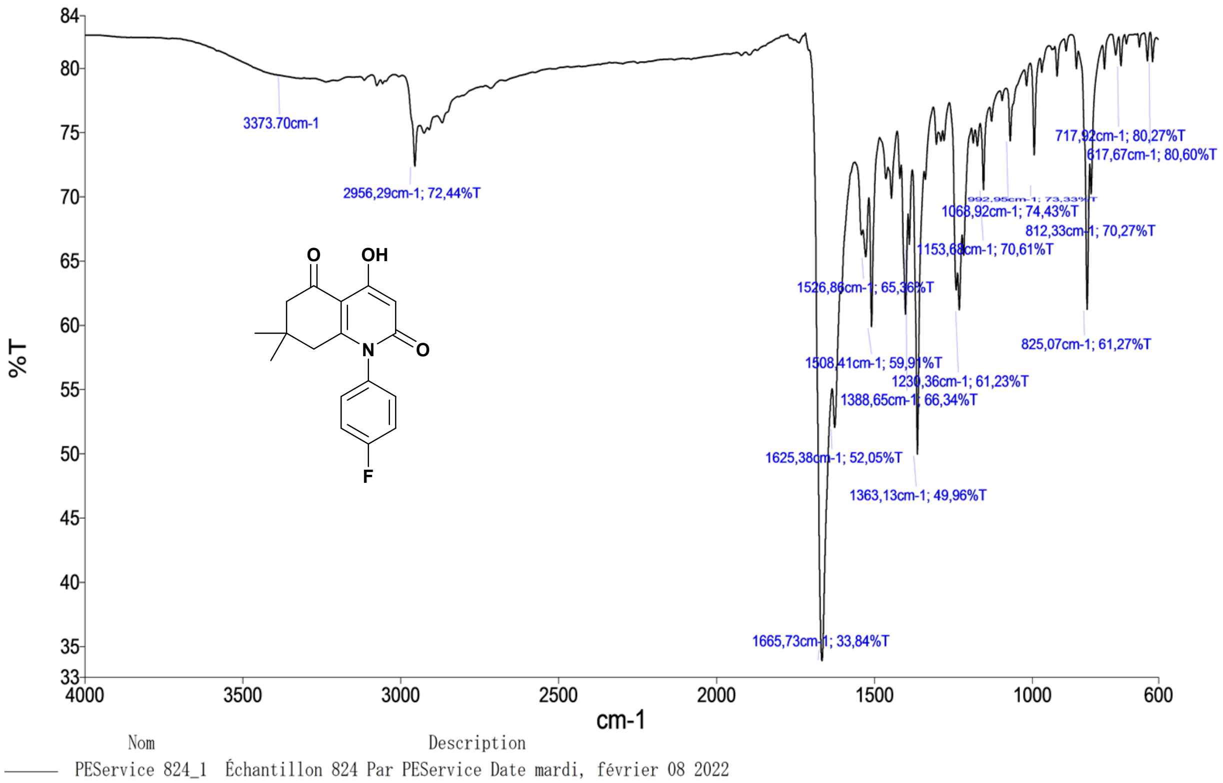

IR spectrum: 1-(4-fluorophenyl)-4-hydroxy-7,7-dimethyl-7,8-dihydroquinoline-2,5(1H,6H)-dione

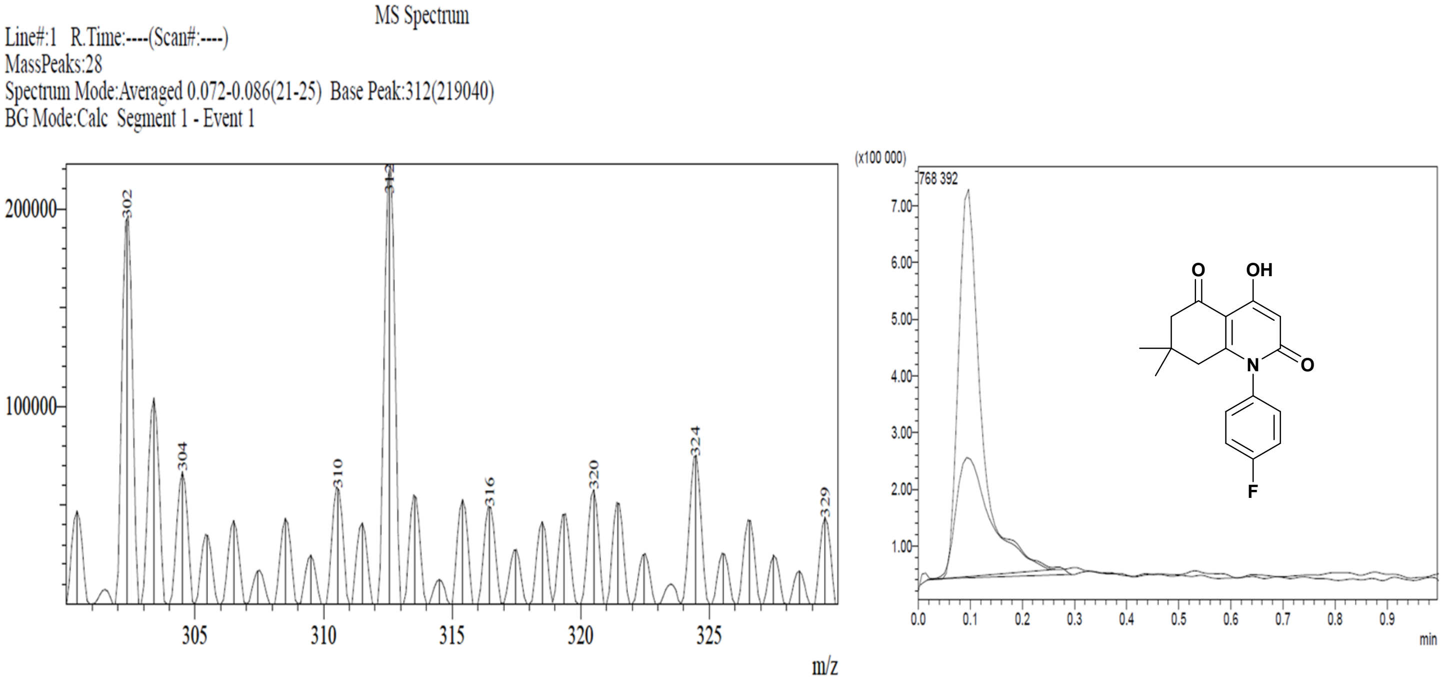

LC-MS spectrum: 1-(4-fluorophenyl)-4-hydroxy-7,7-dimethyl-7,8-dihydroquinoline-2,5(1H,6H)-dione

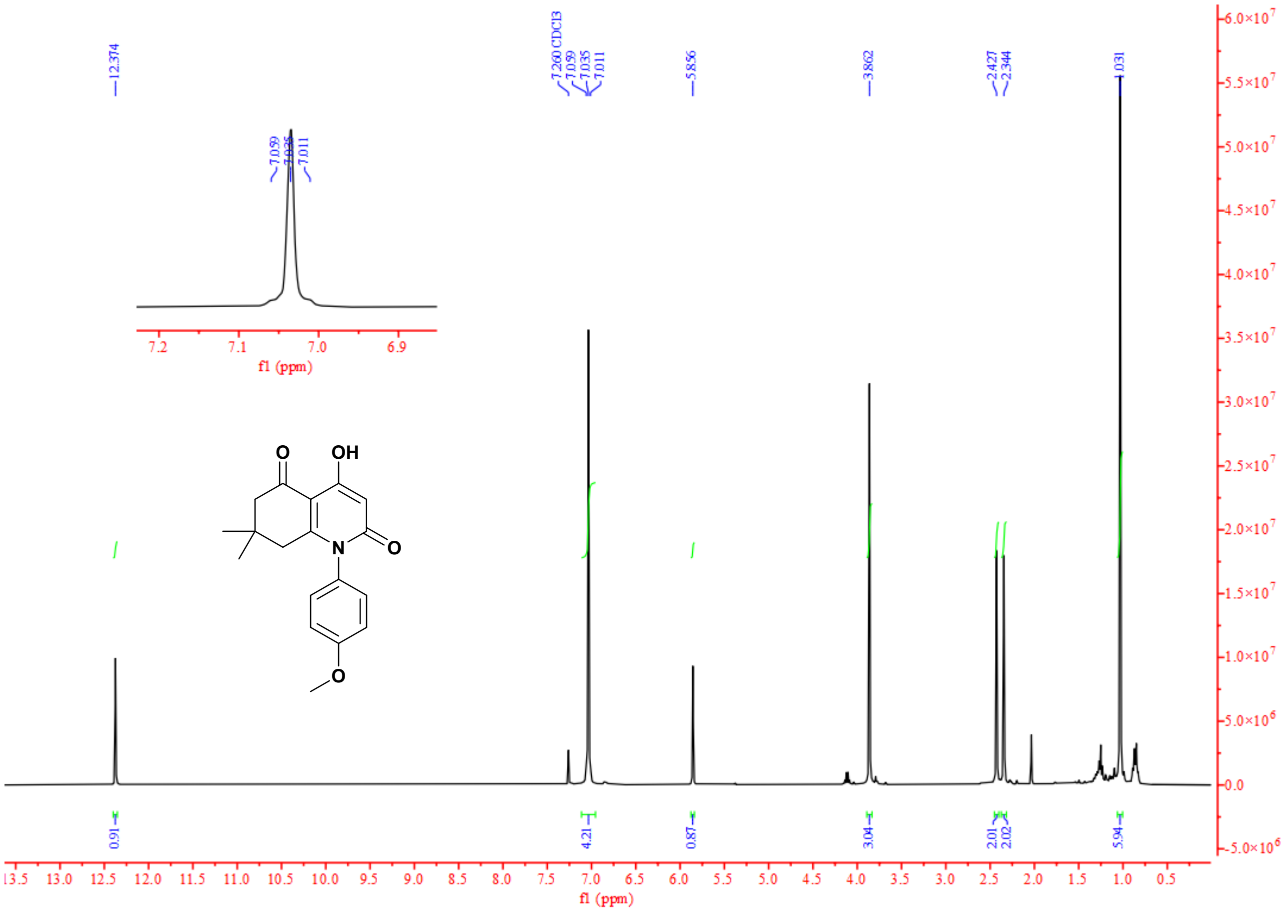

NMR ^{1}H spectrum (400 MHz, $CDCl_3$): 4-hydroxy-1-(4-methoxyphenyl)-7,7-dimethyl-7,8-dihydroquinoline-2,5(1H,6H)-dione

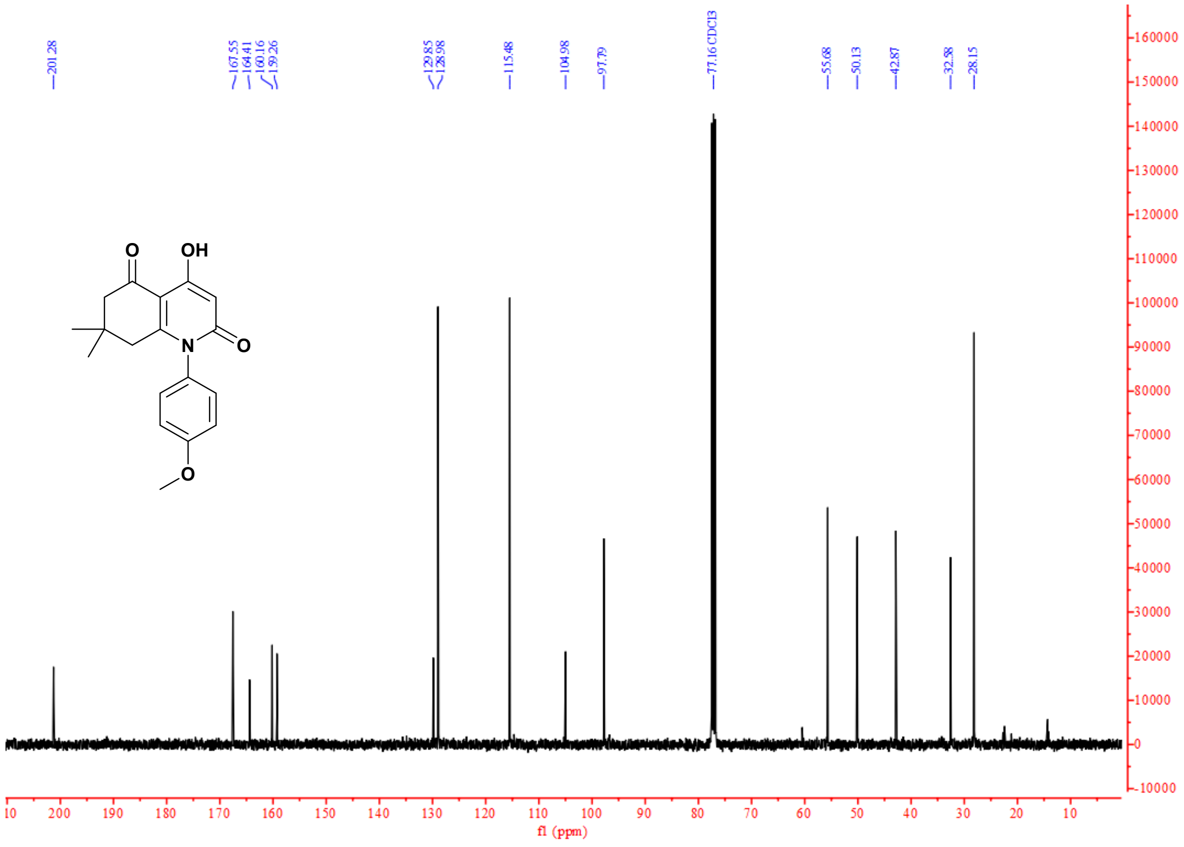

NMR ^{13}C spectrum (101 MHz, $CDCl_3$): 4-hydroxy-1-(4-methoxyphenyl)-7,7-dimethyl-7,8-dihydroquinoline-2,5(1H,6H)-dione

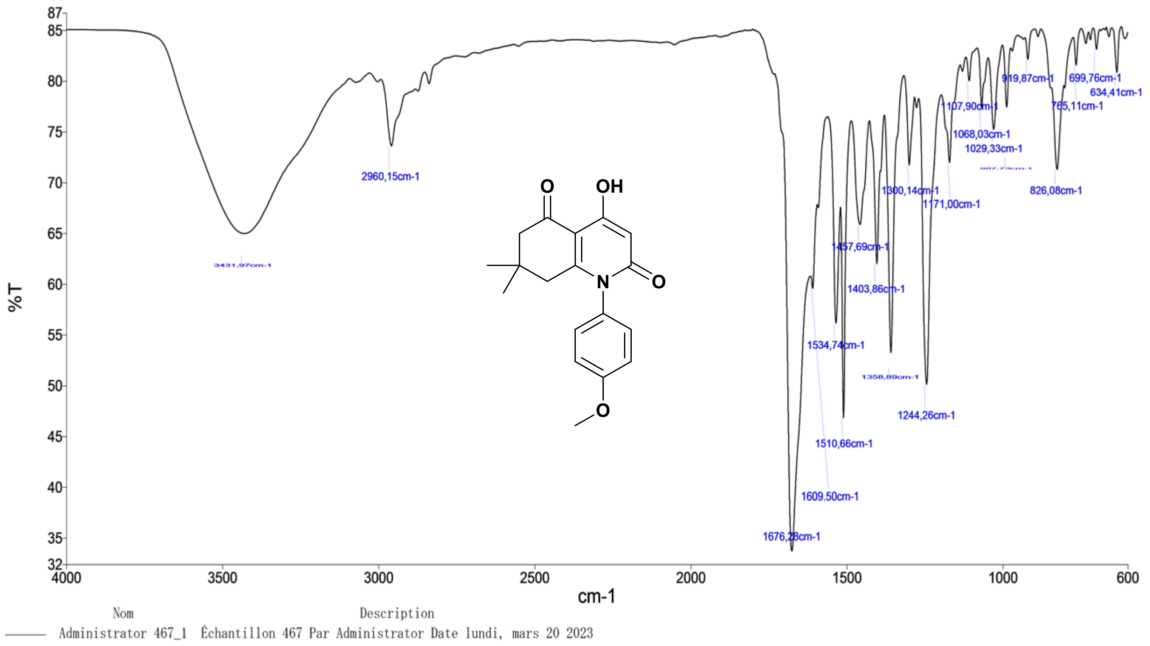

IR spectrum: 4-hydroxy-1-(4-methoxyphenyl)-7,7-dimethyl-7,8-dihydroquinoline-2,5(1H,6H)-dione

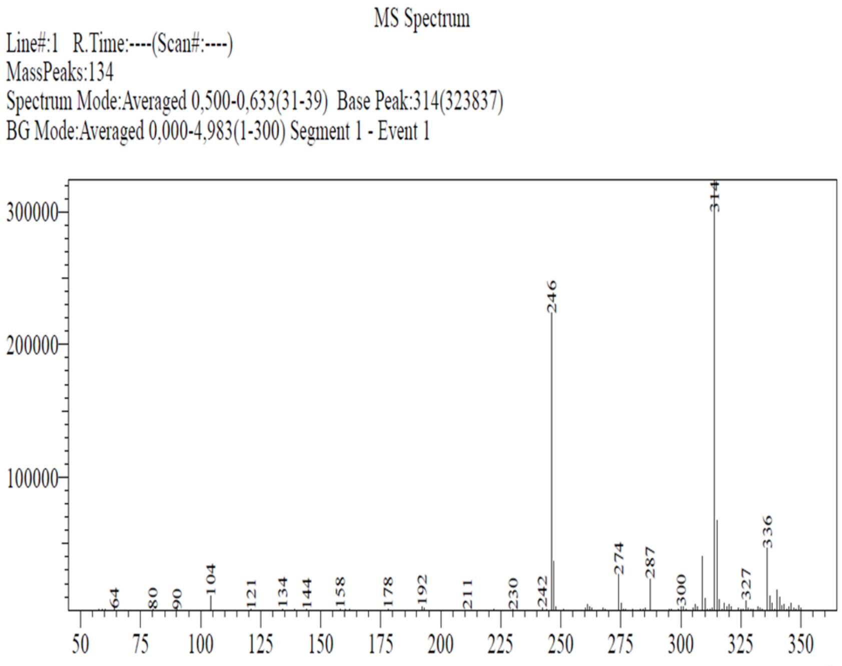

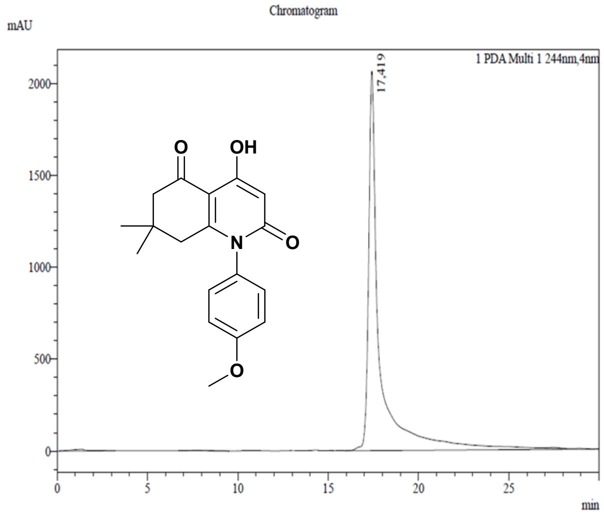

LC-MS spectrum: 4-hydroxy-1-(4-methoxyphenyl)-7,7-dimethyl-7,8-dihydroquinoline-2,5(1H,6H)-dione

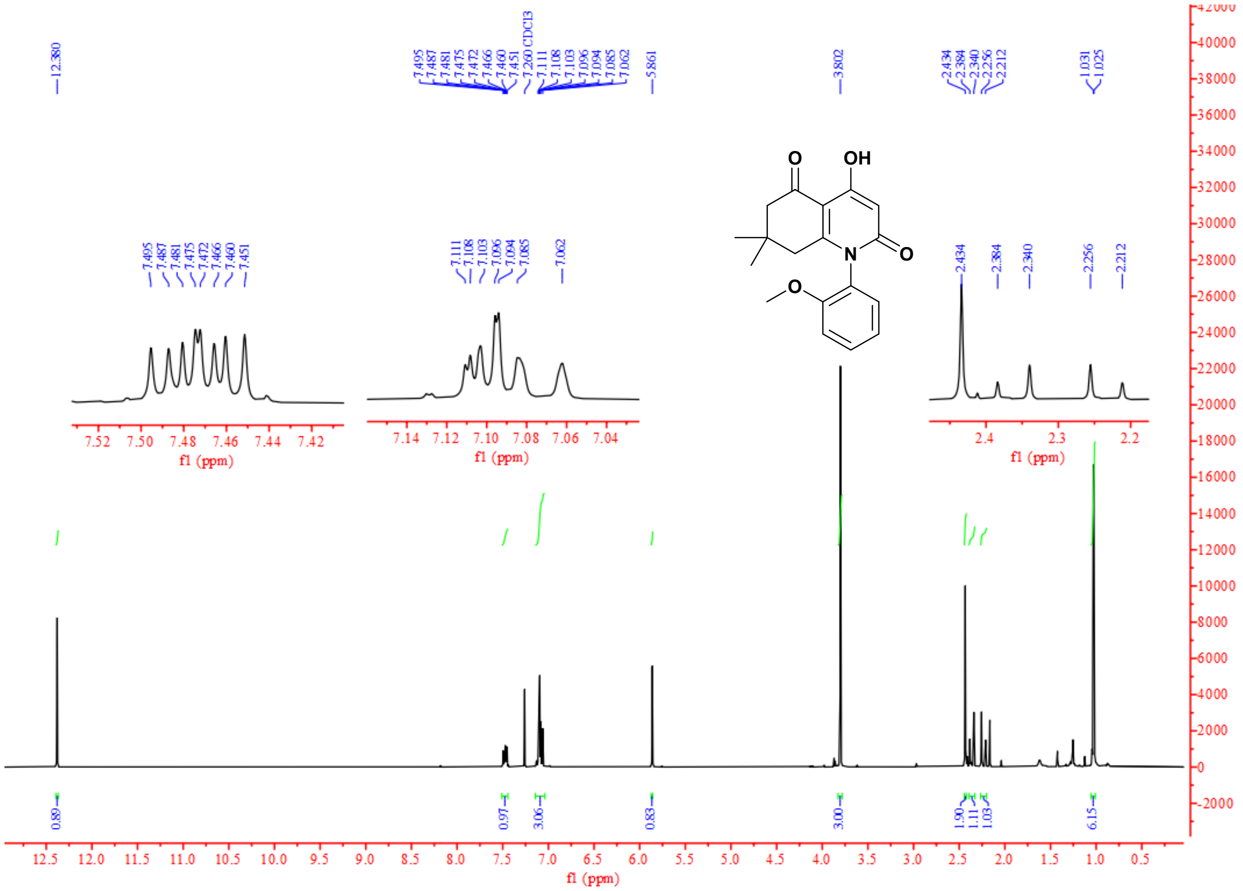

NMR ^{1}H spectrum (400 MHz, $CDCl_3$): 4-hydroxy-1-(2-methoxyphenyl)-7,7-dimethyl-7,8-dihydroquinoline-2,5(1H,6H)-dione

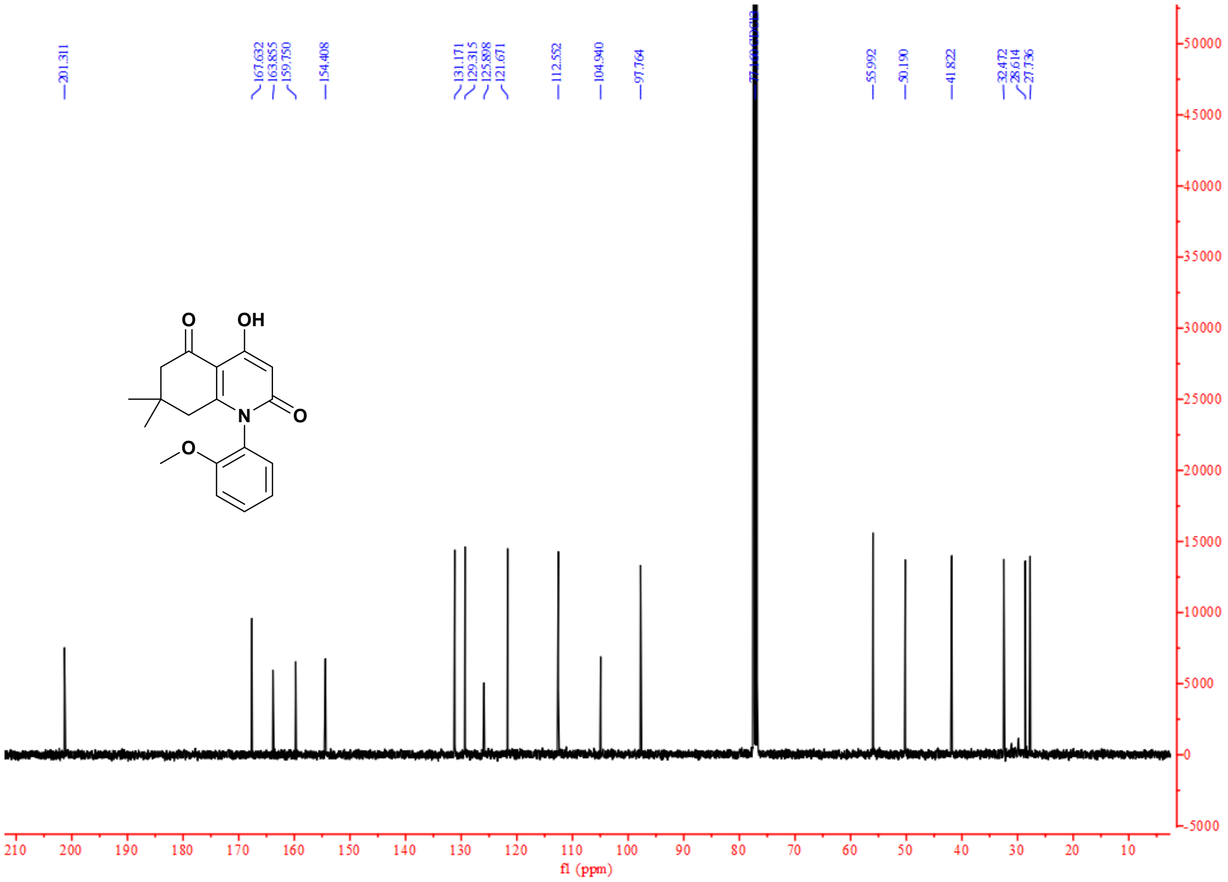

NMR ^{13}C spectrum (101 MHz, $CDCl_3$): 4-hydroxy-1-(2-methoxyphenyl)-7,7-dimethyl-7,8-dihydroquinoline-2,5(1H,6H)-dione

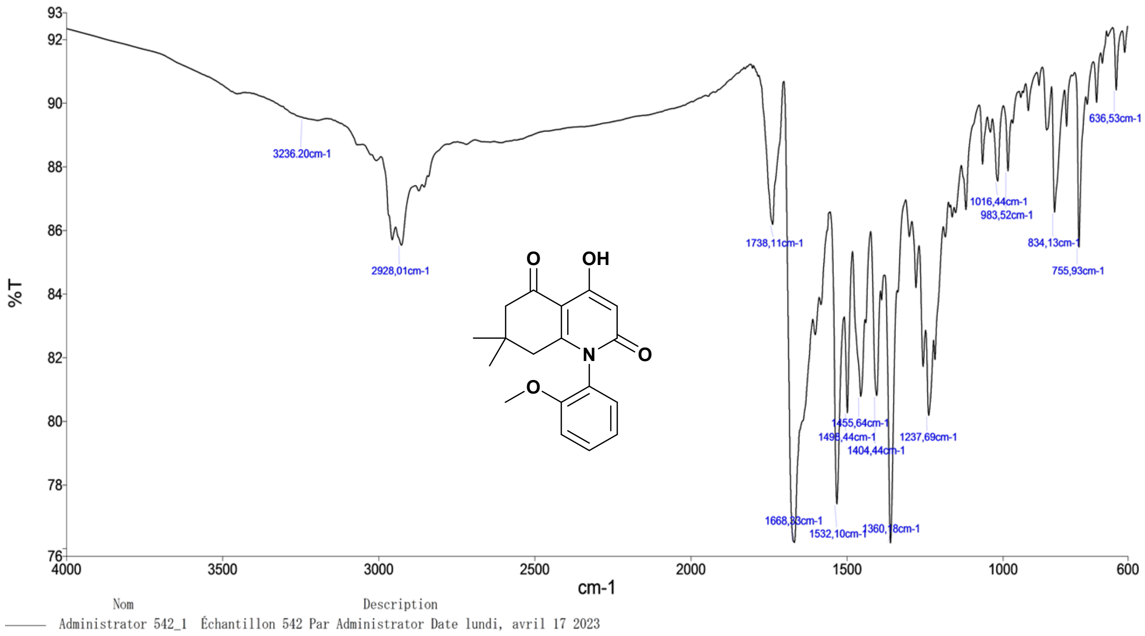

IR spectrum: 4-hydroxy-1-(2-methoxyphenyl)-7,7-dimethyl-7,8-dihydroquinoline-2,5(1H,6H)-dione

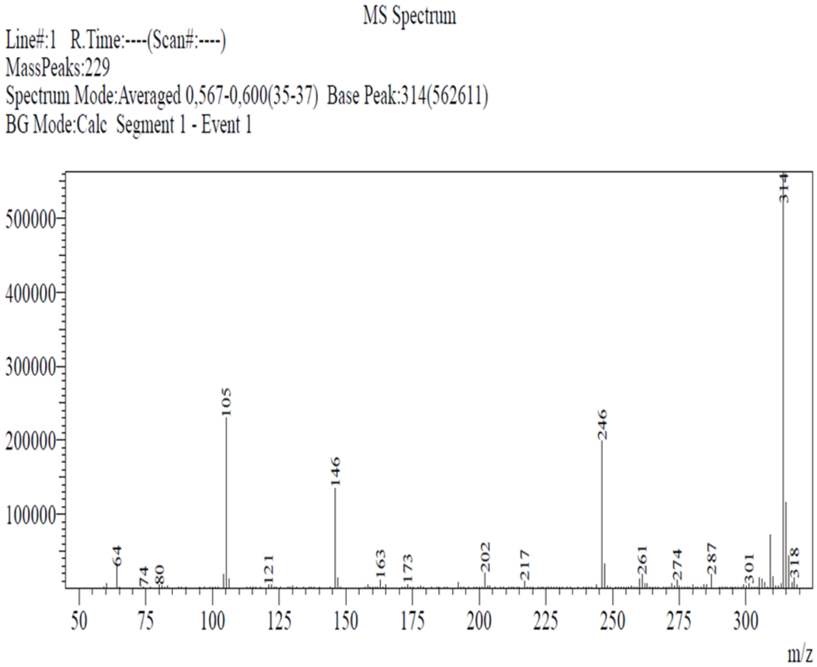

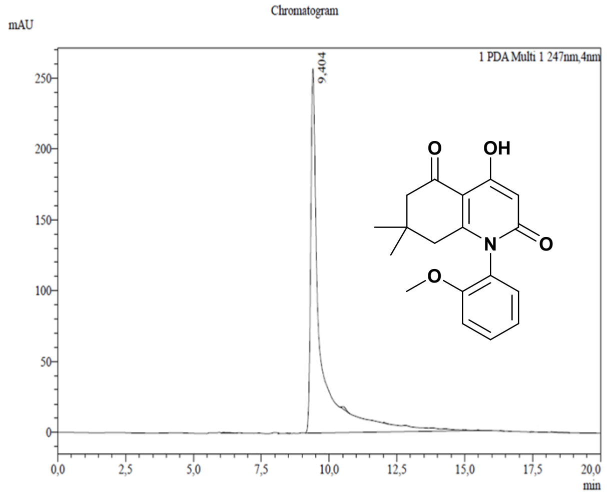

LC-MS spectrum: 4-hydroxy-1-(2-methoxyphenyl)-7,7-dimethyl-7,8-dihydroquinoline-2,5(1H,6H)-dione

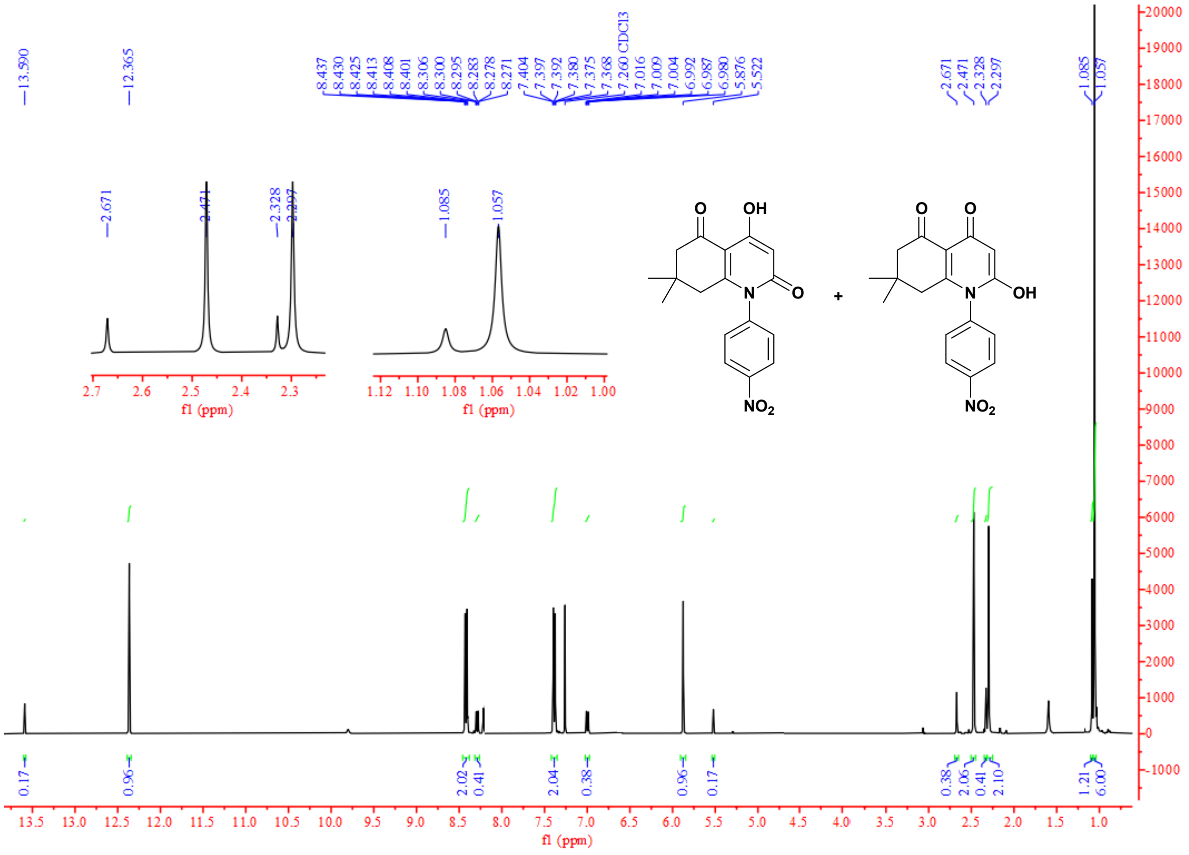

NMR ^{1}H spectrum (400 MHz, $CDCl_3$): 4-hydroxy-7,7-dimethyl-1-(4-nitrophenyl)-7,8-dihydroquinoline-2,5(1H,6H)-dione + 2-hydroxy-7,7-dimethyl-1-(4-nitrophenyl)-7,8-dihydroquinoline-4,5(1H,6H)-dione

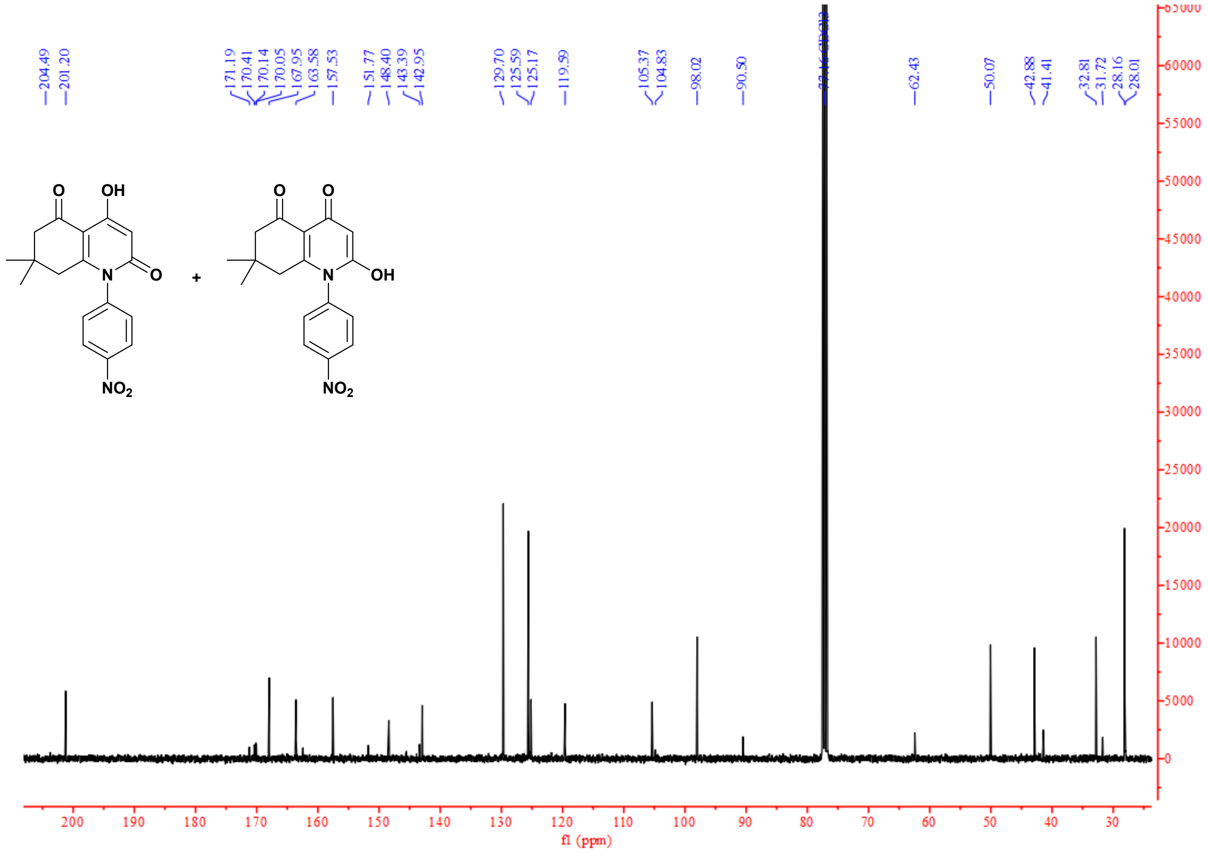

NMR ^{13}C spectrum (101 MHz, $CDCl_3$): 4-hydroxy-7,7-dimethyl-1-(4-nitrophenyl)-7,8-dihydroquinoline-2,5(1H,6H)-dione + 2-hydroxy-7,7-dimethyl-1-(4-nitrophenyl)-7,8-dihydroquinoline-4,5(1H,6H)-dione

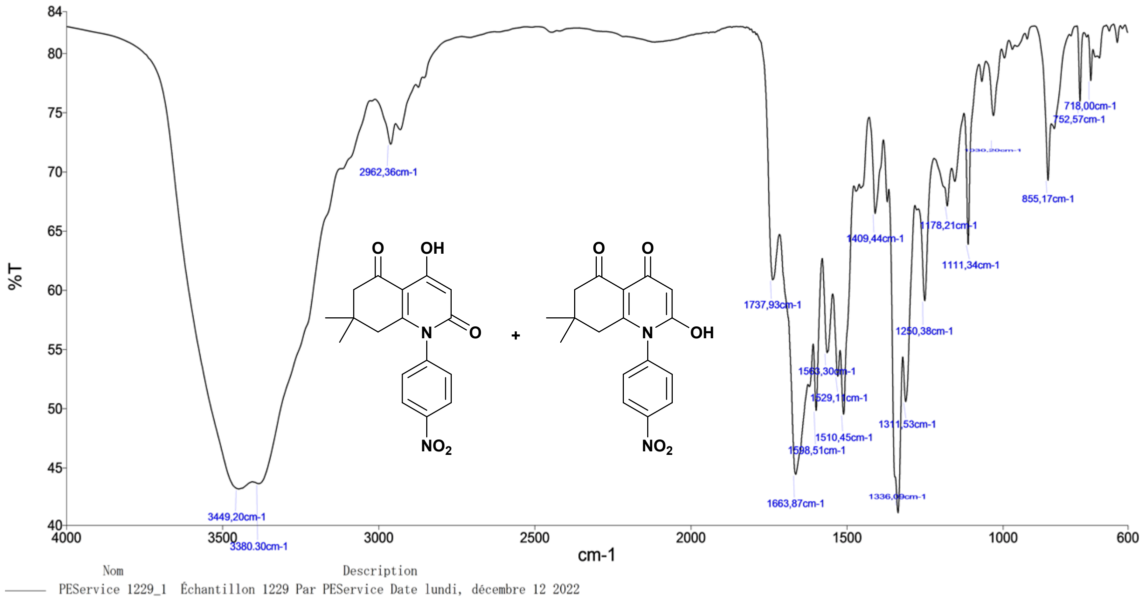

IR spectrum: 4-hydroxy-7,7-dimethyl-1-(4-nitrophenyl)-7,8-dihydroquinoline-2,5(1H,6H)-dione + 2-hydroxy-7,7-dimethyl-1-(4-nitrophenyl)-7,8-dihydroquinoline-4,5(1H,6H)-dione
